# Relationship between serum lipids and the risk of hemorrhagic stroke: a meta-analysis of 50 million participants from prospective cohort studies

**DOI:** 10.1186/s12944-025-02698-0

**Published:** 2025-09-30

**Authors:** Xuefan Yao, Houlin Lai, Kehui Ma, Benke Zhao, Aini He, Wei Sun, Yuan Wang, Haiqing Song

**Affiliations:** 1 Department of Neurology, Xuanwu Hospital, Capital Medical University, No. 45 Changchun St, Beijing, 100053 China; 2 Beijing Stroke Quality Control Center, No. 45 Changchun St, Beijing, 100053 China

**Keywords:** Lipids, Hemorrhagic stroke, Hypolipidemic agents, Meta-analysis.

## Abstract

**Background:**

While prior meta-analyses have demonstrated the benefits of lipid-lowering agents for atherosclerotic cardiovascular disease prevention in high-risk populations with definite risk, the applicability of these findings to global community-based populations, where hemorrhagic risk profiles and optimal serum lipid levels might differ, remains less defined. This gap underscores the need to clarify the relationship between serum lipids and hemorrhagic stroke (HS) risk in a broader community-based population.

**Methods:**

PubMed, Ovid, and the Cochrane Library were searched from inception to May 21, 2025, for observational prospective cohorts in populations with no major disease, baseline serum lipid assessment, and first-attack HS. Risk ratios (RRs) with 95% confidence intervals (95% CIs) of continuous variables and dose-response modeling were both analyzed. Potential nonlinear trends were validated through model comparison.

**Results:**

A total of 50,244,342 participants from 54 cohorts were included. Continuous variable analysis revealed that serum total cholesterol (sTC) was inversely related to total HS (RR = 0.94, 95% CI 0.90 ~ 0.97, *P* < 0.01) and intracerebral hemorrhage (ICH) (RR = 0.90, 95% CI 0.87 ~ 0.93, *P* < 0.01) risk; serum low-density lipoprotein cholesterol (sLDL-C) was inversely related to total HS (RR = 0.92, 95% CI 0.86 ~ 0.98, *P* = 0.012) and ICH (RR = 0.83, 95% CI 0.74 ~ 0.94, *P* < 0.01) risk; and serum high-density lipoprotein cholesterol (sHDL-C) was inversely related to subarachnoid hemorrhage (SAH) (RR = 0.77, 95% CI 0.63 ~ 0.92, *P* < 0.01) risk. In the dose-response analysis, linear trends were detected in total HS, with a decreasing risk of 7.8% (95% CI 3.6%~11.8%, *P* < 0.01) per mmol/L sTC increase and a decreasing risk of 9.6% (95% CI 2.4%~16.2%, *P* < 0.01) per mmol/L sLDL-C increase; in ICH, with a decreasing risk of 17.0% (95% CI 7.5%~25.6%, *P* < 0.01) per mmol/L sLDL-C increase; and in SAH, with a decreasing risk of 20.7% (95% CI 1.5%~36.1%, *P* = 0.036) per mmol/L sHDL-C increase and a decreasing risk of 54.0% (95% CI 5.5%~77.6%, *P* = 0.035) per mmol/L triglyceride increase. U-shaped trends were detected between sTC and ICH risk, with the lowest RR at 6.07 mmol/L; between sLDL-C and SAH risk, with a minimum RR of 3.69 mmol/L; between sHDL-C and total HS risk, with a minimum RR of 1.53 mmol/L; and between sHDL-C and ICH risk, with a minimum RR of 1.50 mmol/L.

**Conclusion:**

When the minimal important difference was set at an RR of 0.87 ~ 1.15, sTC between 3.48 ~ 5.20 mmol/L, sLDL-C between 1.09 ~ 3.40 mmol/L, and sHDL-C between 0.99 ~ 2.34 mmol/L might provide balanced lipid management, contributing to stroke prevention in global community-based populations.

**Trial registration:**

The protocol of this study was registered on PROSPERO (CRD420250650371).

**Supplementary Information:**

The online version contains supplementary material available at 10.1186/s12944-025-02698-0.

## Introduction

Stroke remains a global health challenge, with 12.22 million cases recorded in the Global Burden of Diseases (GBD) in 2019, of which 7.63 million were ischemic stroke (IS) and 4.59 million were hemorrhagic stroke (HS) [1]. Concerns regarding the disease burden have led to the increasing adoption of lipid-lowering agents for stroke prevention. While serum lipids are well-established targets for atherosclerotic cardiovascular disease (ASCVD) prevention, most evidence is based on populations with definite ASCVD risk [2–6]. Excessively premature or intensive lipid-lowering interventions in broader community-based populations with distinct hemorrhagic risk profiles and optimal serum lipid levels might lead to overtreatment and HS risk [7–10]. Furthermore, lipid management extends beyond lipid-lowering agents, necessitating a heightened focus on the intrinsic relationship between serum lipid profiles and HS risk.

Prior meta-analyses have focused predominantly on specific serum lipid components, where three studies targeted serum low-density lipoprotein cholesterol (sLDL-C) [11–13], whereas one revealed divergent correlations for serum high-density lipoprotein cholesterol (sHDL-C) between intracerebral hemorrhage (ICH) and subarachnoid hemorrhage (SAH) [14]. Wang et al. pioneered dose-response analyses (DRA) for multiple serum lipids but detected only a nonlinear trend between serum total cholesterol (sTC) and HS [15], whereas Jin et al. further confirmed nonlinear trends between sHDL-C and HS [16]. However, both studies showed limited interpretability for reliance on highest-versus-lowest comparisons, in response to which Gong et al. employed continuous variable analysis (CVA) for nonlinear trend detection [17]. However, all these studies lacked statistical comparisons between different model fits and were thus unable to make selections between linear and nonlinear models. Moreover, the relationship between sLDL-C and HS remains unclear, likely due to intrinsic heterogeneity, as the etiological distinctions between ICH and SAH necessitate stratified analyses of HS subtypes.

To address these gaps, this study hypothesized a nonlinear relationship between serum lipid levels and HS risk in a broader community-based population, with subtype-specific variations. This meta-analysis aimed to analyze the relationship between serum lipids and HS risk in community-based populations, as well as HS subtypes. Potential nonlinear trends were validated through model fit comparisons.

## Method

The criteria of the Preferred Reporting Items for Systematic Reviews and Meta-Analyses (PRISMA) were applied in this meta-analysis [18]. The PRISMA 2020 checklist is presented in Supplemental Table 1. The protocol for this systematic review was registered on the International Prospective Register of Systematic Reviews (PROSPERO) (CRD420250650371).

### Inclusion criteria

Cohorts in populations with no major disease and baseline serum lipid assessments were included. Studies on populations with diabetes mellitus, chronic kidney disease, ASCVD, during pregnancy, or maintenance hemodialysis were excluded, but populations with hypertension, obesity, tobacco dependence, and hyperlipidemia were included because of their high prevalence in both community-based populations and patients with HS. Studies with no exception of previous HS were also included, with a lower assessment of reliability.

Prospective cohort studies were included in the analysis, which required serum samples collected prior to follow-up initiation. Both nested case-control and retrospective studies were excluded to minimize potential bias. The methods of studies included observation, thus studies with experimental interventions on serum lipids were excluded. However, studies with spontaneous initiation of lipid-lowering therapies during follow-up were included. The confounding factor of statin therapy was either adjusted or excluded in the analysis to alleviate its impact on the outcome.

Baseline serum lipids were required for the assessment of exposure, where sTC, serum triglyceride (sTG), sHDL-C, and sLDL-C were all acceptable classifications in the analysis. Participants were categorized into two or more distinct ranges of serum lipids.

The outcome was defined as first-attack HS, including ICH and SAH, with both incidence and mortality acceptable. A reliable diagnosis was confirmed by experienced neurologists on the basis of the International Classification of Diseases (ICD), 9th or 10th Revision.

The relative risk (RR), odds ratio (OR), or hazard ratio (HR) with the corresponding 95% confidence interval (95% CI) indicated acceptable therapeutic effects.

### Search strategy and selection criteria

Embase, Ovid, and the Cochrane Library were searched for studies on serum lipids and HS published before May 21, 2025. Duplicates were removed with EndNote20 and Zotero. The full search strategies used for each database are detailed in Supplemental Table 2.

Eligible studies were further reviewed with the full texts and supplementary materials and were excluded if they had: (1) no desired classification for lipids, such as non-high-density lipoprotein cholesterol (non-HDL-C) or sTG/sHDL-C; (2) no specific classification for hemorrhagic stroke; (3) no available exposure dose classification; or (4) no therapeutic effect or 95% CI reported. Publications on prospective cohorts were excluded when a better version was available.

### Data extraction

Basic information and results were extracted by two independent reviewers, with discrepancies resolved through discussion with a third reviewer. Basic information was sorted into study, population (race or geographic location), number, follow-up, sex (proportion of males), age, confounding variables, effect size, exposure, fasting state, and endpoint. The results were sorted into the following categories: study, exposure, categories (ranges or categories for levels of exposure), dose (the symbolic dose for the category), end (types of HS), case (number of outcomes), risk (population or person-years at risk), and effect size with 95% CI.

For studies reporting dose ranges at baseline, exposure doses were calculated by assigning the midpoint or mean of each category. The open-ended terminal categories were assumed to have the same range width as the closest adjacent category. This method was also applied to terminal categories with an extreme ending (making the terminal range triple as the closest adjacent category). The value of the continuous variable at the exposure level was negated when it decreased. For studies that provided different but similar ranges between genders, the midpoints were used as ranges for both genders.

### Quality assessment

Quality was assessed with the Newcastle-Ottawa Scale (NOS) by two independent reviewers, with discrepancies resolved through discussion with a third reviewer [19]. The reliability of the agreement among multiple assessors was assessed by Fleiss’ kappa statistics (poor, slight, fair, substantial, and perfect at 0, 0.2, 0.4, 0.6, and 0.8, respectively) [20].

Specifically, populations with hypertension, obesity, tobacco addiction, or hyperlipidemia were regarded as having reduced representativeness. Populations with no exception of the previous HS were regarded as failing to demonstrate the absence of an outcome at baseline. Comparability was rated as high (score = 2) if adjusted for 5 or more confounders, medium (score = 1) for less than 5 confounders, and low (score = 0) if unadjusted. Adequate follow-up required a duration of 10 years or longer and a loss of 20% or less. The exposure ascertainment was downgraded for an unspecified state of fasting when serum sampling was performed. Studies were regarded as high for a total score of 8 or more, medium for a total score of 7, and low for a total score of 6 or lower.

### Statistical analysis

RR per mmol/L increase was calculated by generalized least squares regression and then pooled into a random-effects model (REM) [21, 22]. Heterogeneity was quantified with I^2^ statistics (no, low, moderate, and high at 0%, 25%, 50%, and 75%, respectively) and Cochran’s Q tests (significant to *P* < 0.05) [23, 24]. Further analyses were conducted when 5 or more studies were pooled, with a Baujat plot for heterogeneity detection [25], Egger’s test (significant to *P* < 0.05), the trim-and-fill method for bias on publication, and one-by-one exclusion for influence from a single study.

Subgroup analysis explored heterogeneity and interactions across population origin, sample size, mean follow-up duration, male proportion, mean age, endpoint type, study quality, and confounding variables for adjustment. Subgroups were considered sources of heterogeneity when no significant heterogeneity within a subgroup was identified or as potential interactions when significant differences between subgroups were detected. Meta-regression was used to explore the temporal trends, as well as associations between study characteristics and therapeutic effects. Characteristics with *P* < 0.10 in the univariate regression were included in the multivariate regression.

Certainty was assessed by the Grading of Recommendations Assessment, Development and Evaluation (GRADE) pro Guideline Development Tool (GDT) system with five major indices (study design, risk of bias, inconsistency, indirectness, and imprecision) and four other considerations (publication bias, large effect, plausible confounding, and dose-response gradient) [26]. The risk of bias was assessed by the average NOS score of the studies included, with a score of 8 or more indicating “not serious bias”, a score of 7 indicating “serious bias”, and a score of 6 or lower indicating “very serious bias”. Inconsistency was assessed by the heterogeneity detected in the REM and was regarded as “no” when no or low heterogeneity was detected, “serious” when moderate heterogeneity was detected, and “very serious” when high heterogeneity was detected. Plausible residual confounding was assessed by multivariate meta-regression and regarded as “no” when no significant variable was detected, “would reduce demonstrated effect” when any significant variable was detected with significant therapeutic effect demonstrated, and “would suggest spurious effect” when any significant variable was detected with no significant therapeutic effect demonstrated. Publication bias was assessed by Egger’s test and considered “strongly suspected” when significant publication bias was detected or “unlikely” otherwise. Imprecision was assessed by the sensitivity analysis and regarded as “no” when the result was stable, “serious” when it was significantly influenced by a single study, and “very serious” when it was significantly influenced by several studies. The GRADEpro GDT system automatically generated certainty after the indices and considerations were filled.

DRAs assessed both linear (Model 1) and nonlinear (Model 2) trends with restricted maximum likelihood models [27]. Covariances between studies were calculated by the Greenland and Longnecker methods for trends (significant at *P* < 0.05) [28, 29]. Nonlinear trends were built on 3-knot splines at 10%, 50%, and 90%, with nonlinear trends estimated by the Wald test by testing the linear hypothesis that the coefficient of the second spline was equal to 0 (significant to *P* < 0.05) [30, 31]. Model fit was evaluated by log-likelihood (Loglik), where a model with a smaller absolute value of Loglik was regarded as a better fit if both models showed statistical significance in the aforementioned tests [28].

Statistical analyses were performed with R (version 4.2.2) and R Studio (version 2022.12.0 + 353). Random models were built by the “metagen” function in the “meta” package (version 6.0–0). Both linear and nonlinear trends were estimated by the “dosresmeta” function in the “dosresmeta” package (version 2.0.1). The nonlinear trends were built by the “rcs” function in the “rms” package (version 6.3-0) [32].

## Results

### Study characteristics

**Fig. 1:**
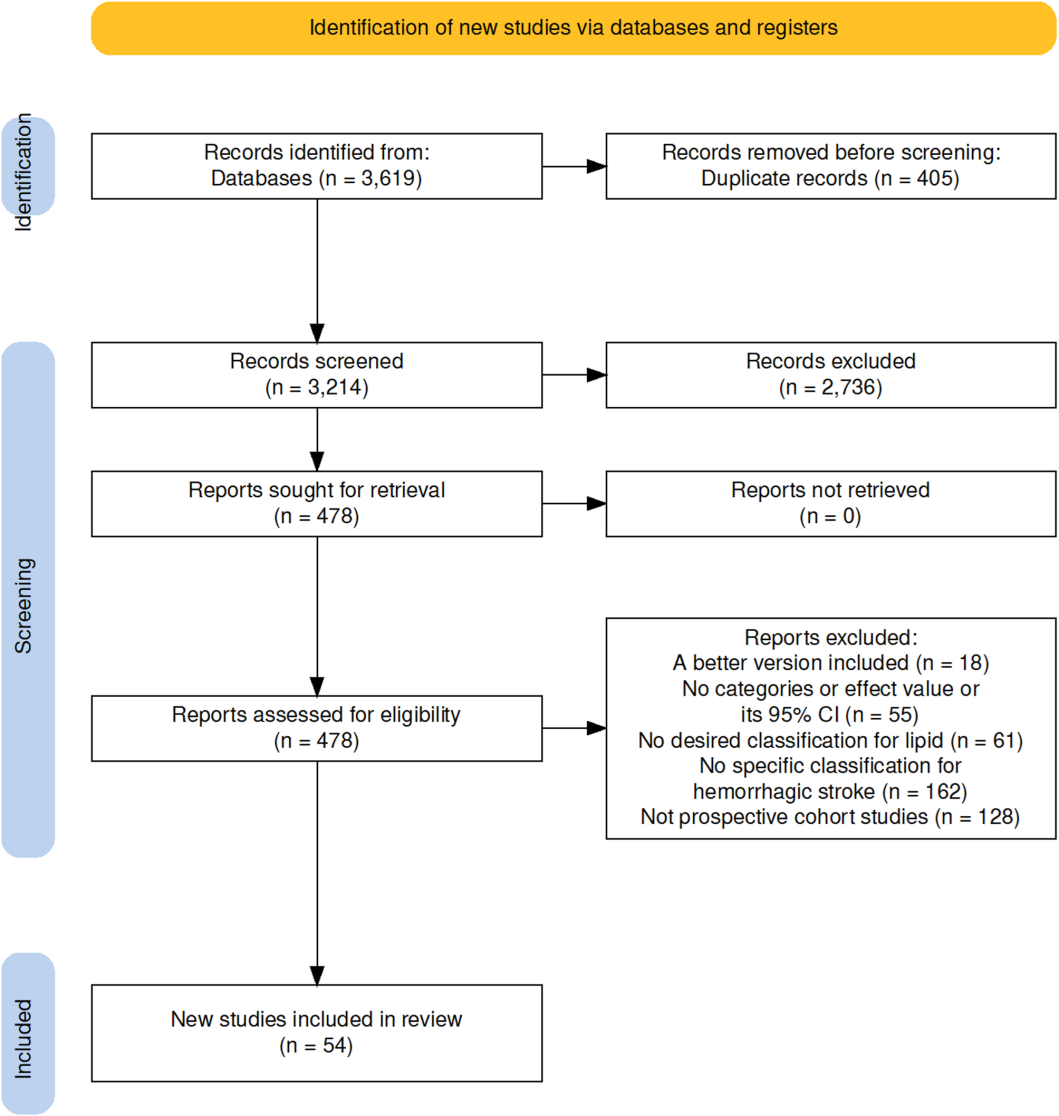
Flow diagrams of the study search and selection

Of 3,619 publications obtained, a total of 50,244,342 participants from 54 cohorts were included (Fig. 1) [33–86], with sTC measured in 39 cohorts, sLDL-C in 18 cohorts, sHDL-C in 23 cohorts, and sTG in 18 cohorts. Among subtypes of HS, ICH was reported in 27 cohorts, and SAH was reported in 17 cohorts (Supplemental Table 3).

### Quality assessment

Forty-one studies were categorized as “high quality”, 10 studies were categorized as “medium quality”, and 3 studies were categorized as “low quality”, with perfect agreement from assessors (κ_NOS score_ = 0.831, κ_NOS category_ = 0.801; Supplemental Table 4). Three studies were downgraded for reduced representativeness of exposure, 15 studies for reduced ascertainment of exposure, 5 for inadequate demonstration of chronological order, 8 for reduced comparability, 1 for unprofessional assessment of outcome, 18 for inadequate follow-up duration, and 4 for inadequate follow-up loss (Supplemental Table 5).

### Serum total cholesterol

#### Continuous variable analysis

**Fig. 2:**
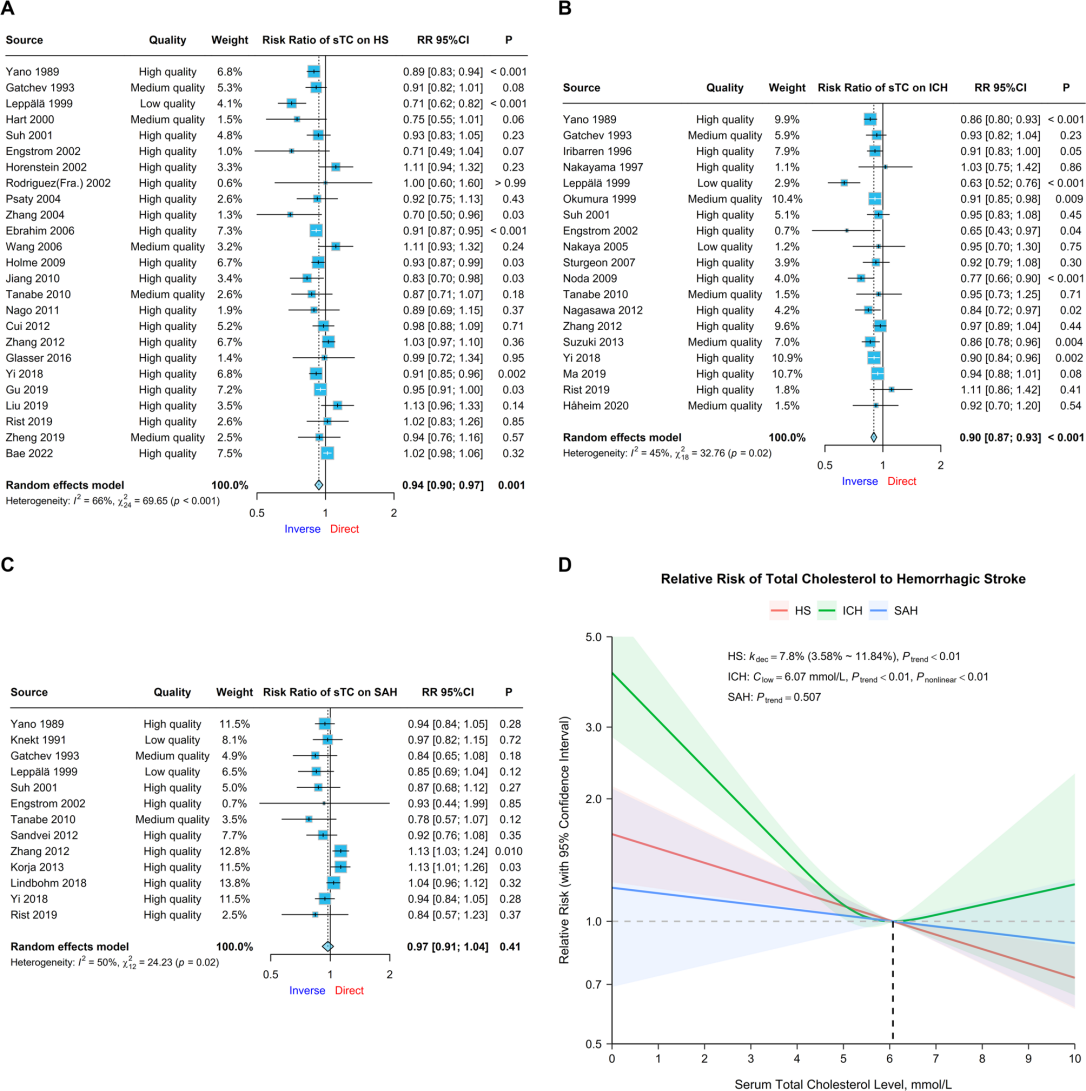
Relationships between serum total cholesterol and hemorrhagic stroke risk **A ~ C** Forest plots of continuous variable analysis of the relative risk of serum total cholesterol on total hemorrhagic stroke, intracerebral hemorrhage, and subarachnoid hemorrhage. Square sizes correspond to cohort weights, horizontal Lines represent 95% confidence intervals, intersection points of crosses represent individual treatment estimates, and diamonds represent pooled treatment estimates. **D** Dose-response analyses between serum total cholesterol and hemorrhagic stroke. Solid Lines represent relative risk trends with changes in serum total cholesterol concentration, semitransparent shadings represent 95% confidence intervals, *k*_dec_ represents the percent relative risk decreasing per mmol/L increase, *C*_low_ represents the serum total cholesterol concentration with the lowest relative risk in a nonlinear relationship, *P*_trend_ represents the *P* value for the trend test in either the linear or nonlinear model, and *P*_nonlinear_ represents the *P* value for Wald’s test in the nonlinear model

Data from 25 cohorts were pooled for CVA on sTC and total HS risk [33, 35, 38, 40–46, 48, 49, 52, 56–58, 60, 63, 67, 71, 73, 74, 76, 77, 81]. An inverse correlation was detected (RR = 0.94, 95% CI 0.90 ~ 0.97, *P* < 0.01), with moderate heterogeneity (I^2^ = 65.54%, *P* < 0.01; Fig. 2A), which attenuated over time (RR = 0.00, 95% CI 0.00 ~ 0.01, *P* = 0.019) (Supplemental Fig. 1). Multivariable regression revealed an increase in the inverse correlation with increasing male proportion and older population age, although the reduction in the demonstrated effect was limited. Subgroup analysis revealed significant interactions among male sex proportion, lipid-lowering therapy adjustment, and quality level, although residual heterogeneity persisted across all subgroups (Table 1).

**Table 1:** Summary of key findings from continuous variable analysis

Expose	End	Number	RR (95% CI)	*P*	I^2^	*P*_Eager_	Confounders in Multivariate Meta-regression	Subgroup as Sources of Heterogeneity	Subgroup as Potential Interaction	Certainty
sTC	Total HS	25	0.94 (0.90~0.97)	<0.01	65.54% (Moderate)	0.285	Male proportion, RR=0.9979 (0.9967~0.9992) Population age, RR=0.9927 (0.9883~0.9971)	None	Male proportion (*P*=0.011) Lipid-lowering therapy adjustment (*P*<0.01) Quality level (*P*<0.01)	Low
	ICH	19	0.90 (0.87~0.93)	<0.01	45.06% (No)	0.422	Unspecified state of fasting (vs. Fasting state), RR=0.9195 (0.8578~0.9857)	None	None	Moderate
	SAH	13	0.97 (0.91~1.04)	0.414	50.47% (Moderate)	0.017	Population age, RR=0.9905 (0.9820~0.9991)	Follow-up duration Population age Lipid-lowering adjustment	Population age (*P*<0.01) Lipid-lowering adjustment (*P*=0.024)	Very low
sLDL-C	Total HS	12	0.92 (0.86~0.98)	0.012	79.65% (High)	0.054	Endpoint of mortality (vs. incidence), RR=0.8688 (0.8186~0.9222)	Endpoint type	State of fasting (*P*=0.014) Endpoint type (*P*<0.01)	Very low
	ICH	9	0.83 (0.74~0.94)	<0.01	64.96% (Moderate)	0.843	None	Male proportion	Population source (*P*=0.018) Population size (*P*=0.023)	Low
	SAH	4	0.94 (0.82~1.07)	0.329	78.94% (High)	-	-	-	-	-
sHDL-C	Total HS	14	0.95 (0.82~1.10)	0.466	76.18% (High)	0.538	None	None	State of fasting (*P*=0.038)	Very low
	ICH	14	0.96 (0.80~1.16)	0.703	67.13% (Moderate)	0.488	None	None	None	Very low
	SAH	8	0.77 (0.63~0.92)	<0.01	74.87% (Moderate)	0.629	Follow-up duration, RR=0.9751 (0.9652~0.9851) NOS, RR=1.3070 (1.0358~1.6491)	Population source Follow-up duration Physical exercise adjustment	-	Moderate
sTG	Total HS	10	1.00 (0.99~1.01)	0.847	48.50% (No)	0.119	-	Population age Alcohol abuse adjustment BMI adjustment Anti-hypertensive drug adjustment	Population source (*P*<0.01) Population age (*P*=0.028)	Low
	ICH	9	0.86 (0.73~1.02)	0.091	63.28% (Moderate)	0.082	Asian Population (vs. American), RR=1.7518 (1.2973~2.3655)	Population source	Population source (*P*=0.010)	Very low
	SAH	2	0.74 (0.35~1.55)	0.422	75.48% (High)	-	-	-	-	-

Data on ICH were obtained from 19 cohorts, revealing an inverse correlation (RR = 0.90, 95% CI 0.87 ~ 0.93, *P* < 0.01) with low heterogeneity (I^2^ = 45.06%, *P* = 0.018; Fig. 2B) [33, 35–39, 41, 42, 47, 50, 54, 57, 61, 63, 65, 71, 75, 76, 78]. Compared with the fasting state, multivariable regression revealed an increase in inverse correlation with the unspecified state of fasting when serum was collected, but subgroup analysis revealed no significant interaction (Table 1).

Data on SAH were obtained from 13 cohorts, with no significant correlation (RR = 0.97, 95% CI 0.91 ~ 1.04, *P* = 0.414) and high heterogeneity (I^2^ = 50.47%, *P* = 0.019; Fig. 2C) [33–35, 38, 41, 42, 57, 62–64, 70, 71, 76]. Publication bias was detected (*P* = 0.017). Multivariable regression revealed an increase in inverse effects from older population age. Subgroup analysis revealed a significant interaction effect of population age and lipid-lowering adjustment, with potential reductions in residual heterogeneity from subgrouping by follow-up duration, population age, and lipid-lowering adjustment (Table 1).

#### Dose-response analysis

DRA in 20 cohorts revealed significant trends between sTC and total HS risk in both models, whereas the Wald test revealed an insufficient nonlinear trend in the nonlinear model (Model 2, *P* = 0.201). The linear model (Model 1) was the better fit (logLik = 11.574), with a decreasing risk of 7.8% (95% CI 3.6%~11.8%, *P* < 0.01) per mmol/L sTC increase (Fig. 2D; Table 2).

**Table 2:** Summary of key findings from dose-response analyses

Expose	End	Number	Better Fitting Model	I^2^	logLik	Decreasing Risk per mmol/L Serum Lipid Increasing	Non-linear Shape
sTC	Total HS	20	Linear Model (Model 1)	64.6%	11.574	7.80% (3.58%~11.84%)	-
	ICH	15	Non-linear Model (Model 2)	53.3%	0.919	-	U-shaped, lowest at 6.07 mmol/L
	SAH	10	None	-	-	-	-
sLDL-C	Total HS	9	Linear Model (Model 1)	86.6%	4.729	9.58% (2.43%~16.22%)	-
	ICH	9	Linear Model (Model 1)	67.6%	0.067	17.03% (7.45%~25.61%)	-
	SAH	3	Non-linear Model (Model 2)	0%	0.549	-	U-shaped, lowest at 3.69 mmol/L
sHDL-C	Total HS	11	Non-linear Model (Model 2)	62.4%	−16.596	-	U-shaped, lowest at 1.53 mmol/L
	ICH	13	Non-linear Model (Model 2)	49.0%	−26.750	-	U-shaped, lowest at 1.50 mmol/L
	SAH	6	Linear Model (Model 1)	29.0%	−2.805	20.68% (1.50%~36.12%)	-
sTG	Total HS	7	None	-	-	-	-
	ICH	8	None	-	-	-	-
	SAH	1	Linear Model (Model 1)	-	−0.657	54.03% (5.50%~77.64%)	-

DRA in 15 cohorts revealed significant trends between sTC and ICH risk in both models. A nonlinear trend was demonstrated in the nonlinear model (Model 2, *P* < 0.01), which was also a better fit (logLik = 0.919), with a U-shaped trend of the lowest RR at 6.07 mmol/L, but the increasing risk was not as significant for sTC levels over 6.07 mmol/L (Fig. 2D; Table 2).

DRA in 10 cohorts revealed no significant trend between sTC and SAH risk.

### Serum low-density lipoprotein cholesterol

#### Continuous variable analysis

**Fig. 3:**
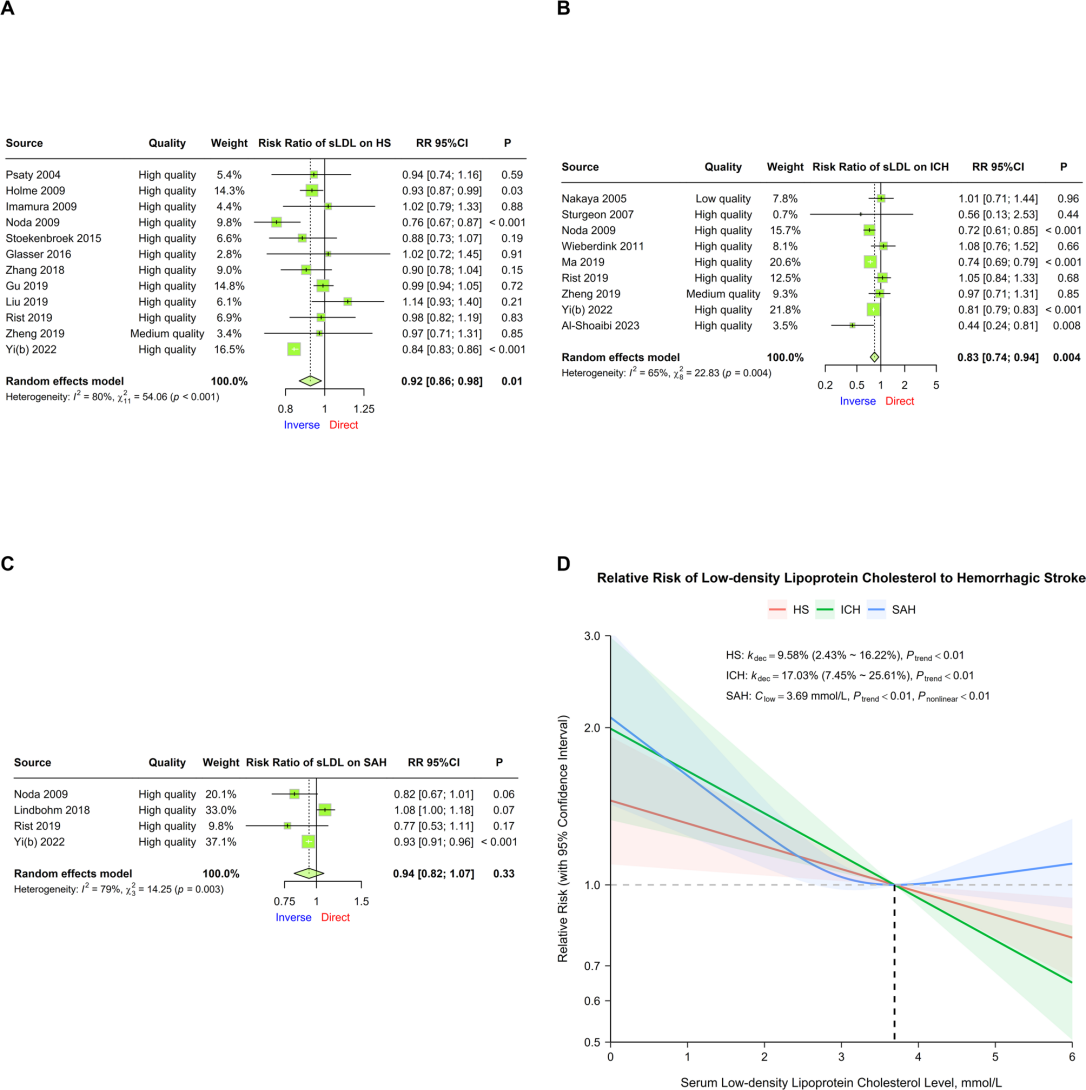
Relationships between serum low-density lipoprotein cholesterol and hemorrhagic stroke risk. **A ~ C** Forest plots of continuous variable analysis of the relative risk of serum low-density Lipoprotein cholesterol on total hemorrhagic stroke, intracerebral hemorrhage, and subarachnoid hemorrhage. Square sizes correspond to cohort weights, horizontal Lines represent 95% confidence intervals, intersection points of crosses represent individual treatment estimates, and diamonds represent pooled treatment estimates. **D** Dose-response analyses between serum low-density Lipoprotein cholesterol and hemorrhagic stroke. Solid Lines represent relative risk trends with changes in serum low-density lipoprotein cholesterol concentration, semitransparent shadings represent corresponding 95% confidence intervals, *k*_dec_ represents the percent of relative risk decreasing per mmol/L increase, *C*_low_ represents the serum low-density lipoprotein cholesterol concentration with the lowest relative risk in a nonlinear relationship, *P*_trend_ represents the *P* value for the trend test in either the linear or nonlinear model, and *P*_nonlinear_ represents the *P* value for Wald’s test in the nonlinear model

Data from 12 cohorts were pooled for CVA on sLDL-C and total HS risk [45, 52–54, 66, 67, 72–74, 76, 77, 85]. An inverse correlation was detected (RR = 0.92, 95% CI 0.86 ~ 0.98, *P* = 0.012), with high heterogeneity (I^2^ = 79.65%, *P* < 0.01; Fig. 3A). Heterogeneity from Yi 2022b was detected, influencing the overall result after omission (RR = 0.94, 95% CI 0.88 ~ 1.00, *P* = 0.06) (Supplemental Fig. 7). Compared with incidence, multivariable regression revealed an increase in inverse correlation with the endpoint of mortality. Subgroup analysis revealed a significant interaction between the state of fasting when serum was collected and the endpoint type, with potential reductions in residual heterogeneity from subgrouping by endpoint type (Table 1).

Data on ICH were obtained from 9 cohorts, revealing an inverse correlation (RR = 0.83, 95% CI 0.74 ~ 0.94, *P* < 0.01) with moderate heterogeneity (I^2^ = 64.96%, *P* < 0.01; Fig. 3B) [47, 50, 54, 59, 75–77, 80, 85]. Subgroup analysis revealed a significant interaction between population source and population size, with potential reductions in residual heterogeneity resulting from subgrouping by proportion of males (Table 1).

Data on SAH were obtained from 4 cohorts, with no significant correlation (RR = 0.94, 95% CI 0.82 ~ 1.07, *P* = 0.329) and high heterogeneity (I^2^ = 78.94%, *P* < 0.01; Fig. 3C) [54, 70, 76, 85].

#### Dose-response analysis

DRA on 9 cohorts revealed significant trends between sLDL-C and total HS risk in both models, whereas the Wald test revealed an insufficient nonlinear trend in the nonlinear model (Model 2, *P* = 0.140). The linear model (Model 1) was the better fit (logLik = 4.729), with a decreasing risk of 9.6% (95% CI 2.4%~16.2%, *P* < 0.01) per mmol/L sLDL-C increase (Fig. 3D; Table 2).

DRA in 9 cohorts revealed significant trends between sLDL-C and ICH risk in both models. A nonlinear trend was demonstrated in the nonlinear model (Model 2, *P* = 0.016), but the linear model (Model 1) was the better fit (logLik = 0.067), with a decreasing risk of 17.0% (95% CI 7.5%~25.6%, *P* < 0.01) per mmol/L sLDL-C increase (Fig. 3D; Table 2).

DRA in 3 cohorts revealed significant trends between sLDL-C and total SAH in both models. A nonlinear trend was demonstrated in the nonlinear model (Model 2, *P* < 0.01), which was also the better fit (logLik = 0.549), with a U-shaped trend of the lowest RR at 3.69 mmol/L, but the increasing risk was not as significant for sLDL-C levels over 3.69 mmol/L (Fig. 3D; Table 2).

### Serum high-density lipoprotein cholesterol

#### Continuous variable analysis

**Fig. 4:**
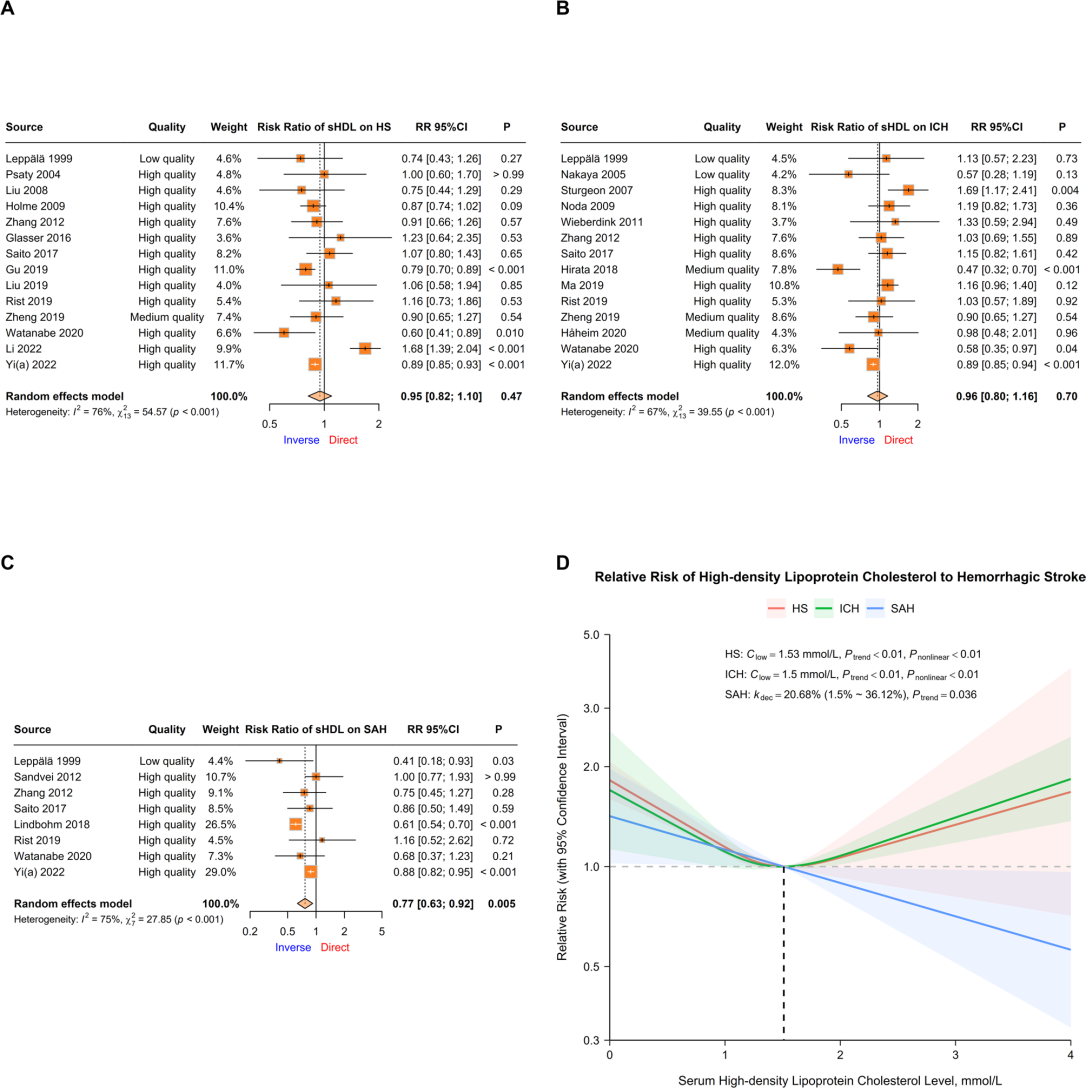
Relationships between serum high-density lipoprotein cholesterol and hemorrhagic stroke risk. **A ~ C** Forest plots of continuous variable analysis of the relative risk of serum high-density Lipoprotein cholesterol on total hemorrhagic stroke, intracerebral hemorrhage, and subarachnoid hemorrhage. Square sizes correspond to cohort weights, horizontal Lines represent 95% confidence intervals, intersection points of crosses represent individual treatment estimates, and diamonds represent pooled treatment estimates. **D** Dose-response analyses between serum high-density Lipoprotein cholesterol and hemorrhagic stroke. Solid Lines represent relative risk trends with changes in serum high-density lipoprotein cholesterol concentration, semitransparent shadings represent corresponding 95% confidence intervals, *k*_dec_ represents the percent of relative risk decreasing per mmol/L increase, *C*_low_ represents the serum high-density lipoprotein cholesterol concentration with the lowest relative risk in a nonlinear relationship, *P*_trend_ represents the *P* value for the trend test in either the linear or nonlinear model, and *P*_nonlinear_ represents the *P* value for Wald’s test in the nonlinear model

Data from 14 cohorts were pooled for CVA on sHDL-C and total HS risk [38, 45, 51, 52, 63, 67, 68, 73, 74, 76, 77, 79, 83, 84]. No significant correlation was detected (RR = 0.95, 95% CI 0.82 ~ 1.10, *P* = 0.466), with high heterogeneity (I^2^ = 76.18%, *P* < 0.01; Fig. 4A). Heterogeneity from Li 2022 was detected, making the overall result significant (RR = 0.87, 95% CI 0.82 ~ 0.93, *P* < 0.01) after omission (Supplemental Fig. 11). Subgroup analysis revealed a significant interaction in the fasting state when serum was collected, although residual heterogeneity persisted across all subgroups (Table 1).

Data on ICH were obtained from 14 cohorts, and no significant correlation was detected (RR = 0.96, 95% CI 0.80 ~ 1.16, *P* = 0.703), with moderate heterogeneity (I^2^ = 67.13%, *P* < 0.01; Fig. 4B) [38, 47, 50, 54, 59, 63, 68, 69, 75–79, 84].

Data on SAH were obtained from 8 cohorts, revealing an inverse correlation (RR = 0.77, 95% CI 0.63 ~ 0.92, *P* < 0.01) with moderate heterogeneity (I^2^ = 74.87%, *P* < 0.01; Fig. 4C) [38, 62, 63, 68, 70, 76, 79, 84]. Multivariable regression revealed an increase in the inverse correlation with longer follow-up duration and attenuation of the inverse correlation with higher quality. Subgroup analysis revealed potential reductions in residual heterogeneity from subgrouping by population source, follow-up duration, and exercise adjustment (Table 1).

#### Dose-response analysis

DRA in 11 cohorts revealed significant trends between sHDL-C and total HS risk only in the nonlinear model (Model 2, *P* < 0.01). The nonlinear trend was confirmed by the Wald test (*P* < 0.01), with a U-shaped trend of the lowest RR at 1.53 mmol/L, but the increasing risk was not as significant for sHDL-C levels over 1.53 mmol/L (Fig. 4D; Table 2).

DRA in 13 cohorts revealed significant trends between sHDL-C and ICH risk only in the nonlinear model (Model 2, *P* < 0.01). The nonlinear trend was confirmed by the Wald test (*P* < 0.01), with a U-shaped trend of the lowest RR at 1.50 mmol/L (Fig. 4D; Table 2).

DRA in 6 cohorts revealed significant trends between sHDL-C and SAH risk in both models. A nonlinear trend was demonstrated in the nonlinear model (Model 2, *P* = 0.015), but the linear model (Model 1) was the better fit (logLik=−2.805), with a decreasing risk of 20.7% (95% CI 1.5%~36.1%, *P* = 0.036) per mmol/L sHDL-C increase (Fig. 4D; Table 2).

### Serum triglycerides

#### Continuous variable analysis

**Fig. 5:**
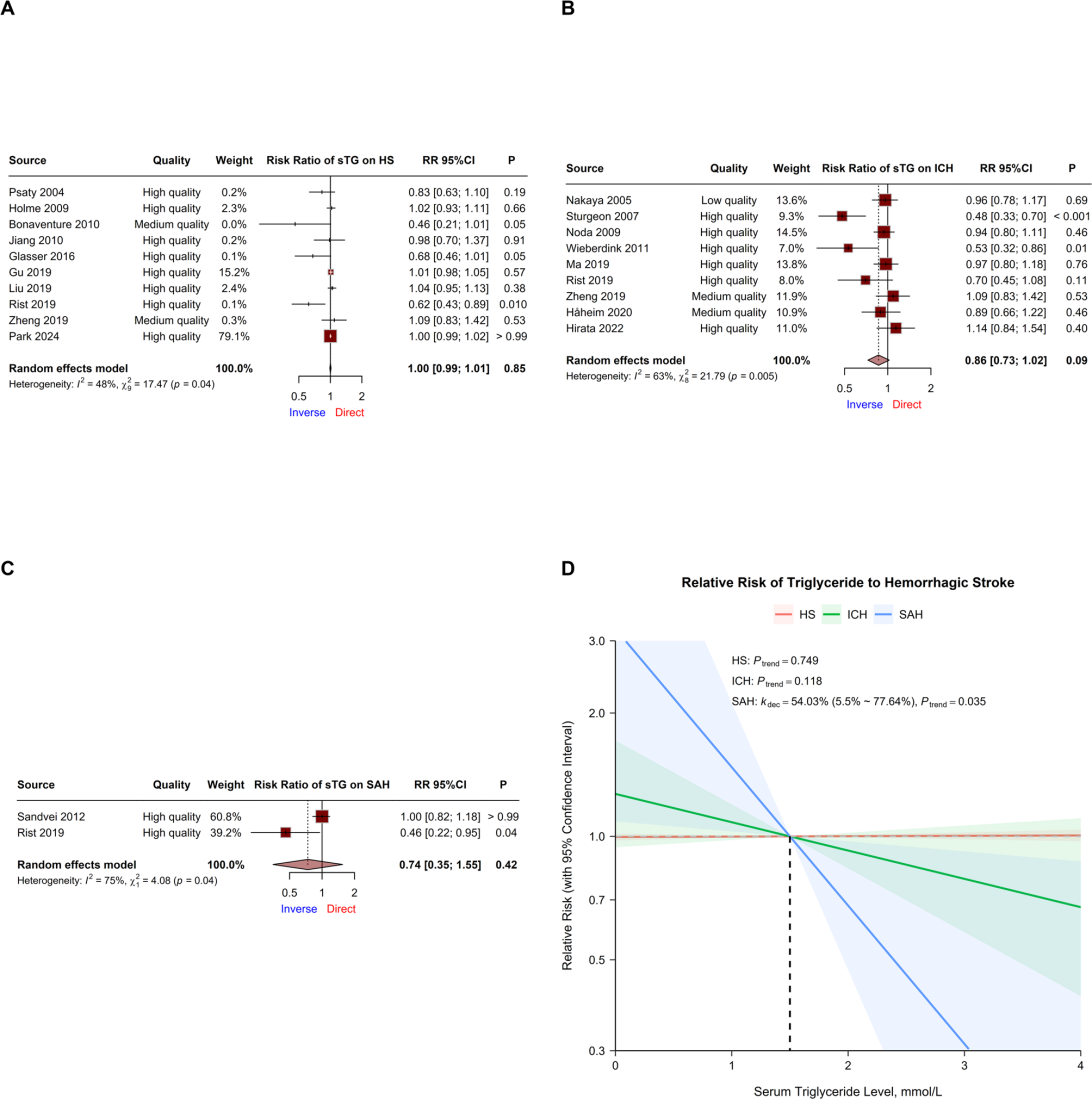
Relationships between serum triglyceride and hemorrhagic stroke risk. **A ~ C** Forest plots of continuous variable analysis of the relative risk of serum triglyceride on total hemorrhagic stroke, intracerebral hemorrhage, and subarachnoid hemorrhage. Square sizes correspond to cohort weights, horizontal Lines represent 95% confidence intervals, intersection points of crosses represent individual treatment estimates, and diamonds represent pooled treatment estimates. **D** Dose-response analyses between serum triglyceride and hemorrhagic stroke. Solid Lines represent relative risk trends with changes in serum triglyceride concentration, semitransparent shadings represent corresponding 95% confidence intervals, *k*_dec_ represents the percent of relative risk decreasing per mmol/L increase, *C*_low_ represents the serum triglyceride concentration with the lowest relative risk in a nonlinear relationship, *P*_trend_ represents the *P* value for the trend test in either the linear or nonlinear model, and *P*_nonlinear_ represents the *P* value for Wald’s test in a nonlinear model

Data from 10 cohorts were pooled for CVA on sTG and total HS risk [45, 52, 55, 56, 67, 73, 74, 76, 77, 86]. No significant correlation was detected (RR = 1.00, 95% CI 0.99 ~ 1.01, *P* = 0.847), with low heterogeneity (I^2^ = 48.50%, *P* = 0.042; Fig. 5A). Subgroup analysis revealed significant interactions between population source and population age and potential reductions in residual heterogeneity from subgrouping by population age, alcohol abuse adjustment, BMI adjustment, and antihypertensive drug adjustment (Table 1).

Data on ICH were obtained from 9 cohorts, and no significant correlation was detected (RR = 0.86, 95% CI 0.73 ~ 1.02, *P* = 0.091), with moderate heterogeneity (I^2^ = 63.28%, *P* < 0.01; Fig. 5B) [47, 50, 54, 59, 75–78, 82]. Multivariable regression revealed direct effects in the Asian population compared with the American population. Subgroup analysis also revealed a significant interaction in population source, with corresponding subgrouping potentially reducing residual heterogeneity (Table 1).

Data on SAH were obtained from 2 cohorts, with no significant correlation (RR = 0.74, 95% CI 0.35 ~ 1.55, *P* = 0.422) with high heterogeneity (I^2^ = 75.48%, *P* = 0.043; Fig. 5C) [62, 76].

#### Dose-response analysis

DRA in 7 cohorts revealed no significant trend between sTG and total HS risk, and DRA in 8 cohorts revealed no significant trend between sTG and ICH risk.

DRA in 1 cohort revealed a significant trend between sTG and SAH risk only in the linear model (Model 1, *P* = 0.035), with a decreasing risk of 54.0% (95% CI 5.5%~77.6%, *P* = 0.035) per mmol/L sTG increase (Fig. 5D; Table 2).

## Discussion

This meta-analysis employed CVA and DRA to investigate the relationship between serum Lipids and HS risk in global community-based populations, with potential nonlinear trends validated through model comparisons. Although a nonlinear trend between sTC and ICH was detected with the lowest RR at 6 mmol/L, the Linear model demonstrated superior goodness-of-fit between sTC and overall HS. sLDL-C presented a negative Linear trend with both overall HS and ICH, whereas sHDL-C presented subtype-specific effects, with the lowest HS risk at 1.53 mmol/L.

Adopting individual lipid management targets under corresponding ASCVD risk estimation is essential, with sLDL-C regarded as the primary target for secondary prevention of ASCVD [8, 87, 88]. Current guidelines recommend an sLDL-C reduction of > 50% from baseline or achieving sLDL-C levels < 1.4 mmol/L for ultrahigh risk, a > 50% reduction or < 1.8 mmol/L for very high risk, and levels < 2.6 mmol/L for medium and high risk, with no lower threshold for sLDL-C established where clinical benefits ceased or adverse effects emerged [87, 88]. While clinical evidence has reinforced the “lowest is best” in sLDL-C management of populations with defined ASCVD risks and refuted concerns about safety-driven undertreatment [8, 89, 90], this practice statement should be framed as reassurance against withholding intensive treatment when clinically indicated rather than advocating for untargeted aggressive lipid lowering in unselected populations.

Concerns about ASCVD have led to premature or intensive lipid-lowering interventions in community-based populations, yet potential HS risk remains a major consideration. The Joint Committee on the Chinese Guidelines for Lipid Management proposed sTC < 5.2 mmol/L, sLDL-C < 3.4 mmol/L, sTG < 1.7 mmol/L, and serum non-HDL-C < 4.1 mmol/L as reasonable levels for primary prevention in populations with low ASCVD risk, but the corresponding lower limits or specific targets for sHDL-C management remain inconclusive. However, emerging evidence suggests that hypocholesterolemia compromises vascular endothelial function and integrity, whereas uncontrolled hypertension further exacerbates microaneurysm formation in individuals with low sLDL-C levels, thus increasing HS risk [72]. Prolonged follow-up suggested increased HS risk at reduced sLDL-C levels in community-based populations, with significantly decreased risks at sLDL-C < 2.5 mmol/L for SAH and < 3.5 mmol/L for ICH [75, 76, 85], necessitating the cautious use of lipid-lowering agents in individuals with minimal or no ASCVD risk. Here, the minimal important difference (MID) was introduced to define clinically meaningful serum Lipid ranges, which were set at an RR ranging form 0.87 ~ 1.15 on the basis of GRADE recommendations and prior clinical practice [91]. This study suggested that both low sTC (< 3.48 mmol/L) and low sLDL-C (< 1.09 mmol/L) were independent risk factors for HS, with these thresholds representing the upper MID limits (RR = 1.15) relative to the HS risk at conventionally recommended targets (sTC 5.20 mmol/L and sLDL-C 3.40 mmol/L, respectively). Consequently, ranges of 3.48 ~ 5.20 mmol/L for sTC and 1.09–3.40 mmol/L for sLDL-C might better balance ASCVD prevention with HS risk in community-based populations.

Although current guidelines only consider sHDL-C < 1.0 mmol/L as the standard for reduction, supra-optimal sHDL-C levels might increase HS risk [68, 82]. Clinical trials on cholesteryl ester transfer protein (CETP) inhibitors revealed elevated sHDL-C but unexpected increases in cardiovascular mortality [92], potentially attributable to cholesterol-overloaded high-density lipoprotein particles (HDL-P), compensation with sHDL-C reverse transport dysfunction, and endothelial dysfunction [93]. This study revealed the lowest risk of both HS and ICH at an sHDL-C level of approximately 1.5 mmol/L, and when the MID was set at an RR of 0.87 ~ 1.15, a clinical sHDL-C range of 0.99 ~ 2.34 mmol/L might provide maximal clinical benefit while minimizing potential risks in community-based populations.

Notably, a novel U-shaped trend was detected between sLDL-C and SAH risk, although its correlation with high sLDL-C levels was not specified. In several cohorts, high sLDL-C levels increased SAH risk predominantly in males, potentially related to the vascular protective effect of estrogen [63, 70], suggesting that sex is a confounding factor.

Age represents a critical determinant in the adoption of individualized lipid Management targets, especially in SAH prevention. In this study, subgroup analyses revealed age-dependent heterogeneity between sTC and SAH risk, with a direct correlation in younger individuals but an inverse correlation in elder individuals, indicating increased hemorrhagic risk in elderly individuals. A nationwide study of 15.6 million Koreans revealed an age-dependent relationship, where hypertriglyceridemia increased HS risk in young-to-middle-aged populations but had protective effects on elderly individuals [86]. In alignment with the stratified risk patterns, the European Society of Cardiology (ESC) and the European Atherosclerosis Society (EAS) restricted statin therapy in individuals aged 75 years or more to those with high ASCVD risk and recommended initiation at low doses when necessary to minimize adverse events [88].

The individual lipid management targets suggested a comprehensive approach extending beyond pharmacotherapy alone. The Joint Committee on the Chinese Guidelines for Lipid Management has emphasized healthy lifestyles as foundational to lipid management, advocating against premature or intensive pharmacological intervention in community-based populations without definite ASCVD risk, as more individuals might benefit from lifestyle modifications, such as physical activity and dietary optimization [87]. Critically, this study suggested that low serum lipid levels might increase HS risk, necessitating regular monitoring. In accordance with ESC/EAS recommendations, clinicians should adopt a shared decision-making (SDM) framework for ASCVD risk Management, incorporating Lipid assessment every 8 weeks after initiation, as well as attention to other ASCVD risk factors, such as hypertension, to further optimize the balance between therapeutic efficacy and safety [88].

Although once required to be sampled in the fasting state, current guidelines suggest little difference between lipid profiles from fasting and nonfasting serum samples [88]. Given the better patient acceptability, nonfasting sampling might be preferable for lipid examination, except in populations requiring strict metabolic monitoring, such as those with diabetes mellitus or hypertriglyceridemia. Interestingly, this study suggested that sTC levels from random serum samples might better predict ICH risk than those from fasting samples, potentially because of pioneer damage to lipid tolerance.

### Study strengths and limitations

Our meta-analysis had several strengths. First, by focusing on ostensibly healthy populations rather than individuals with definite ASCVD risk, this study provided generalizable insights that are applicable to community-based populations. Second, a dual analysis, which combined random effects analysis for linear correlation synthesis and DRA for nonlinear trend detection, ensured comprehensive data utilization while preserving potential nonlinear relationships. Third, stratified analyses of HS subtypes revealed critical distinctions, where sHDL-C demonstrated linear protective effects against SAH, which used to be covered by the predominance of ICH, suggesting prevention strategies specific to HS subtypes. Finally, model selection was enhanced through likelihood comparisons, with the linear model demonstrating superior goodness-of-fit over the previous U-shaped trend between sTC and total HS [15–17], particularly due to the exclusion of studies on ICH only, whose U-shaped trend was confirmed, with the lowest risk at 6.07 mmol/L.

However, this study also had several limitations warrant consideration. First, the analysis of observational cohorts led to limitations in causal inference. While predefined methodological criteria were applied during data extraction, heterogeneity in how original studies measured or adjusted for confounders remained unavoidable, which was compounded by the inability to account for confounder variations during the follow-up. Although subgroup analyses and meta-regression were performed, certain limitations persisted. For example, mortality data proved inadequate for burden assessment given the improvement in acute care over time. While the protocol excluded populations with baseline conditions that might have affected serum lipid levels during study selection and downgraded studies on populations with hypertension, obesity, tobacco dependence, and hyperlipidemia during quality assessment, the reliance on occasional or scheduled health examinations potentially introduced selection bias by oversampling individuals with ASCVD concerns, leading to medical consultations. Second, although evidence-based subgroups and cutoff points were chosen to maximize the clinical rationale and subgroups containing only one study were excluded to avoid occasional influence, the results of subgroup analysis should be interpreted with caution. The absence of individual-level data led to reduced sensitivity of subgroup categorization and imbalances in group sizes, which might obscure real effects. Third, few types of serum lipids were analyzed. Although increasing evidence suggests that lipid ratios, such as sLDL-C/sHDL-C and sTG/sHDL-C, might serve as superior markers for lipid metabolism and vascular risk stratification, research in this area remains relatively insufficient, and their clinical applicability still poses challenges [94, 95]. Finally, despite noted sex differences in serum lipid effects [35, 68, 74], sex-specific analysis was not conducted because of insufficient samples; instead, subgroup analysis and meta-regression on the male proportion were conducted because of the potential influence of sex.

In view of these limitations, future research should prioritize large and standardized cohorts with open-access data on the variation in risk factors and accompanying treatment to improve causal inference. Stratified analyses across different ASCVD risk populations are also needed to refine serum lipid ranges for individual lipid management. Novel lipid biomarkers, such as serum non-HDL-C and sLDL-C/sHDL-C, should also be reported to establish their clinical application. Collaborative data programs could overcome current sample limitations, enabling more precise risk assessment and individualized management strategies that balance ischemic and hemorrhagic risks.

## Conclusion

This study revealed that sTC and sLDL-C presented a negative Linear relationship with HS, and sHDL-C presented a U-shaped relationship with HS, with a minimum risk of 1.53 mmol/L in global community-based populations. When considering an MID of RR 0.87 ~ 1.15, Maintaining sTC between 3.48 ~ 5.20 mmol/L, sLDL-C between 1.09 ~ 3.40 mmol/L, and sHDL-C between 0.99 ~ 2.34 mmol/L might provide an optimal balance for lipid management, contributing to stroke prevention in global community-based populations. Although the safety range was rather wide, this study cautioned against indiscriminate lipid-lowering therapy, as excessively premature or intensive lipid-lowering therapy reduction might increase hemorrhagic risk without cardiovascular benefit. Individualized management based on ASCVD risk, combined with regular monitoring and patient compliance, could further optimize stroke prevention while minimizing unintended hemorrhagic harm.

## Supplementary Information

Supplementary Material 1.

## Data Availability

Analysis codes are available from the corresponding authors upon reasonable request.

## Abbreviations

95% CI: 95% confidence interval
ASCVD: Atherosclerotic cardiovascular disease
CETP: Cholesteryl ester transfer protein
CVA: Continuous variable analysis
DRA: Dose-response analysis
EAS: European Atherosclerosis Society
ESC: European Society of Cardiology
GBD: Global Burden of Diseases
GDT: Guideline Development Tool
GRADE: Grading of Recommendations Assessment, Development and Evaluation
HDL-P: High-density lipoprotein particles
HR: Hazard ratio
HS: Hemorrhagic stroke
ICD: International Classification of Diseases
ICH: Intracerebral hemorrhage
IS: Ischemic stroke
Loglik: Log-likelihood
MID: Minimal important difference
non-HDL-C: Non-high-density lipoprotein cholesterol
NOS: Newcastle-Ottawa Scale
OR: Odds ratio
REM: Random-effects model
RR: Relative risk
SAH: Subarachnoid hemorrhage
SDM: Shared decision-making
sHDL-C: Serum high-density lipoprotein cholesterol
sLDL-C: Serum low-density lipoprotein cholesterol
sTC: Serum total cholesterol
sTG: Serum triglyceride
