# Supplementary material for: Relationship between serum lipids and the risk of hemorrhagic stroke: a meta-analysis of 50 million participants from prospective cohort studies
Source: Lipids Health Dis. 2025 Sep 30;24:303. doi: 10.1186/s12944-025-02698-0 (PMC12487445; doi:10.1186/s12944-025-02698-0)

# Title

Relationship between Serum Lipids and the Risk of Hemorrhagic Stroke: A Meta-Analysis of 50 Million Participants from Prospective Cohort Studies

# Supplement Material

Contents

[**Supplemental Table 1** Preferred Reporting Items for Systematic reviews and Meta-Analyses 2020 checklist. 8](#_Toc205670686)

[**Supplemental Table 2** Detailed stress for searching. 10](#_Toc205670687)

[**Supplemental Table 3** Characteristics of studies included. 12](#_Toc205670688)

[**Supplemental Table 4** Independent and final results in quality assessment. 17](#_Toc205670689)

[**Supplemental Table 5** Newcastle-Ottawa Scale for Prospective Cohort Studies. 19](#_Toc205670690)

[**Supplemental Table 6** Summary of univariate meta-regression analysis for continuous variable analysis on relationship between serum lipids and risk of hemorrhagic stroke. 21](#_Toc205670691)

[**Supplemental Table 7** Summary of multivariate meta-regression analysis for continuous variable analysis on relationship between serum lipids and risk of hemorrhagic stroke. 24](#_Toc205670692)

[**Supplemental Table 8** Summary of dose-response relationship between serum lipids and hemorrhagic stroke. 26](#_Toc205670693)

[**Supplemental Table 9** Certainty assessment of analyses for relationship between serum lipids and risk of hemorrhagic stroke. 27](#_Toc205670694)

[**Supplemental Figure 1** Continuous variable analysis on relationship between serum total cholesterol and risk of total hemorrhagic stroke; **A)** Baujat plot for heterogeneity analysis, **B)** Funnel plot for bias on publication, **C)** Funnel plot for bias on publication after trim-and-fill method, **D)** Bubble plot for meta-regression on publish years, **E)** Forest plot for risk ratio after trim-and-fill method, **F)** Forest plot for risk ratio after one-by-one exclusion. 29](#_Toc205670695)

[**Supplemental Figure 2** Forest plots of subgroup analysis of continuous variable analysis on risk ratio of serum total cholesterol on total hemorrhagic stroke. p_β_ represents *P* value for treatment estimates within subgroups; p_i_ represents *P* value for heterogeneity within subgroups; p_e_ as represents *P* value for heterogeneity between subgroups. 30](#_Toc205670696)

[**Supplemental Figure 3** Continuous variable analysis on relationship between serum total cholesterol and risk of intracerebral hemorrhage; **A)** Baujat plot for heterogeneity analysis, **B)** Funnel plot for bias on publication, **C)** Funnel plot for bias on publication after trim-and-fill method, **D)** Bubble plot for meta-regression on publish years, **E)** Forest plot for risk ratio after trim-and-fill method, **F)** Forest plot for risk ratio after one-by-one exclusion. 31](#_Toc205670697)

[**Supplemental Figure 4** Forest plots of subgroup analysis of continuous variable analysis on risk ratio of serum total cholesterol on intracerebral hemorrhage. p_β_ represents *P* value for treatment estimates within subgroups; p_i_ represents *P* value for heterogeneity within subgroups; p_e_ as represents *P* value for heterogeneity between subgroups. 32](#_Toc205670698)

[**Supplemental Figure 5** Continuous variable analysis on relationship between serum total cholesterol and risk of subarachnoid hemorrhage; **A)** Baujat plot for heterogeneity analysis, **B)** Funnel plot for bias on publication, **C)** Funnel plot for bias on publication after trim-and-fill method, **D)** Bubble plot for meta-regression on publish years, **E)** Forest plot for risk ratio after trim-and-fill method, **F)** Forest plot for risk ratio after one-by-one exclusion. 33](#_Toc205670699)

[**Supplemental Figure 6** Forest plots of subgroup analysis of continuous variable analysis on risk ratio of serum total cholesterol on subarachnoid hemorrhage. p_β_ represents *P* value for treatment estimates within subgroups; p_i_ represents *P* value for heterogeneity within subgroups; p_e_ as represents *P* value for heterogeneity between subgroups. 34](#_Toc205670700)

[**Supplemental Figure 7** Continuous variable analysis on relationship between serum low-density lipoprotein cholesterol and risk of total hemorrhagic stroke; **A)** Baujat plot for heterogeneity analysis, **B)** Funnel plot for bias on publication, **C)** Funnel plot for bias on publication after trim-and-fill method, **D)** Bubble plot for meta-regression on publish years, **E)** Forest plot for risk ratio after trim-and-fill method, **F)** Forest plot for risk ratio after one-by-one exclusion. 35](#_Toc205670701)

[**Supplemental Figure 8** Forest plots of subgroup analysis of continuous variable analysis on risk ratio of serum low-density lipoprotein cholesterol on total hemorrhagic stroke. p_β_ represents *P* value for treatment estimates within subgroups; p_i_ represents *P* value for heterogeneity within subgroups; p_e_ as represents *P* value for heterogeneity between subgroups. 36](#_Toc205670702)

[**Supplemental Figure 9** Continuous variable analysis on relationship between serum low-density lipoprotein cholesterol and risk of intracerebral hemorrhage; **A)** Baujat plot for heterogeneity analysis, **B)** Funnel plot for bias on publication, **C)** Funnel plot for bias on publication after trim-and-fill method, **D)** Bubble plot for meta-regression on publish years, **E)** Forest plot for risk ratio after trim-and-fill method, **F)** Forest plot for risk ratio after one-by-one exclusion. 37](#_Toc205670703)

[**Supplemental Figure 10** Forest plots of subgroup analysis of continuous variable analysis on risk ratio of serum low-density lipoprotein cholesterol on intracerebral hemorrhage. p_β_ represents *P* value for treatment estimates within subgroups; p_i_ represents *P* value for heterogeneity within subgroups; p_e_ as represents *P* value for heterogeneity between subgroups. 38](#_Toc205670704)

[**Supplemental Figure 11** Continuous variable analysis on relationship between serum high-density lipoprotein cholesterol and risk of total hemorrhage stroke; **A)** Baujat plot for heterogeneity analysis, **B)** Funnel plot for bias on publication, **C)** Funnel plot for bias on publication after trim-and-fill method, **D)** Bubble plot for meta-regression on publish years, **E)** Forest plot for risk ratio after trim-and-fill method, **F)** Forest plot for risk ratio after one-by-one exclusion. 39](#_Toc205670705)

[**Supplemental Figure 12** Forest plots of subgroup analysis of continuous variable analysis on risk ratio of serum high-density lipoprotein cholesterol on total hemorrhagic stroke. p_β_ represents *P* value for treatment estimates within subgroups; p_i_ represents *P* value for heterogeneity within subgroups; p_e_ as represents *P* value for heterogeneity between subgroups. 40](#_Toc205670706)

[**Supplemental Figure 13** Continuous variable analysis on relationship between serum high-density lipoprotein cholesterol and risk of intracerebral hemorrhage; **A)** Baujat plot for heterogeneity analysis, **B)** Funnel plot for bias on publication, **C)** Funnel plot for bias on publication after trim-and-fill method, **D)** Bubble plot for meta-regression on publish years, **E)** Forest plot for risk ratio after trim-and-fill method, **F)** Forest plot for risk ratio after one-by-one exclusion. 41](#_Toc205670707)

[**Supplemental Figure 14** Forest plots of subgroup analysis of continuous variable analysis on risk ratio of serum high-density lipoprotein cholesterol on intracerebral hemorrhage. p_β_ represents *P* value for treatment estimates within subgroups; p_i_ represents *P* value for heterogeneity within subgroups; p_e_ as represents *P* value for heterogeneity between subgroups. 42](#_Toc205670708)

[**Supplemental Figure 15** Continuous variable analysis on relationship between serum high-density lipoprotein cholesterol and risk of subarachnoid hemorrhage; **A)** Baujat plot for heterogeneity analysis, **B)** Funnel plot for bias on publication, **C)** Funnel plot for bias on publication after trim-and-fill method, **D)** Bubble plot for meta-regression on publish years, **E)** Forest plot for risk ratio after trim-and-fill method, **F)** Forest plot for risk ratio after one-by-one exclusion. 43](#_Toc205670709)

[**Supplemental Figure 16** Forest plots of subgroup analysis of continuous variable analysis on risk ratio of serum high-density lipoprotein cholesterol on subarachnoid hemorrhage. p_β_ represents *P* value for treatment estimates within subgroups; p_i_ represents *P* value for heterogeneity within subgroups; p_e_ as represents *P* value for heterogeneity between subgroups. 44](#_Toc205670710)

[**Supplemental Figure 17** Continuous variable analysis on relationship between serum triglyceride and risk of total hemorrhage stroke; **A)** Baujat plot for heterogeneity analysis, **B)** Funnel plot for bias on publication, **C)** Funnel plot for bias on publication after trim-and-fill method, **D)** Bubble plot for meta-regression on publish years, **E)** Forest plot for risk ratio after trim-and-fill method, **F)** Forest plot for risk ratio after one-by-one exclusion. 45](#_Toc205670711)

[**Supplemental Figure 18** Forest plots of subgroup analysis of continuous variable analysis on risk ratio of serum triglyceride on total hemorrhagic stroke. p_β_ represents *P* value for treatment estimates within subgroups; p_i_ represents *P* value for heterogeneity within subgroups; p_e_ as represents *P* value for heterogeneity between subgroups. 46](#_Toc205670712)

[**Supplemental Figure 19** Continuous variable analysis on relationship between serum triglyceride and risk of intracerebral hemorrhage; **A)** Baujat plot for heterogeneity analysis, **B)** Funnel plot for bias on publication, **C)** Funnel plot for bias on publication after trim-and-fill method, **D)** Bubble plot for meta-regression on publish years, **E)** Forest plot for risk ratio after trim-and-fill method, **F)** Forest plot for risk ratio after one-by-one exclusion. 47](#_Toc205670713)

[**Supplemental Figure 20** Forest plots of subgroup analysis of continuous variable analysis on risk ratio of serum triglyceride on intracerebral hemorrhage. p_β_ represents *P* value for treatment estimates within subgroups; p_i_ represents *P* value for heterogeneity within subgroups; p_e_ as represents *P* value for heterogeneity between subgroups. 48](#_Toc205670714)

**Supplemental Table 1** Preferred Reporting Items for Systematic reviews and Meta-Analyses 2020 checklist.

| **Section and Topic** | **Item #** | **Checklist item** | **Location where item is reported** |
| --- | --- | --- | --- |
| **TITLE** | | |  |
| Title | 1 | Identify the report as a systematic review. | Page 1 |
| **ABSTRACT** | | |  |
| Abstract | 2 | See the PRISMA 2020 for Abstracts checklist. | Page 2 |
| **INTRODUCTION** | | |  |
| Rationale | 3 | Describe the rationale for the review in the context of existing knowledge. | Page 4 |
| Objectives | 4 | Provide an explicit statement of the objective(s) or question(s) the review addresses. | Page 5 |
| **METHODS** | | |  |
| Eligibility criteria | 5 | Specify the inclusion and exclusion criteria for the review and how studies were grouped for the syntheses. | Page 5 |
| Information sources | 6 | Specify all databases, registers, websites, organisations, reference lists and other sources searched or consulted to identify studies. Specify the date when each source was last searched or consulted. | Page 6 |
| Search strategy | 7 | Present the full search strategies for all databases, registers and websites, including any filters and limits used. | Page 7, Supplemental Table 2 |
| Selection process | 8 | Specify the methods used to decide whether a study met the inclusion criteria of the review, including how many reviewers screened each record and each report retrieved, whether they worked independently, and if applicable, details of automation tools used in the process. | Page 7 |
| Data collection process | 9 | Specify the methods used to collect data from reports, including how many reviewers collected data from each report, whether they worked independently, any processes for obtaining or confirming data from study investigators, and if applicable, details of automation tools used in the process. | Page 7 |
| Data items | 10a | List and define all outcomes for which data were sought. Specify whether all results that were compatible with each outcome domain in each study were sought (e.g. for all measures, time points, analyses), and if not, the methods used to decide which results to collect. | Page 7 |
|  | 10b | List and define all other variables for which data were sought (e.g. participant and intervention characteristics, funding sources). Describe any assumptions made about any missing or unclear information. | Page 7 |
| Study risk of bias assessment | 11 | Specify the methods used to assess risk of bias in the included studies, including details of the tool(s) used, how many reviewers assessed each study and whether they worked independently, and if applicable, details of automation tools used in the process. | Page 8 |
| Effect measures | 12 | Specify for each outcome the effect measure(s) (e.g. risk ratio, mean difference) used in the synthesis or presentation of results. | Page 6 |
| Synthesis methods | 13a | Describe the processes used to decide which studies were eligible for each synthesis (e.g. tabulating the study intervention characteristics and comparing against the planned groups for each synthesis (item #5)). | Page 8 |
|  | 13b | Describe any methods required to prepare the data for presentation or synthesis, such as handling of missing summary statistics, or data conversions. | Page 8 |
|  | 13c | Describe any methods used to tabulate or visually display results of individual studies and syntheses. | Page 8 |
|  | 13d | Describe any methods used to synthesize results and provide a rationale for the choice(s). If meta-analysis was performed, describe the model(s), method(s) to identify the presence and extent of statistical heterogeneity, and software package(s) used. | Page 8 |
|  | 13e | Describe any methods used to explore possible causes of heterogeneity among study results (e.g. subgroup analysis, meta-regression). | Page 9 |
|  | 13f | Describe any sensitivity analyses conducted to assess robustness of the synthesized results. | Page 9 |
| Reporting bias assessment | 14 | Describe any methods used to assess risk of bias due to missing results in a synthesis (arising from reporting biases). | Page 9 |
| Certainty assessment | 15 | Describe any methods used to assess certainty (or confidence) in the body of evidence for an outcome. | Page 9 |
| **RESULTS** | | |  |
| Study selection | 16a | Describe the results of the search and selection process, from the number of records identified in the search to the number of studies included in the review, ideally using a flow diagram. | Figure 1 |
|  | 16b | Cite studies that might appear to meet the inclusion criteria, but which were excluded, and explain why they were excluded. | Page 11 |
| Study characteristics | 17 | Cite each included study and present its characteristics. | Page 11 |
| Risk of bias in studies | 18 | Present assessments of risk of bias for each included study. | Page 11 |
| Results of individual studies | 19 | For all outcomes, present, for each study: (a) summary statistics for each group (where appropriate) and (b) an effect estimate and its precision (e.g. confidence/credible interval), ideally using structured tables or plots. | Supplemental Table 3 |
| Results of syntheses | 20a | For each synthesis, briefly summarise the characteristics and risk of bias among contributing studies. | Page 11 |
|  | 20b | Present results of all statistical syntheses conducted. If meta-analysis was done, present for each the summary estimate and its precision (e.g. confidence/credible interval) and measures of statistical heterogeneity. If comparing groups, describe the direction of the effect. | Page 11 |
|  | 20c | Present results of all investigations of possible causes of heterogeneity among study results. | Page 11 |
|  | 20d | Present results of all sensitivity analyses conducted to assess the robustness of the synthesized results. | Page 11 |
| Reporting biases | 21 | Present assessments of risk of bias due to missing results (arising from reporting biases) for each synthesis assessed. | Page 11 |
| Certainty of evidence | 22 | Present assessments of certainty (or confidence) in the body of evidence for each outcome assessed. | Supplemental Table 9 |
| **DISCUSSION** | | |  |
| Discussion | 23a | Provide a general interpretation of the results in the context of other evidence. | Page 18 |
|  | 23b | Discuss any limitations of the evidence included in the review. | Page 22 |
|  | 23c | Discuss any limitations of the review processes used. | Page 23 |
|  | 23d | Discuss implications of the results for practice, policy, and future research. | Page 24 |
| **OTHER INFORMATION** | | |  |
| Registration and protocol | 24a | Provide registration information for the review, including register name and registration number, or state that the review was not registered. | Page 5 |
|  | 24b | Indicate where the review protocol can be accessed, or state that a protocol was not prepared. | Page 5 |
|  | 24c | Describe and explain any amendments to information provided at registration or in the protocol. | Page 5 |
| Support | 25 | Describe sources of financial or non-financial support for the review, and the role of the funders or sponsors in the review. | Page 26 |
| Competing interests | 26 | Declare any competing interests of review authors. | Page 26 |
| Availability of data, code and other materials | 27 | Report which of the following are publicly available and where they can be found: template data collection forms; data extracted from included studies; data used for all analyses; analytic code; any other materials used in the review. | Page 26 |

**Supplemental Table 2** Detailed stress for searching.

**Database: Ovid MEDLINE(R) ALL <1946 to May 20, 2025>**

| **No.** | **Searches** | **Num.** |
| --- | --- | --- |
| 1 | exp intracranial hemorrhages/ or exp hemorrhagic stroke/ | 85,668 |
| 2 | (intracranial h?emorrhag* or cerebral h?emorrhag* or subarachnoid h?emorrhag* or h?emorrhagic stroke?).ab,ti,kw. | 68,891 |
| 3 | 1 or 2 | 117,940 |
| 4 | exp Lipid Metabolism Disorders/ or exp Cholesterol/ or exp Triglycerides/ | 314,790 |
| 5 | (cholesterol or triglyceride or LDL or HDL).ab,ti,kw. | 371,750 |
| 6 | 4 or 5 | 506,846 |
| 7 | exp cohort studies/ or exp observational study/ or exp risk assessment/ or exp risk factors/ | 3,649,099 |
| 8 | (prospective stud* or follow-up stud* or cohort stud* or observational stud* or longitudinal stud*).ab,ti,kw. | 969,551 |
| 9 | 7 or 8 | 3,949,542 |
| 10 | (journal article or observational study).pt. | 36,348,955 |
| 11 | (retrospective stud* or case report? or case-control stud* or randomized controlled trial? or meta analys#s or systematic review? or review?).ti,kw. | 1,477,618 |
| 12 | (3 and 6 and 9 and 10) not 11 | 764 |

**Cochrane Library Date Run: 21/05/2025 16:38:51**

| **ID** | **Search** | **Hits** |
| --- | --- | --- |
| #1 | MeSH descriptor: [Intracranial Hemorrhages] explode all trees | 3,233 |
| #2 | MeSH descriptor: [Hemorrhagic Stroke] explode all trees | 88 |
| #3 | (intracranial h?emorrhag* or cerebral h?emorrhag* or subarachnoid h?emorrhag* or h?emorrhagic stroke?):ti,ab,kw | 12,603 |
| #4 | #1 or #2 or #3 | 12,827 |
| #5 | MeSH descriptor: [Lipid Metabolism Disorders] explode all trees | 10,238 |
| #6 | MeSH descriptor: [Cholesterol] explode all trees | 12,793 |
| #7 | MeSH descriptor: [Triglycerides] explode all trees | 7,855 |
| #8 | (cholesterol or triglyceride or LDL or HDL):ti,ab,kw | 54,085 |
| #9 | #5 or #6 or #7 or #8 | 57,996 |
| #10 | #4 and #9 | 281 |
| #11 | #10 in Trials | 275 |

**EMBASE from 1947 to 21 May 2025**

| No. | Query | Results |
| --- | --- | --- |
| #12 | #3 AND #6 AND #9 AND #10 NOT #11 | 2,580 |
| #11 | 'retrospective stud*' OR 'case report$' OR 'case-control stud*' OR 'randomized controlled trial$' OR 'meta-analys?s' OR 'systematic review$' OR 'review$':ti,kw | 7,671,039 |
| #10 | 'article'/it | 31,302,403 |
| #9 | #7 OR #8 | 6,068,381 |
| #8 | 'prospective stud*' OR 'follow-up stud*' OR 'cohort stud*' OR 'observational stud*' OR 'longitudinal stud*':ab,ti,kw | 2,102,621 |
| #7 | 'prospective study'/de OR 'follow up'/de OR 'cohort analysis'/de OR 'observational study'/de OR 'risk factor'/de OR 'risk assessment'/de | 5,784,180 |
| #6 | #4 OR #5 | 1,080,217 |
| #5 | cholesterol OR triglyceride OR ldl OR hdl:ab,ti,kw | 681,940 |
| #4 | 'lipid metabolism disorders'/exp OR 'cholesterol'/exp OR 'triglycerides'/exp | 924,285 |
| #3 | #1 OR #2 | 221,871 |
| #2 | 'intracranial h$emorrhag*' OR 'cerebral h$emorrhag*' OR 'subarachnoid h$emorrhag*' OR 'h$emorrhagic stroke$':ab,ti,kw | 122,293 |
| #1 | 'intracranial hemorrhages'/exp OR 'hemorrhagic stroke'/exp | 206,910 |

**Supplemental Table 3** Characteristics of studies included.

| **Study** | **Population** | **Number** | **Follow-up (years)** | **Sexual (male, %)** | **Age (mean or average)** | **Confounding variables** | **Effect size** | **Exposure** | **Fasting state** | **Endpoint (Number)** |
| --- | --- | --- | --- | --- | --- | --- | --- | --- | --- | --- |
| Yano 1989(33) | Asian (Japanese) | 7850 | 18 | 100 | 56.5 | Age, Alcohol, BP, Smoking, UA | RR | sTC | Fasting | HS(116) ICH(77) SAH(39) |
| Knekt 1991(34) | European (Finnish) | 42862 | 12 | 53.97 | 40.6 | Age | RR | sTC | Unspecified | SAH(187) |
| Gatchev 1993(35) | European (Swedish) | 54385 | 20.5 | 49.1 | 59.5 | Age, DBP, Follow-up period | RR | sTC | Unspecified | ICH mortality(347) SAH mortality(87) |
| Iribarren 1996(36) | American | 61756 | 10.7 | 46 | 54.2 | Age, BG/GLU, BMI, Education, Individual confounding conditions, Race, SBP, Smoking and drinking status | RR | sTC | Fasting | ICH(386) |
| Nakayama 1997(37) | Asian (Japanese) | 2302 | 15.5 | 41.8 | 56.4 | Age | RR | sTC | Non-fasting | ICH(19) |
| Leppälä 1999(38) | European (Finnish) | 28519 | 6 | 100 | 57.7 | Age, Alcohol, BMI, DM, Education, Physical activity, Previous heart disease, SBP, Smoking, α-tocopherol, β-carotene | RR | sHDL-C, sTC | Unspecified | ICH(112) SAH(85) |
| Okumura 1999(39) | Asian (Japanese) | 38053 | 3 | 46.9 | 65.6 | Age, SBP, UP, Sex | OR | sTC | Fasting | ICH(111) |
| Hart 2000(40) | European (Scottish) | 15267 | 20 | 45.8 | 54.5 | Age, CHD, DBP, Height, Sex, Smoking | RR | sTC | Unspecified | HS(90) |
| Suh 2001(41) | Asian (Korean) | 114793 | 6 | 100 | 45 | Age, Alcohol, BMI, BP, FBG, Smoking | RR | sTC | Fasting | ICH(372) SAH(98) |
| Engstrom 2002(42) | European (Swedish) | 6063 | 18.7 | 100 | 46.8 | Age | RR | sTC | Fasting | ICH(29) |
| Horenstein 2002(43) | American | 24343 | 13.9 | 0 | 52 | Age, BMI, DM, Education, Ethnicity, SBP, Smoking, Time period | RR | sTC | Unspecified | HS mortality(83) |
| Rodriguez(Fra.) 2002(44) | American | 1216 | 20 | 100 | 55.8 | Age, Alcohol, BMI, DM, Hypertension, Smoking | RR | sTC | Unspecified | HS(18) |
| Psaty 2004(45) | American | 4885 | 7.5 | 40 | 72.6 | Age, CVD, DM, SBP, Sex, Smoking | HR | sHDL-C, sLDL-C, sTC, sTG | Fasting | HS(104) |
| Zhang 2004(46) | Asian (Chinese) | 5092 | 13.5 | 100 | 45 | Age, BP, BMI, Smoking | RR | sTC | Fasting | HS(48) |
| Nakaya 2005(47) | Asian (Japanese) | 30832 | 5.39 | 31.6 | 57.7 | Age, DM, Hypertension, Sex, Smoking | RR | sHDL-C, sLDL-C, sTC, sTG | Unspecified | ICH(102) |
| Ebrahim 2006(48) | Asian (Korean) | 787442 | 10 | 84 | 42.3 | Age, Alcohol, Area of residence, BMI, DM, FBG, Height, Hypertension, Monthly pay, Physical activity, SBP, Sex, Smoking | HR | sTC | Fasting | HS(3947) |
| Wang 2006(49) | Asian (Chinese) | 30384 | 10 | 53.1 | 46.9 | Age, BMI, DM, HDL, Hypertension, SBP, Sex, Smoking | RR | sTC | Fasting | HS(128) |
| Sturgeon 2007(50) | American | 15792 | 12.2 | 44.1 | 59.3 | Age | RR | sHDL-C, sLDL-C, sTC, sTG | Fasting | ICH(135) |
| Liu 2008(51) | Asian (Chinese) | 30384 | 12 | 53.5 | 46.9 | Age, BMI, BP, DM, Sex, Smoking, sTC | RR | sHDL-C | Fasting | HS(159) |
| Holme 2009(52) | European (Swedish) | 148600 | 11.8 | 56.5 | 50.8 | Age, AMI, DM, Hypertension, Sex | HR | sHDL-C, sLDL-C, sTC, sTG | Fasting | HS(846) |
| Imamura 2009(53) | Asian (Japanese) | 2351 | 19 | 43 | 57.3 | Age, BMI, ECG, FBG, HDL, Regular exercise, SBP, Sex, Smoking and drinking status, TG | HR | sLDL-C | Fasting | HS(80) |
| Noda 2009(54) | Asian (Japanese) | 91219 | 10.3 | 33.8 | 58.6 | Age, BP, BMI, DM, GGT, Kidney dysfunction, Medication use(anti-hypertensive, anti-lipid), Sex, Smoking and drinking status | HR | sHDL-C, sLDL-C, sTC, sTG | Unspecified | HS mortality(454) ICH mortality(264) SAH mortality(190) |
| Bonaventure 2010(55) | European (French) | 8393 | 5 | 39 | 74.2 | Age, ApoE genotype, BP, DM, Excess weight, Medical history, Medicine use(aspirin, lipid-lowering), Sex, Smoking and drinking status, Study centre | HR | sTG | Fasting | HS(36) |
| Jiang 2010(56) | Asian (Chinese) | 10093 | 8.4 | 44.8 | 55.3 | Age, BMI, BP, DM, Education, Group, Heart disease, Hypertension, Sex, Smoking and drinking status | HR | sTC, sTG | Fasting | HS(216) |
| Tanabe 2010(57) | Asian (Japanese) | 22430 | 7.6 | 39.9 | 57.8 | Age, BMI, BP, DM, HDL, SBP, Sex, Smoking | RR | sTC | Unspecified | ICH(65) SAH(48) |
| Nago 2011(58) | Asian (Japanese) | 12241 | 11.9 | 39.2 | 55.3 | Age, BMI, HDL, SBP, Smoking and drinking status | HR | sTC | Unspecified | HS mortality(55) |
| Wieberdink 2011(59) | European (DusTCh) | 5773 | 9.7 | 42.9 | 67.3 | Age, Alcohol, BMI, DM, FBG, Medication use(anti-thrombotic, anti-hypertensive or lipid-lowering), SBP, Sex, SIC/RI, Smoking, Subcohort | HR | sHDL-C, sLDL-C, sTG | Fasting | ICH(45) |
| Cui 2012(60) | Asian (Japanese) | 33469 | 12 | 35 | 54.1 | Age, Area, BMI, HDL, Medication use(anti-hypertensive, anti-hyperlipidemia), SBP, Sex, Smoking and drinking status | HR | sTC | Non-fasting | HS(470) |
| Nagasawa 2012(61) | Asian (Japanese) | 65594 | 10.1 | 41.2 | 57 | Age, BMI, SBP, Sex, Smoking and drinking status | HR | sTC | Unspecified | ICH mortality(212) |
| Sandvei 2012(62) | European (Norwegian) | 92408 | 10.9 | 47.8 | 58.9 | Age, Alcohol, Number of hours since last meal, Sex, Smoking | HR | sHDL-C, sTC, sTG | Non-fasting | SAH(122) |
| Zhang 2012(63) | European (Finnish) | 58235 | 20.1 | 47.6 | 44.6 | Age, BMI, DM, Education, Family history of stroke, Medicine use(cholesterol-lowering agents), Physical activity, SBP, Smoking and drinking status, Study year | HR | sHDL-C, sTC | Fasting | ICH(497) SAH(332) |
| Korja 2013(64) | European (Finnish) | 64369 | 17.9 | 48.3 | 45 | Age, Hypertension, Premature stroke in mother, SBP, Sex, Smoking | HR | sTC | Fasting | SAH(437) |
| Suzuki 2013(65) | Asian (Japanese) | 151796 | 3 | 51 | 65.3 | Age, BMI, BP, Disorder(cardiac, hepatic, renal), DM, Sex, Smoking and drinking status | RR | sTC | Unspecified | ICH(344) |
| Stoekenbroek 2015(66) | European (England) | 21798 | 12.1 | 43.9 | 59 | Age, BMI, DM, HDL, SBP, Sex, Smoking | HR | sLDL-C | Non-fasting | HS(117) |
| Glasser 2016(67) | American | 23867 | 7.5 | 44.9 | 64.7 | Age, AF, BMI, DM, Education, History of heart disease, Income, Left ventricular hypertrophy, Medication use(anti-hypertensive, anti-lipid), Race, Region, SBP, Smoking | HR | sHDL-C, sLDL-C, sTC, sTG | Fasting | HS(77) |
| Saito 2017(68) | Asian (Japanese) | 30736 | 15 | 34.4 | 56.9 | Age, BMI, Cohort, DM, Hypertension, non-HDL-cholesterol, Sex, Smoking and drinking status | HR | sHDL-C | Non-fasting | ICH(475) SAH(173) |
| Hirata 2018(69) | Asian (Japanese) | 43407 | 12.1 | 48.6 | 57.1 | Age, BMI, DM, Medicine use(anti-hypertensive and anti-lipemic), non-HDL-cholesterol, SBP, Smoking and drinking status, Sports during leisure time | HR | sHDL-C | Unspecified | ICH mortality(117) |
| Lindbohm 2018(70) | European (Finnish) | 65521 | 23.2 | 48.4 | 43.3 | Age, Alcohol, BG/GLU, BMI, Drug use(cholesterol-lowering), SBP, Sex, SES, Smoking, Study year, Study area | HR | sHDL-C, sLDL-C, sTC | Fasting | SAH(543) |
| Yi 2018(71) | Asian (Korean) | 503340 | 10.4 | 54.3 | 52.9 | Age, Beneficiary income status, BMI, FBG, Physical activity, SBP, Sex, Smoking and drinking status | HR | sTC | Fasting | HS mortality(1184) ICH mortality(830) SAH mortality(354) |
| Zhang 2018(72) | Asian (Chinese) | 20954 | 21.8 | 51.5 | 47.4 | Age, BMI, Family history of CVD, FBG, HDL, Medicine use(anti-hypertensive, lipid-lowering), SBP, Sex, Smoking and drinking status | HR | sLDL-C | Fasting | HS(253) |
| Gu 2019(73) | Asian (Chinese) | 267500 | 8.6 | 59.6 | 50.4 | Age, Alcohol, BMI, Education level, Geographic region, Hypertension, Sex, Smoking | HR | sHDL-C, sLDL-C, sTC, sTG | Fasting | HS(2186) |
| Liu 2019(74) | Asian (Chinese) | 42005 | 3.6 | 61.9 | 42.3 | Age, BMI, DBP and SBP, DM, Family history of CVD, History of hypertension, Medicine use(lipid-lowering), Smoking and drinking status | HR | sHDL-C, sLDL-C, sTC, sTG | Fasting | HS(158) |
| Ma 2019(75) | Asian (Chinese) | 96043 | 9 | 79.9 | 51.3 | Age, ALT, Average monthly income, BMI, DBP and SBP, DM, Education, GFR, hs-CRP, Physical activity, Salt intake, Sex, Smoking and drinking status, Use of anti-hypertensive, lipid-lowering agents, aspirin and anti-coagulants | HR | sHDL-C, sLDL-C, sTC, sTG | Fasting | ICH(753) |
| Rist 2019(76) | American | 27937 | 19.3 | 0 | 54.7 | Age, Alcohol, BMI, DM, Hypertension, Medication use(cholesterol-lowering), Menopausal status and use, Physical activity, Randomized treatment assignment, Smoking status | RR | sHDL-C, sLDL-C, sTC, sTG | Fasting | HS(137) ICH(85) SAH(43) |
| Zheng 2019(77) | Asian (Chinese) | 5097 | 8.4 | 43.8 | 56.3 | Age, BMI, DM, Ethnicity, Medicine use(anti-hypertensive), SBP and DBP, Sex, Smoking and drinking status | HR | sHDL-C, sLDL-C, sTC, sTG | Fasting | HS(187) |
| Håheim 2020(78) | European (Norwegian) | 6530 | 12.5 | 100 | 69.1 | Age | HR | sHDL-C, sTC, sTG | Unspecified | ICH mortality(49) |
| Watanabe 2020(79) | Asian (Japanese) | 11027 | 10.7 | 38.9 | 55 | Age, BG/GLU, BMI, Medicine(anti-hypertensive, anti-hyperlipidemic, anti-diabetic), Menopausal status, non-HDL-cholesterol, Physical activity, SBP, Sex, Smoking and drinking status | HR | sHDL-C | Unspecified | ICH(95) SAH(51) |
| Al-Shoaibi 2023(80) | Asian (Japanese) | 8966 | 12 | 79 | 45.6 | Age, Alcohol, BMI, DM, HDL, Hypertension, Medicine use(anti-hypertensive and anti-dyslipidemia), Physical activity, Sex, Smoking, Survey year, TG | HR | sLDL-C | Fasting | ICH(25) |
| Bae 2022(81) | Asian (Korean) | 480830 | 10.3 | 50.7 | 42.4 | Age, Alcohol, BMI, CCI, DM, FBG Hypertension, Income, Medicine(aspirin and statin), Physical activity, SBP, Sex, Smoking | HR | sTC | Fasting | HS(2877) |
| Hirata 2022(82) | Asian (Japanese) | 6831 | 18 | 41.8 | 52.2 | Age, BMI, DM, HDL, Hypertension, Smoking and drinking status, sTC | HR | sTG | Non-fasting | ICH mortality(40) |
| Li 2022(83) | Asian (Chinese) | 96258 | 10.7 | 79.6 | 51.5 | Age, Average income, BMI, Education, FBG, GFR, Household income, hs-CRP, LDL, Medication use(lipid-lowering, anti-hypertensive agents, hypoglycemic), Marital status, Regular exercise, SBP, Sex, Smoking and drinking status, TG | HR | sHDL-C | Fasting | HS(869) |
| Yi(a) 2022(84) | Asian (Korean) | 15859501 | 8.8 | 52.2 | 47.4 | Age, BMI, FBG, Household income, LDL, Medication use(lipid-lowering), Physical activity, SBP, Sex, Smoking and drinking status, TG | HR | sHDL-C | Fasting | HS mortality(12000) ICH mortality(7809) SAH mortality(4191) |
| Yi(b) 2022(85) | Asian (Korean) | 14884975 | 8.8 | 52.7 | 46.6 | Age, BG/GLU, BMI, GFR, Household income, Physical activity, SBP, Sex, Smoking and drinking status | HR | sLDL-C | Fasting | HS mortality(15178) ICH mortality(9717) SAH mortality(5461) |
| Park 2024(86) | Asian (Korean) | 15672028 | 8.8 | 47.9 | 47.3 | Age, BMI, FBG, HDL, Household income, LDL, Medication use(lipid-lowering), Physical activity, SBP, Sex, Smoking and drinking status | HR | sTG | Fasting | HS mortality(16575) |

* Abbreviation in confounding variables: AF as atrial fibrillation; ALT as alanine aminotransferase; BG/GLU as blood glucose; BMI as body mass index; BP as blood pressure; CCI as Charlson comorbidity index; CHD as coronary heart disease; CVD as cardiovascular disease; DBP as diastolic blood pressure; DM as diabetes mellitus; ECG as electrocardiogram; FBG as fasting blood glucose; GGT as γ-glutamyl transpeptidase; GFR as glomerular filtration rate; Hct as hematocrit; HR as heart rate; hs-CRP as high sensitivity C-reactive protein; SBP as systolic blood pressure; SES as socioeconomic status; sHDL-C as serum high density lipoprotein cholesterol; SIC/RI as serum insulin concentration/ regular insulin; sLDL-C as serum low density lipoprotein cholesterol; sTC as serum total cholesterol; sTG as serum triglyceride; UA as uric acid; UP/Pro as urine protein.

**Supplemental Table 4** Independent and final results in quality assessment.

| **Study** | **XY** | **BZ** | **AH** | **WS** | **Assessor1** | **Assessor2** | **Final** |
| --- | --- | --- | --- | --- | --- | --- | --- |
| Yano 1989(33) | 9 |  |  | 9 | 9 | 9 | 9 |
| Knekt 1991(34) | 6 |  |  | 6 | 6 | 6 | 6 |
| Gatchev 1993(35) | 7 |  |  | 7 | 7 | 7 | 7 |
| Iribarren 1996(36) | 9 |  |  | 9 | 9 | 9 | 9 |
| Nakayama 1997(37) | 7 |  |  | 8 | 7 | 8 | 8 |
| Leppälä 1999(38) | 6 |  |  | 6 | 6 | 6 | 6 |
| Okumura 1999(39) | 7 |  |  | 7 | 7 | 7 | 7 |
| Hart 2000(40) | 7 |  |  | 7 | 7 | 7 | 7 |
| Suh 2001(41) | 8 |  |  | 8 | 8 | 8 | 8 |
| Engstrom 2002(42) | 8 |  |  | 8 | 8 | 8 | 8 |
| Horenstein 2002(43) | 8 |  |  | 8 | 8 | 8 | 8 |
| Rodriguez(Fra.) 2002(44) | 8 |  |  | 8 | 8 | 8 | 8 |
| Psaty 2004(45) | 8 |  |  | 8 | 8 | 8 | 8 |
| Zhang 2004(46) | 8 |  |  | 8 | 8 | 8 | 8 |
| Nakaya 2005(47) | 6 | 7 |  |  | 6 | 7 | 6 |
| Ebrahim 2006(48) | 9 | 9 |  |  | 9 | 9 | 9 |
| Wang 2006(49) | 7 | 7 |  |  | 7 | 7 | 7 |
| Sturgeon 2007(50) | 8 | 8 |  |  | 8 | 8 | 8 |
| Liu 2008(51) | 9 | 9 |  |  | 9 | 9 | 9 |
| Holme 2009(52) | 9 | 9 |  |  | 9 | 9 | 9 |
| Imamura 2009(53) | 9 | 9 |  |  | 9 | 9 | 9 |
| Noda 2009(54) | 8 | 8 |  |  | 8 | 8 | 8 |
| Bonaventure 2010(55) | 7 | 7 |  |  | 7 | 7 | 7 |
| Jiang 2010(56) | 8 | 9 |  |  | 8 | 9 | 8 |
| Tanabe 2010(57) | 7 | 7 |  |  | 7 | 7 | 7 |
| Nago 2011(58) | 8 | 8 |  |  | 8 | 8 | 8 |
| Wieberdink 2011(59) | 8 | 8 |  |  | 8 | 8 | 8 |
| Cui 2012(60) | 8 | 9 |  |  | 8 | 9 | 8 |
| Nagasawa 2012(61) | 8 | 8 |  |  | 8 | 8 | 8 |
| Sandvei 2012(62) |  | 9 | 9 |  | 9 | 9 | 9 |
| Zhang 2012(63) |  | 9 | 9 |  | 9 | 9 | 9 |
| Korja 2013(64) |  | 9 | 9 |  | 9 | 9 | 9 |
| Suzuki 2013(65) |  | 8 | 7 |  | 8 | 7 | 7 |
| Stoekenbroek 2015(66) |  | 9 | 9 |  | 9 | 9 | 9 |
| Glasser 2016(67) |  | 8 | 8 |  | 8 | 8 | 8 |
| Saito 2017(68) |  | 9 | 9 |  | 9 | 9 | 9 |
| Hirata 2018(69) |  | 7 | 8 |  | 7 | 8 | 7 |
| Lindbohm 2018(70) |  | 8 | 8 |  | 8 | 8 | 8 |
| Yi 2018(71) |  | 9 | 9 |  | 9 | 9 | 9 |
| Zhang 2018(72) |  | 9 | 9 |  | 9 | 9 | 9 |
| Gu 2019(73) |  | 8 | 8 |  | 8 | 8 | 8 |
| Liu 2019(74) |  | 8 | 8 |  | 8 | 8 | 8 |
| Ma 2019(75) |  | 8 | 8 |  | 8 | 8 | 8 |
| Rist 2019(76) |  | 9 | 9 |  | 9 | 9 | 9 |
| Zheng 2019(77) |  |  | 7 | 7 | 7 | 7 | 7 |
| Håheim 2020(78) |  |  | 7 | 7 | 7 | 7 | 7 |
| Watanabe 2020(79) |  |  | 8 | 8 | 8 | 8 | 8 |
| Al-Shoaibi 2023(80) |  |  | 9 | 9 | 9 | 9 | 9 |
| Bae 2022(81) |  |  | 8 | 8 | 8 | 8 | 8 |
| Hirata 2022(82) |  |  | 8 | 8 | 8 | 8 | 8 |
| Li 2022(83) |  |  | 9 | 9 | 9 | 9 | 9 |
| Yi(a) 2022(84) |  |  | 8 | 8 | 8 | 8 | 8 |
| Yi(b) 2022(85) |  |  | 8 | 8 | 8 | 8 | 8 |
| Park 2024(86) |  |  | 8 | 8 | 8 | 8 | 8 |

**Supplemental Table 5** Newcastle-Ottawa Scale for Prospective Cohort Studies.

| **Study** | **Selection** | | | | **Comparability** | **Outcome** | | | **Total** |
| --- | --- | --- | --- | --- | --- | --- | --- | --- | --- |
|  | **Representativeness of the exposed cohort** | **Selection of the non-exposed cohort** | **Ascertainment of exposure** | **Demonstration that outcome of interest was not present at start of study** | **Comparability of cohorts on the basis of the design or analysis** | **Assessment of outcome** | **Was follow-up long enough for outcomes to occur** | **Adequacy of follow-up of cohorts** |  |
| Yano 1989(33) | 1 | 1 | 1 | 1 | 2 | 1 | 1 | 1 | 9 |
| Knekt 1991(34) | 1 | 1 | 0 | 0 | 1 | 1 | 1 | 1 | 6 |
| Gatchev 1993(35) | 1 | 1 | 0 | 1 | 1 | 1 | 1 | 1 | 7 |
| Iribarren 1996(36) | 1 | 1 | 1 | 1 | 2 | 1 | 1 | 1 | 9 |
| Nakayama 1997(37) | 1 | 1 | 1 | 1 | 1 | 1 | 1 | 1 | 8 |
| Leppälä 1999(38) | 0 | 1 | 0 | 1 | 2 | 1 | 0 | 1 | 6 |
| Okumura 1999(39) | 1 | 1 | 1 | 1 | 1 | 1 | 0 | 1 | 7 |
| Hart 2000(40) | 1 | 1 | 0 | 0 | 2 | 1 | 1 | 1 | 7 |
| Suh 2001(41) | 1 | 1 | 1 | 1 | 2 | 1 | 0 | 1 | 8 |
| Engstrom 2002(42) | 1 | 1 | 1 | 1 | 1 | 1 | 1 | 1 | 8 |
| Horenstein 2002(43) | 1 | 1 | 0 | 1 | 2 | 1 | 1 | 1 | 8 |
| Rodriguez(Fra.) 2002(44) | 1 | 1 | 0 | 1 | 2 | 1 | 1 | 1 | 8 |
| Psaty 2004(45) | 1 | 1 | 1 | 1 | 2 | 1 | 0 | 1 | 8 |
| Zhang 2004(46) | 1 | 1 | 1 | 1 | 1 | 1 | 1 | 1 | 8 |
| Nakaya 2005(47) | 0 | 1 | 0 | 1 | 2 | 1 | 0 | 1 | 6 |
| Ebrahim 2006(48) | 1 | 1 | 1 | 1 | 2 | 1 | 1 | 1 | 9 |
| Wang 2006(49) | 1 | 1 | 1 | 0 | 2 | 1 | 1 | 0 | 7 |
| Sturgeon 2007(50) | 1 | 1 | 1 | 1 | 1 | 1 | 1 | 1 | 8 |
| Liu 2008(51) | 1 | 1 | 1 | 1 | 2 | 1 | 1 | 1 | 9 |
| Holme 2009(52) | 1 | 1 | 1 | 1 | 2 | 1 | 1 | 1 | 9 |
| Imamura 2009(53) | 1 | 1 | 1 | 1 | 2 | 1 | 1 | 1 | 9 |
| Noda 2009(54) | 1 | 1 | 0 | 1 | 2 | 1 | 1 | 1 | 8 |
| Bonaventure 2010(55) | 1 | 1 | 1 | 0 | 2 | 1 | 0 | 1 | 7 |
| Jiang 2010(56) | 1 | 1 | 1 | 1 | 2 | 1 | 0 | 1 | 8 |
| Tanabe 2010(57) | 1 | 1 | 0 | 1 | 2 | 1 | 0 | 1 | 7 |
| Nago 2011(58) | 1 | 1 | 0 | 1 | 2 | 1 | 1 | 1 | 8 |
| Wieberdink 2011(59) | 1 | 1 | 1 | 1 | 2 | 1 | 0 | 1 | 8 |
| Cui 2012(60) | 1 | 1 | 1 | 1 | 2 | 0 | 1 | 1 | 8 |
| Nagasawa 2012(61) | 1 | 1 | 0 | 1 | 2 | 1 | 1 | 1 | 8 |
| Sandvei 2012(62) | 1 | 1 | 1 | 1 | 2 | 1 | 1 | 1 | 9 |
| Zhang 2012(63) | 1 | 1 | 1 | 1 | 2 | 1 | 1 | 1 | 9 |
| Korja 2013(64) | 1 | 1 | 1 | 1 | 2 | 1 | 1 | 1 | 9 |
| Suzuki 2013(65) | 1 | 1 | 0 | 1 | 2 | 1 | 0 | 1 | 7 |
| Stoekenbroek 2015(66) | 1 | 1 | 1 | 1 | 2 | 1 | 1 | 1 | 9 |
| Glasser 2016(67) | 1 | 1 | 1 | 1 | 2 | 1 | 0 | 1 | 8 |
| Saito 2017(68) | 1 | 1 | 1 | 1 | 2 | 1 | 1 | 1 | 9 |
| Hirata 2018(69) | 1 | 1 | 0 | 0 | 2 | 1 | 1 | 1 | 7 |
| Lindbohm 2018(70) | 1 | 1 | 1 | 1 | 2 | 1 | 1 | 0 | 8 |
| Yi 2018(71) | 1 | 1 | 1 | 1 | 2 | 1 | 1 | 1 | 9 |
| Zhang 2018(72) | 1 | 1 | 1 | 1 | 2 | 1 | 1 | 1 | 9 |
| Gu 2019(73) | 1 | 1 | 1 | 1 | 2 | 1 | 0 | 1 | 8 |
| Liu 2019(74) | 1 | 1 | 1 | 1 | 2 | 1 | 0 | 1 | 8 |
| Ma 2019(75) | 1 | 1 | 1 | 1 | 2 | 1 | 0 | 1 | 8 |
| Rist 2019(76) | 1 | 1 | 1 | 1 | 2 | 1 | 1 | 1 | 9 |
| Zheng 2019(77) | 0 | 1 | 1 | 1 | 2 | 1 | 0 | 1 | 7 |
| Håheim 2020(78) | 1 | 1 | 0 | 1 | 1 | 1 | 1 | 1 | 7 |
| Watanabe 2020(79) | 1 | 1 | 0 | 1 | 2 | 1 | 1 | 1 | 8 |
| Al-Shoaibi 2023(80) | 1 | 1 | 1 | 1 | 2 | 1 | 1 | 1 | 9 |
| Bae 2022(81) | 1 | 1 | 1 | 1 | 2 | 1 | 1 | 0 | 8 |
| Hirata 2022(82) | 1 | 1 | 1 | 1 | 2 | 1 | 1 | 0 | 8 |
| Li 2022(83) | 1 | 1 | 1 | 1 | 2 | 1 | 1 | 1 | 9 |
| Yi(a) 2022(84) | 1 | 1 | 1 | 1 | 2 | 1 | 0 | 1 | 8 |
| Yi(b) 2022(85) | 1 | 1 | 1 | 1 | 2 | 1 | 0 | 1 | 8 |
| Park 2024(86) | 1 | 1 | 1 | 1 | 2 | 1 | 0 | 1 | 8 |

**Supplemental Table 6** Summary of univariate meta-regression analysis for continuous variable analysis on relationship between serum lipids and risk of hemorrhagic stroke.

| Univariate |  | **Total HS** |  |  |  | **ICH** |  |  |  | **SAH** |  |
| --- | --- | --- | --- | --- | --- | --- | --- | --- | --- | --- | --- |
|  | **RR** | **95%CI** | ***P*** |  | **RR** | **95%CI** | ***P*** |  | **RR** | **95%CI** | ***P*** |
| **sTC** |  |  |  |  |  |  |  |  |  |  |  |
| **Population Sourse** |  |  |  |  |  |  |  |  |  |  |  |
| American |  | Reference |  |  |  | Reference |  |  |  | Reference |  |
| Asian | 0.9268 | 0.8075~1.0638 | 0.28 |  | 0.9458 | 0.8340~1.0724 | 0.38 |  | 1.0881 | 0.7171~1.6510 | 0.69 |
| European | 0.8735 | 0.7498~1.0175 | 0.08 |  | 0.9221 | 0.7961~1.0680 | 0.28 |  | 1.2129 | 0.8049~1.8277 | 0.36 |
| **Participant Number** | 1.0000 | 1.0000~1.0000 | 0.79 |  | 1.0000 | 1.0000~1.0000 | 0.91 |  | 1.0000 | 1.0000~1.0000 | 0.85 |
| **Follow-up Duration** | 1.0006 | 0.9920~1.0093 | 0.89 |  | 1.0037 | 0.9972~1.0101 | 0.26 |  | 1.0120 | 1.0025~1.0217 | 0.01 |
| **Male Proportion** | 0.9979 | 0.9966~0.9992 | <0.01 |  | 0.9988 | 0.9972~1.0003 | 0.12 |  | 0.9986 | 0.9959~1.0012 | 0.29 |
| **Population Age** | 0.9943 | 0.9892~0.9995 | 0.03 |  | 0.9962 | 0.9917~1.0008 | 0.11 |  | 0.9883 | 0.9813~0.9953 | <0.01 |
| **Fasting State** |  |  |  |  |  |  |  |  |  |  |  |
| Fasting |  | Reference |  |  |  | Reference |  |  |  | Reference |  |
| Non-fasting | 1.0332 | 0.8616~1.2390 | 0.72 |  | 1.1225 | 0.8110~1.5537 | 0.49 |  | 0.9046 | 0.7179~1.1400 | 0.40 |
| Unspecified | 0.9233 | 0.8337~1.0226 | 0.13 |  | 0.9195 | 0.8578~0.9857 | 0.02 |  | 0.8642 | 0.7454~1.0020 | 0.05 |
| **Endpoint** |  |  |  |  |  |  |  |  |  |  |  |
| Incidence |  | Reference |  |  |  | Reference |  |  |  | Reference |  |
| Mortality | 1.0121 | 0.9046~1.1323 | 0.83 |  | 0.9688 | 0.8912~1.0531 | 0.46 |  | 0.9220 | 0.7715~1.1020 | 0.37 |
| **Newcastle-Ottawa Scale** | 1.0380 | 0.9872~1.0913 | 0.15 |  | 1.0333 | 0.9918~1.0764 | 0.12 |  | 1.0454 | 0.9864~1.1079 | 0.13 |
| **sLDL-C** |  |  |  |  |  |  |  |  |  |  |  |
| **Population Sourse** |  |  |  |  |  |  |  |  |  |  |  |
| American |  | Reference |  |  |  | Reference |  |  |  |  |  |
| Asian | 0.9404 | 0.7763~1.1392 | 0.53 |  | 0.7573 | 0.5828~0.9840 | 0.04 |  |  |  |  |
| European | 0.9381 | 0.7463~1.1792 | 0.58 |  | 1.0472 | 0.6730~1.6293 | 0.84 |  |  |  |  |
| **Participant Number** | 1.0000 | 1.0000~1.0000 | 0.17 |  | 1.0000 | 1.0000~1.0000 | 0.82 |  |  |  |  |
| **Follow-up Duration** | 0.9979 | 0.9847~1.0114 | 0.76 |  | 1.0108 | 0.9770~1.0458 | 0.54 |  |  |  |  |
| **Male Proportion** | 1.0011 | 0.9969~1.0054 | 0.60 |  | 0.9964 | 0.9944~0.9985 | <0.01 |  |  |  |  |
| **Population Age** | 0.9980 | 0.9888~1.0073 | 0.67 |  | 1.0168 | 0.9948~1.0394 | 0.14 |  |  |  |  |
| **Fasting State** |  |  |  |  |  |  |  |  |  |  |  |
| Fasting |  | Reference |  |  |  | Reference |  |  |  |  |  |
| Non-fasting | 0.9335 | 0.7314~1.1915 | 0.58 |  | - | - | - |  |  |  |  |
| Unspecified | 0.8062 | 0.6600~0.9848 | 0.03 |  | 0.9625 | 0.6962~1.3306 | 0.82 |  |  |  |  |
| **Endpoint** |  |  |  |  |  |  |  |  |  |  |  |
| Incidence |  | Reference |  |  |  | Reference |  |  |  |  |  |
| Mortality | 0.8620 | 0.8065~0.9213 | <0.01 |  | 0.8792 | 0.6680~1.1571 | 0.36 |  |  |  |  |
| **Newcastle-Ottawa Scale** | 1.0089 | 0.8953~1.1368 | 0.89 |  | 0.9293 | 0.7787~1.1091 | 0.42 |  |  |  |  |
| **sHDL-C** |  |  |  |  |  |  |  |  |  |  |  |
| **Population Sourse** |  |  |  |  |  |  |  |  |  |  |  |
| American |  | Reference |  |  |  | Reference |  |  |  | Reference |  |
| Asian | 0.8446 | 0.5376~1.3267 | 0.46 |  | 0.6197 | 0.3697~1.0387 | 0.07 |  | 0.7387 | 0.3138~1.7391 | 0.49 |
| European | 0.7628 | 0.4530~1.2844 | 0.31 |  | 0.7772 | 0.4207~1.4359 | 0.42 |  | 0.5700 | 0.2419~1.3430 | 0.20 |
| **Participant Number** | 1.0000 | 1.0000~1.0000 | 0.78 |  | 1.0000 | 1.0000~1.0000 | 0.78 |  | 1.0000 | 1.0000~1.0000 | 0.29 |
| **Follow-up Duration** | 1.0075 | 0.9719~1.0445 | 0.68 |  | 1.0128 | 0.9655~1.0624 | 0.60 |  | 0.9767 | 0.9668~0.9867 | <0.01 |
| **Male Proportion** | 1.0011 | 0.9935~1.0087 | 0.79 |  | 1.0015 | 0.9933~1.0098 | 0.72 |  | 0.9910 | 0.9793~1.0028 | 0.14 |
| **Population Age** | 1.0037 | 0.9811~1.0269 | 0.75 |  | 1.0065 | 0.9745~1.0396 | 0.69 |  | 1.0140 | 0.9828~1.0461 | 0.38 |
| **Fasting State** |  |  |  |  |  |  |  |  |  |  |  |
| Fasting |  | Reference |  |  |  | Reference |  |  |  | Reference |  |
| Non-fasting | 1.0882 | 0.6394~1.8521 | 0.76 |  | 1.0546 | 0.5617~1.9800 | 0.87 |  | 1.2248 | 0.7478~2.0061 | 0.42 |
| Unspecified | 0.6652 | 0.4180~1.0586 | 0.09 |  | 0.6933 | 0.4771~1.0074 | 0.05 |  | 0.7352 | 0.4041~1.3379 | 0.31 |
| **Endpoint** |  |  |  |  |  |  |  |  |  |  |  |
| Incidence |  | Reference |  |  |  | Reference |  |  |  | Reference |  |
| Mortality | 0.9335 | 0.5709~1.5263 | 0.78 |  | 0.8004 | 0.5461~1.1730 | 0.25 |  | 1.2262 | 0.8388~1.7925 | 0.29 |
| **Newcastle-Ottawa Scale** | 1.1412 | 0.9556~1.3628 | 0.14 |  | 1.1697 | 0.9557~1.4316 | 0.13 |  | 1.2796 | 0.9713~1.6857 | 0.08 |
| **TG** |  |  |  |  |  |  |  |  |  |  |  |
| **Population Sourse** |  |  |  |  |  |  |  |  |  |  |  |
| American |  | Reference |  |  |  | Reference |  |  |  |  |  |
| Asian | 1.3756 | 1.1339~1.6690 | <0.01 |  | 1.7518 | 1.2973~2.3655 | <0.01 |  |  |  |  |
| European | 1.3855 | 1.1209~1.7125 | <0.01 |  | 1.3673 | 0.9289~2.0125 | 0.11 |  |  |  |  |
| **Participant Number** | 1.0000 | 1.0000~1.0000 | 0.71 |  | 1.0000 | 1.0000~1.0000 | 0.56 |  |  |  |  |
| **Follow-up Duration** | 0.9916 | 0.9784~1.0049 | 0.21 |  | 0.9923 | 0.9487~1.0378 | 0.73 |  |  |  |  |
| **Male Proportion** | 1.0064 | 1.0017~1.0112 | <0.01 |  | 1.0017 | 0.9947~1.0088 | 0.63 |  |  |  |  |
| **Population Age** | 0.9917 | 0.9846~0.9989 | 0.02 |  | 0.9800 | 0.9511~1.0098 | 0.19 |  |  |  |  |
| **Fasting State** |  |  |  |  |  |  |  |  |  |  |  |
| Fasting |  | Reference |  |  |  | Reference |  |  |  |  |  |
| Non-fasting | - | - | - |  | 1.5033 | 0.8226~2.7473 | 0.19 |  |  |  |  |
| Unspecified | - | - | - |  | 1.2293 | 0.8333~1.8135 | 0.30 |  |  |  |  |
| **Endpoint** |  |  |  |  |  |  |  |  |  |  |  |
| Incidence |  | Reference |  |  |  | Reference |  |  |  |  |  |
| Mortality | 0.9936 | 0.9613~1.0270 | 0.71 |  | 1.2294 | 0.8464~1.7858 | 0.28 |  |  |  |  |
| **Newcastle-Ottawa Scale** | 0.9921 | 0.9139~1.0770 | 0.85 |  | 0.8908 | 0.7179~1.1053 | 0.29 |  |  |  |  |

**P* value was marked with red if <0.05, and marked with orange if <0.10.

**Supplemental Table 7** Summary of multivariate meta-regression analysis for continuous variable analysis on relationship between serum lipids and risk of hemorrhagic stroke.

| Multivariate |  | **Total HS** |  |  | Multivariate |  | **ICH** |  |  | Multivariate |  | **SAH** |  |
| --- | --- | --- | --- | --- | --- | --- | --- | --- | --- | --- | --- | --- | --- |
|  | **RR** | **95%CI** | ***P*** |  |  | **RR** | **95%CI** | ***P*** |  |  | **RR** | **95%CI** | ***P*** |
| **sTC** |  |  |  |  |  |  |  |  |  |  |  |  |  |
| **Population Sourse** |  |  |  |  | **Fasting State** |  |  |  |  | **Follow-up Duration** | 1.0047 | 0.9943~1.0153 | 0.38 |
| American |  | Reference |  |  | Fasting |  | Reference |  |  | **Population Age** | 0.9905 | 0.9820~0.9991 | 0.03 |
| Asian | 0.9413 | 0.8214~1.0787 | 0.38 |  | Non-fasting | 1.1225 | 0.8110~1.5537 | 0.49 |  | **Fasting State** |  |  |  |
| European | 0.9176 | 0.8019~1.0501 | 0.21 |  | Unspecified | 0.9195 | 0.8578~0.9857 | 0.02 |  | Fasting |  | Reference |  |
| **Male Proportion** | 0.9979 | 0.9967~0.9992 | <0.01 |  |  |  |  |  |  | Non-fasting | 1.0316 | 0.8291~1.2836 | 0.78 |
| **Population Age** | 0.9927 | 0.9883~0.9971 | <0.01 |  |  |  |  |  |  | Unspecified | 0.9194 | 0.7977~1.0598 | 0.25 |
| **sLDL** |  |  |  |  |  |  |  |  |  |  |  |  |  |
| **Fasting State** |  |  |  |  | **Population Sourse** |  |  |  |  |  |  |  |  |
| Fasting |  | Reference |  |  | American |  | Reference |  |  |  |  |  |  |
| Non-fasting | 0.9102 | 0.7456~1.1111 | 0.36 |  | Asian | 0.9402 | 0.6001~1.4730 | 0.79 |  |  |  |  |  |
| Unspecified | 0.9048 | 0.7849~1.0430 | 0.17 |  | European | 1.2352 | 0.7110~2.1459 | 0.45 |  |  |  |  |  |
| **Endpoint** |  |  |  |  | **Male Proportion** | 0.9962 | 0.9902~1.0021 | 0.20 |  |  |  |  |  |
| Incidence |  | Reference |  |  |  |  |  |  |  |  |  |  |  |
| Mortality | 0.8688 | 0.8186~0.9222 | <0.01 |  |  |  |  |  |  |  |  |  |  |
| **sHDL** |  |  |  |  |  |  |  |  |  |  |  |  |  |
| **Fasting State** |  |  |  |  | **Population Sourse** |  |  |  |  | **Follow-up Duration** | 0.9751 | 0.9652~0.9851 | <0.01 |
| Fasting |  | Reference |  |  | American |  | Reference |  |  | **Newcastle-Ottawa Scale** | 1.3070 | 1.0358~1.6491 | 0.02 |
| Non-fasting | 1.0882 | 0.6394~1.8520 | 0.76 |  | Asian | 0.6733 | 0.3976~1.1402 | 0.14 |  |  |  |  |  |
| Unspecified | 0.6652 | 0.4180~1.0586 | 0.09 |  | European | 0.8734 | 0.4728~1.6136 | 0.67 |  |  |  |  |  |
|  |  |  |  |  | **Fasting State** |  |  |  |  |  |  |  |  |
|  |  |  |  |  | Fasting |  | Reference |  |  |  |  |  |  |
|  |  |  |  |  | Non-fasting | 1.2142 | 0.6544~2.2527 | 0.54 |  |  |  |  |  |
|  |  |  |  |  | Unspecified | 0.7500 | 0.5148~1.0927 | 0.13 |  |  |  |  |  |
| **sTG** |  |  |  |  |  |  |  |  |  |  |  |  |  |
| **Population Sourse** |  |  |  |  | **Population Sourse** |  |  |  |  |  |  |  |  |
| American |  | Reference |  |  | American |  | Reference |  |  |  |  |  |  |
| Asian | 1.2618 | 0.9558~1.6658 | 0.10 |  | Asian | 1.7518 | 1.2973~2.3655 | <0.01 |  |  |  |  |  |
| European | 1.2662 | 0.9563~1.6765 | 0.10 |  | European | 1.3673 | 0.9289~2.0125 | 0.11 |  |  |  |  |  |
| **Male Proportion** | 1.0020 | 0.9989~1.0051 | 0.21 |  |  |  |  |  |  |  |  |  |  |
| **Population Age** | 0.9974 | 0.9883~1.0066 | 0.58 |  |  |  |  |  |  |  |  |  |  |

**P* value was marked with red if <0.05, and marked with orange if <0.10.

**Supplemental Table 8** Summary of dose-response relationship between serum lipids and hemorrhagic stroke.

| **Expose** | **End** | **Model** | **Number** | **χ^2^_trend_** | **p_trend_** | **I^2^** | **p_Het_** | **χ^2^_non_** | **p_non_** | **logLik** | **AIC** | **BIC** |
| --- | --- | --- | --- | --- | --- | --- | --- | --- | --- | --- | --- | --- |
| sTC | Total HS | Model1 | 20 | 12.656 | <0.01 | 64.6% | <0.01 | - | - | 11.574 | -19.148 | -17.259 |
|  |  | Model2 | 20 | 13.776 | <0.01 | 54.3% | <0.01 | 1.636 | 0.201 | 2.777 | 4.446 | 12.634 |
|  | ICH | Model1 | 15 | 25.517 | <0.01 | 50.9% | 0.012 | - | - | 7.192 | -10.384 | -9.106 |
|  |  | Model2 | 15 | 64.644 | <0.01 | 53.3% | <0.01 | 8.509 | <0.01 | 0.919 | 8.161 | 14.822 |
|  | SAH | Model1 | 10 | 0.441 | 0.507 | 49.2% | 0.039 | - | - | 3.246 | -2.492 | -2.098 |
|  |  | Model2 | 10 | 0.563 | 0.754 | 10.7% | 0.324 | 0.180 | 0.671 | -4.335 | 18.669 | 23.121 |
| sLDL-C | Total HS | Model1 | 9 | 6.717 | <0.01 | 86.6% | <0.01 | - | - | 4.729 | -5.458 | -5.299 |
|  |  | Model2 | 9 | 17.035 | <0.01 | 85.1% | <0.01 | 2.180 | 0.140 | -0.520 | 11.039 | 14.902 |
|  | ICH | Model1 | 9 | 11.222 | <0.01 | 67.6% | <0.01 | - | - | 0.067 | 3.867 | 4.025 |
|  |  | Model2 | 9 | 497.764 | <0.01 | 64.0% | <0.01 | 5.832 | 0.016 | -7.182 | 24.364 | 28.227 |
|  | SAH | Model1 | 3 | 4.024 | 0.045 | 20.6% | 0.284 | - | - | 0.961 | 2.078 | -0.536 |
|  |  | Model2 | 3 | 23.024 | <0.01 | 0% | 0.744 | 22.097 | <0.01 | 0.549 | 8.903 | 5.834 |
| sHDL-C | Total HS | Model1 | 11 | 0.529 | 0.467 | 82.4% | <0.01 | - | - | -3.670 | 11.341 | 11.946 |
|  |  | Model2 | 11 | 83.379 | <0.01 | 62.4% | <0.01 | 9.893 | <0.01 | -16.596 | 43.193 | 48.171 |
|  | ICH | Model1 | 13 | 0.227 | 0.634 | 64.0% | <0.01 | - | - | -5.503 | 15.005 | 15.975 |
|  |  | Model2 | 13 | 34.201 | <0.01 | 49.0% | <0.01 | 20.480 | <0.01 | -26.750 | 63.499 | 69.390 |
|  | SAH | Model1 | 6 | 4.395 | 0.036 | 29.0% | 0.217 | - | - | -2.805 | 9.609 | 8.828 |
|  |  | Model2 | 6 | 19.687 | <0.01 | 0% | 0.650 | 5.968 | 0.015 | -11.917 | 33.834 | 35.347 |
| sTG | Total HS | Model1 | 7 | 0.103 | 0.749 | 48.4% | 0.071 | - | - | 0.152 | 3.697 | 3.280 |
|  |  | Model2 | 7 | 0.719 | 0.698 | 26.1% | 0.181 | 0.348 | 0.555 | -8.926 | 27.852 | 30.276 |
|  | ICH | Model1 | 8 | 2.438 | 0.118 | 67.7% | <0.01 | - | - | -2.831 | 9.661 | 9.553 |
|  |  | Model2 | 8 | 3.262 | 0.196 | 30.5% | 0.125 | 1.816 | 0.178 | -9.553 | 29.105 | 32.301 |
|  | SAH | Model1 | 1 | 4.468 | 0.035 | - | - | - | - | -0.657 | 3.314 | 2.413 |
|  |  | Model2 | 1 | 5.443 | 0.066 | - | - | 0.976 | 0.323 | -0.169 | 4.339 | 2.536 |

*χ^2^_trend_ as χ^2^-value for trends; p_trend_ as *P* value for trends; p_Het_ as *P* value for heterogeneity; χ^2^_non_ as χ^2^-value for Wald test; p_non_ as *P* value for Wald test; logLik as log-likelihood value.

**Supplemental Table 9** Certainty assessment of analyses for relationship between serum lipids and risk of hemorrhagic stroke.

| **Exposure** | **Endpoint** | **№ of studies** | **Study design** | **Risk of bias** | **Inconsistency** | **Indirectness** | **Imprecision** | **Other considerations** | **Relative (95% CI)** | **Certainty** |
| --- | --- | --- | --- | --- | --- | --- | --- | --- | --- | --- |
| sTC | HS | 25 | prospective cohort study | serious^a^ | serious^b^ | not serious | not serious | all plausible residual confounding would reduce the demonstrated effect dose response gradient^c^ | **RR 0.94** (0.90 to 0.97) | ⨁⨁◯◯ Low^a,b,c^ |
| sTC | ICH | 18 | prospective cohort study | serious^a^ | not serious | not serious | not serious | all plausible residual confounding would reduce the demonstrated effect dose response gradient^c^ | **RR 0.90** (0.87 to 0.93) | ⨁⨁⨁◯ Moderate^a,c^ |
| sTC | SAH | 12 | prospective cohort study | serious^a^ | serious^b^ | not serious | not serious | publication bias strongly suspected all plausible residual confounding would suggest spurious effect, while no effect was observed^c,d^ | **RR 0.97** (0.91 to 1.04) | ⨁◯◯◯ Very low^a,b,c,d^ |
| sLDL-C | HS | 12 | prospective cohort study | not serious | very serious^b^ | not serious | serious^e^ | all plausible residual confounding would reduce the demonstrated effect dose response gradient^c^ | **RR 0.92** (0.86 to 0.98) | ⨁◯◯◯ Very low^b,c,e^ |
| sLDL-C | ICH | 9 | prospective cohort study | not serious | serious^b^ | not serious | not serious | dose response gradient | **RR 0.83** (0.74 to 0.94) | ⨁⨁◯◯ Low^b^ |
| sLDL-C | SAH | 4 | prospective cohort study | not serious | very serious^b^ | not serious |  |  | **RR 0.94** (0.82 to 1.07) | -^b^ |
| sHDL-C | HS | 14 | prospective cohort study | not serious | very serious^b^ | not serious | serious^e^ | dose response gradient | **RR 0.95** (0.82 to 1.10) | ⨁◯◯◯ Very low^b,e^ |
| sHDL-C | ICH | 14 | prospective cohort study | serious^a^ | serious^b^ | not serious | not serious | dose response gradient | **RR 0.96** (0.80 to 1.16) | ⨁◯◯◯ Very low^a,b^ |
| sHDL-C | SAH | 8 | prospective cohort study | not serious | serious^b^ | not serious | not serious | all plausible residual confounding would reduce the demonstrated effect dose response gradient | **RR 0.77** (0.63 to 0.92) | ⨁⨁⨁◯ Moderate^b^ |
| sTG | HS | 10 | prospective cohort study | not serious | not serious | not serious | not serious | none | **RR 1.00** (0.99 to 1.01) | ⨁⨁◯◯ Low |
| sTG | ICH | 8 | prospective cohort study | serious^a^ | serious^b^ | not serious | not serious | all plausible residual confounding would suggest spurious effect, while no effect was observed^c^ | **RR 0.86** (0.73 to 1.02) | ⨁◯◯◯ Very low^a,b,c^ |
| sTG | SAH | 2 | prospective cohort study | not serious | very serious^b^ | not serious |  |  | **RR 0.74** (0.35 to 1.55) | -^b^ |

a. Risk of bias was assessed by the average NOS points of the studies included, with a score of 8 or more as not serious bias, a score of 7 as serious bias, and a score of 6 or lower as very serious bias.

b. Inconsistency was assessed by the heterogeneity detected in the random effects model, which was regarded as no when no or low heterogeneity (<50%) was detected, as serious when moderate heterogeneity (50%~75%) was detected, and as very serious when high heterogeneity (>75%) was detected.

c. Plausible residual confounding was assessed by multivariate meta-regression, which was regarded as no when no significant variable was detected, as "would reduce demonstrated effect" when any significant variable was detected with significant therapeutic effect demonstrated, and as "would suggest spurious effect" when any significant variable was detected with no significant therapeutic effect demonstrated.

d. Publication bias was assessed by Egger’s test, which was regarded as strongly suspected when significant bias on publication was detected (*P*<0.01), or as unlikely otherwise.

e. Imprecision was assessed by the sensitivity analysis, which was regarded as no when the result was stable, as serious when significantly influenced by one single study, and as very serious when significantly influenced by several studies.

**Supplemental Figure 1** Continuous variable analysis on relationship between serum total cholesterol and risk of total hemorrhagic stroke; **A)** Baujat plot for heterogeneity analysis, **B)** Funnel plot for bias on publication, **C)** Funnel plot for bias on publication after trim-and-fill method, **D)** Bubble plot for meta-regression on publish years, **E)** Forest plot for risk ratio after trim-and-fill method, **F)** Forest plot for risk ratio after one-by-one exclusion.





**Supplemental Figure 2** Forest plots of subgroup analysis of continuous variable analysis on risk ratio of serum total cholesterol on total hemorrhagic stroke. p_β_ represents *P* value for treatment estimates within subgroups; p_i_ represents *P* value for heterogeneity within subgroups; p_e_ as represents *P* value for heterogeneity between subgroups.


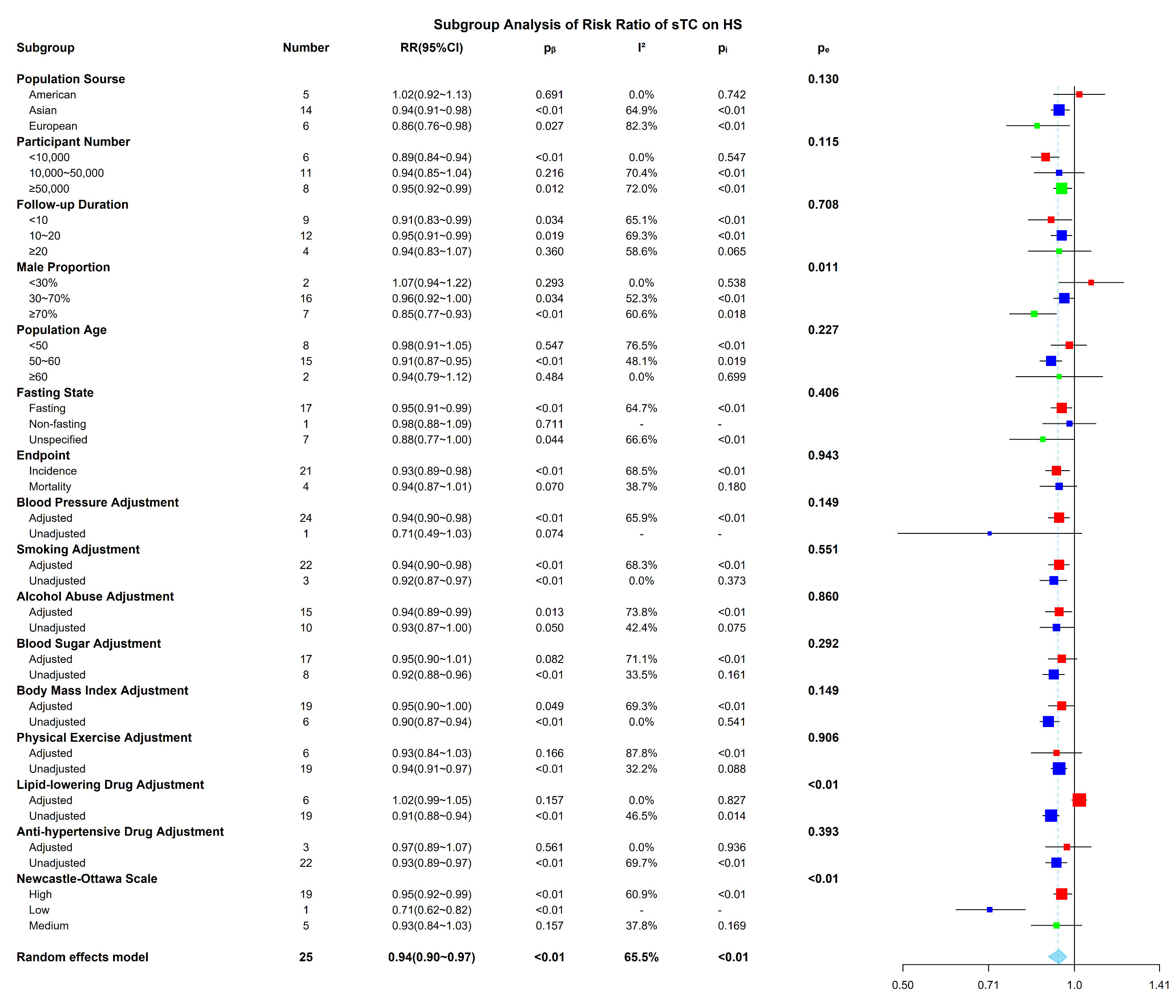


**Supplemental Figure 3** Continuous variable analysis on relationship between serum total cholesterol and risk of intracerebral hemorrhage; **A)** Baujat plot for heterogeneity analysis, **B)** Funnel plot for bias on publication, **C)** Funnel plot for bias on publication after trim-and-fill method, **D)** Bubble plot for meta-regression on publish years, **E)** Forest plot for risk ratio after trim-and-fill method, **F)** Forest plot for risk ratio after one-by-one exclusion.


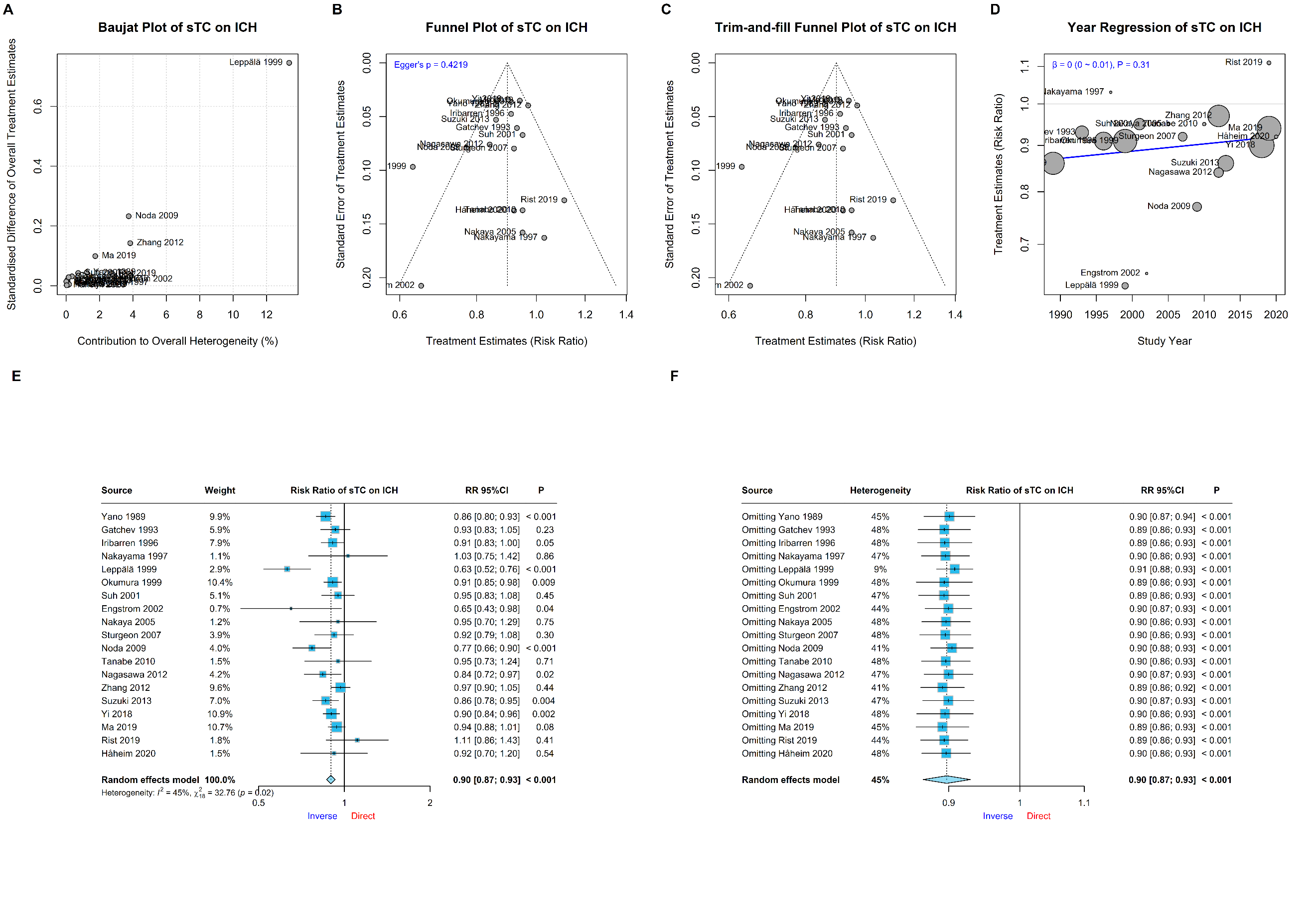


**Supplemental Figure 4** Forest plots of subgroup analysis of continuous variable analysis on risk ratio of serum total cholesterol on intracerebral hemorrhage. p_β_ represents *P* value for treatment estimates within subgroups; p_i_ represents *P* value for heterogeneity within subgroups; p_e_ as represents *P* value for heterogeneity between subgroups.


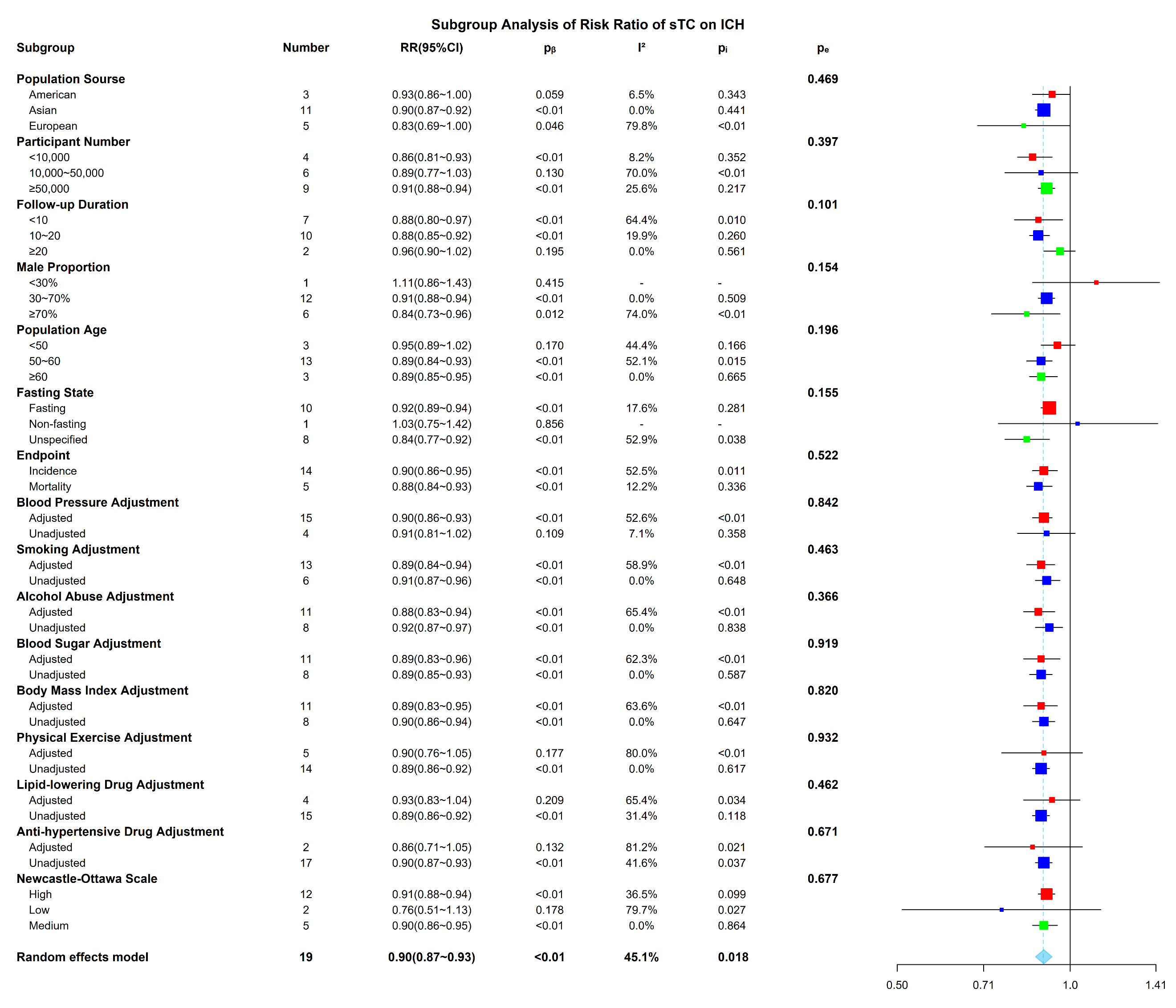


**Supplemental Figure 5** Continuous variable analysis on relationship between serum total cholesterol and risk of subarachnoid hemorrhage; **A)** Baujat plot for heterogeneity analysis, **B)** Funnel plot for bias on publication, **C)** Funnel plot for bias on publication after trim-and-fill method, **D)** Bubble plot for meta-regression on publish years, **E)** Forest plot for risk ratio after trim-and-fill method, **F)** Forest plot for risk ratio after one-by-one exclusion.


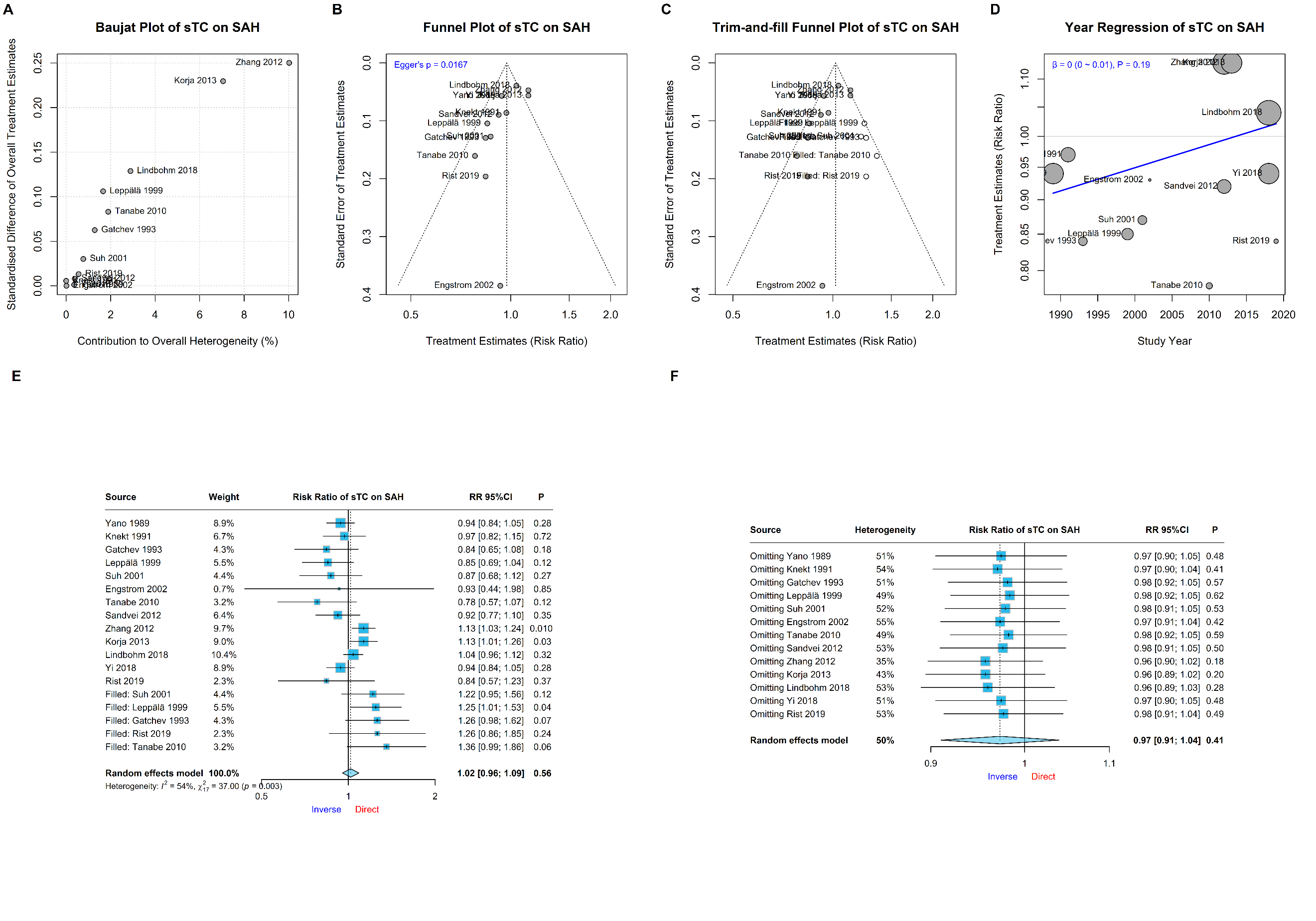


**Supplemental Figure 6** Forest plots of subgroup analysis of continuous variable analysis on risk ratio of serum total cholesterol on subarachnoid hemorrhage. p_β_ represents *P* value for treatment estimates within subgroups; p_i_ represents *P* value for heterogeneity within subgroups; p_e_ as represents *P* value for heterogeneity between subgroups.


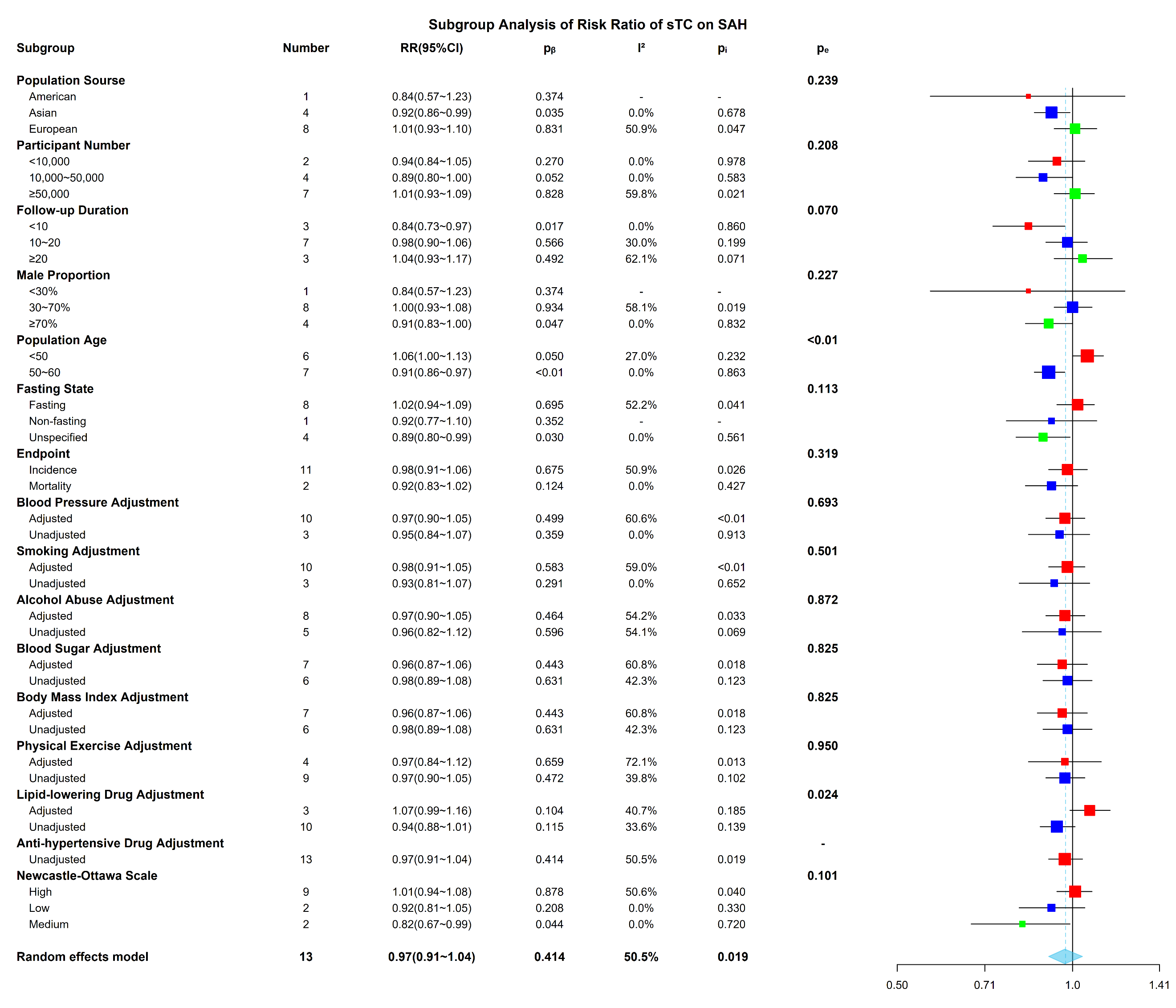


**Supplemental Figure 7** Continuous variable analysis on relationship between serum low-density lipoprotein cholesterol and risk of total hemorrhagic stroke; **A)** Baujat plot for heterogeneity analysis, **B)** Funnel plot for bias on publication, **C)** Funnel plot for bias on publication after trim-and-fill method, **D)** Bubble plot for meta-regression on publish years, **E)** Forest plot for risk ratio after trim-and-fill method, **F)** Forest plot for risk ratio after one-by-one exclusion.


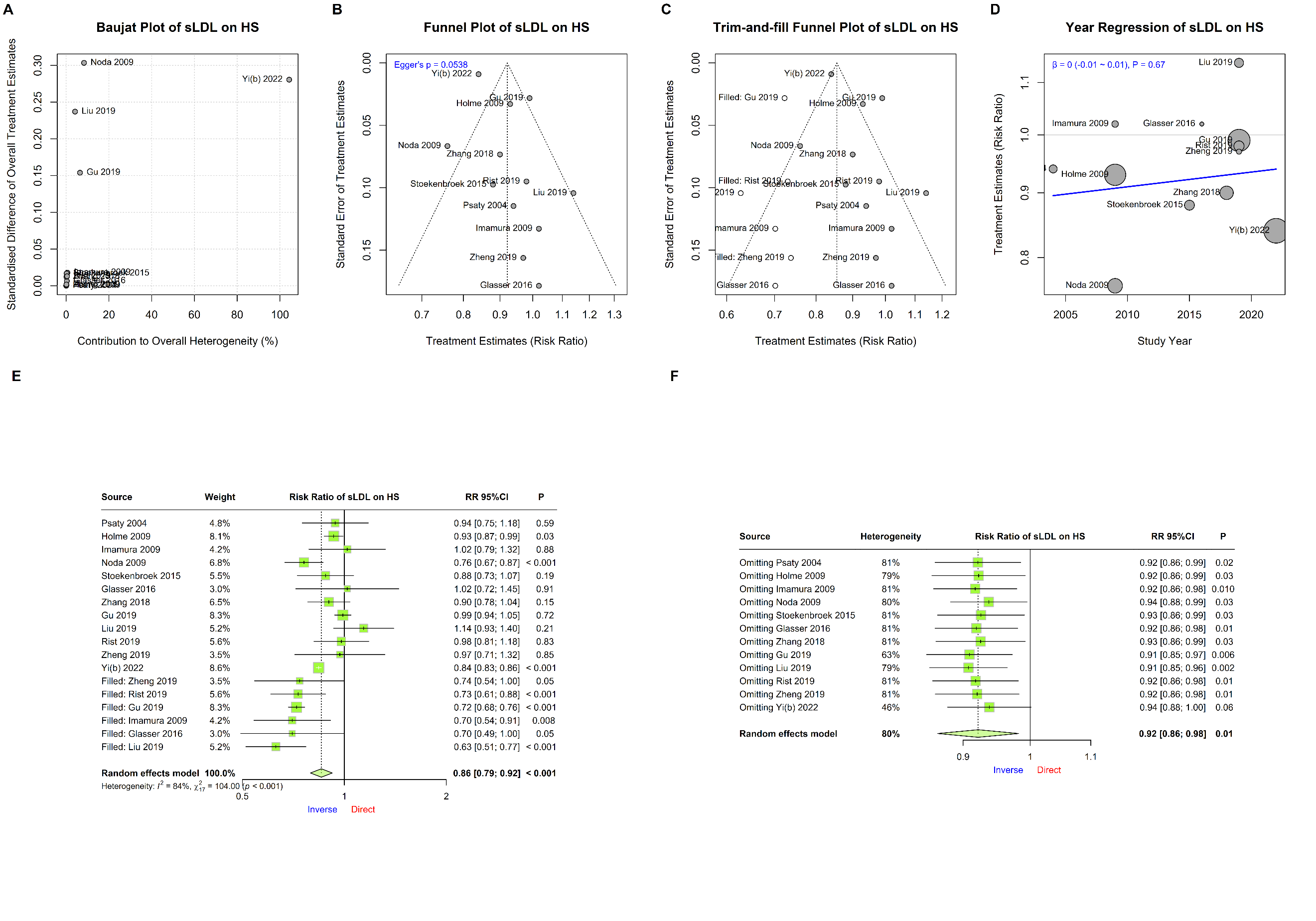


**Supplemental Figure 8** Forest plots of subgroup analysis of continuous variable analysis on risk ratio of serum low-density lipoprotein cholesterol on total hemorrhagic stroke. p_β_ represents *P* value for treatment estimates within subgroups; p_i_ represents *P* value for heterogeneity within subgroups; p_e_ as represents *P* value for heterogeneity between subgroups.


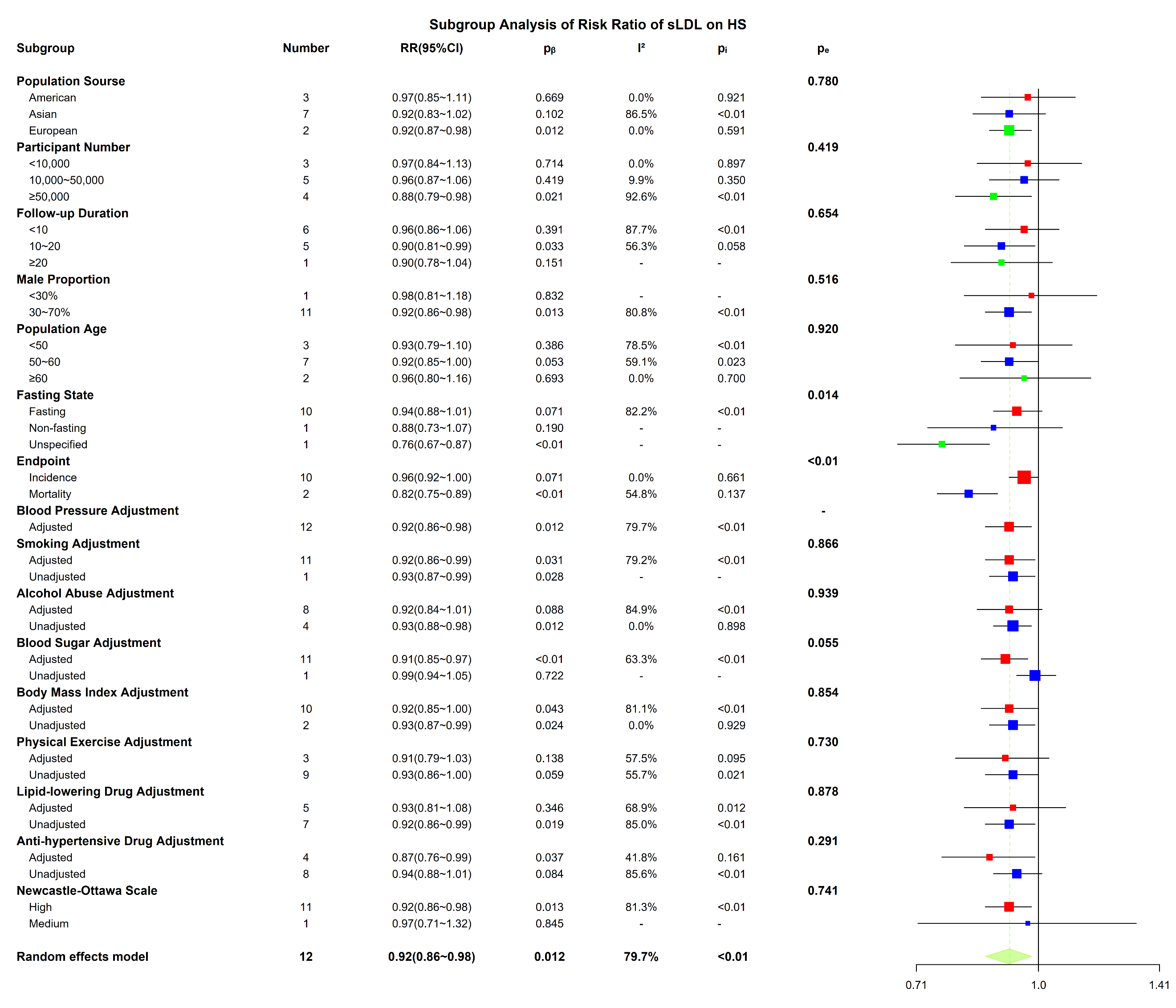


**Supplemental Figure 9** Continuous variable analysis on relationship between serum low-density lipoprotein cholesterol and risk of intracerebral hemorrhage; **A)** Baujat plot for heterogeneity analysis, **B)** Funnel plot for bias on publication, **C)** Funnel plot for bias on publication after trim-and-fill method, **D)** Bubble plot for meta-regression on publish years, **E)** Forest plot for risk ratio after trim-and-fill method, **F)** Forest plot for risk ratio after one-by-one exclusion.


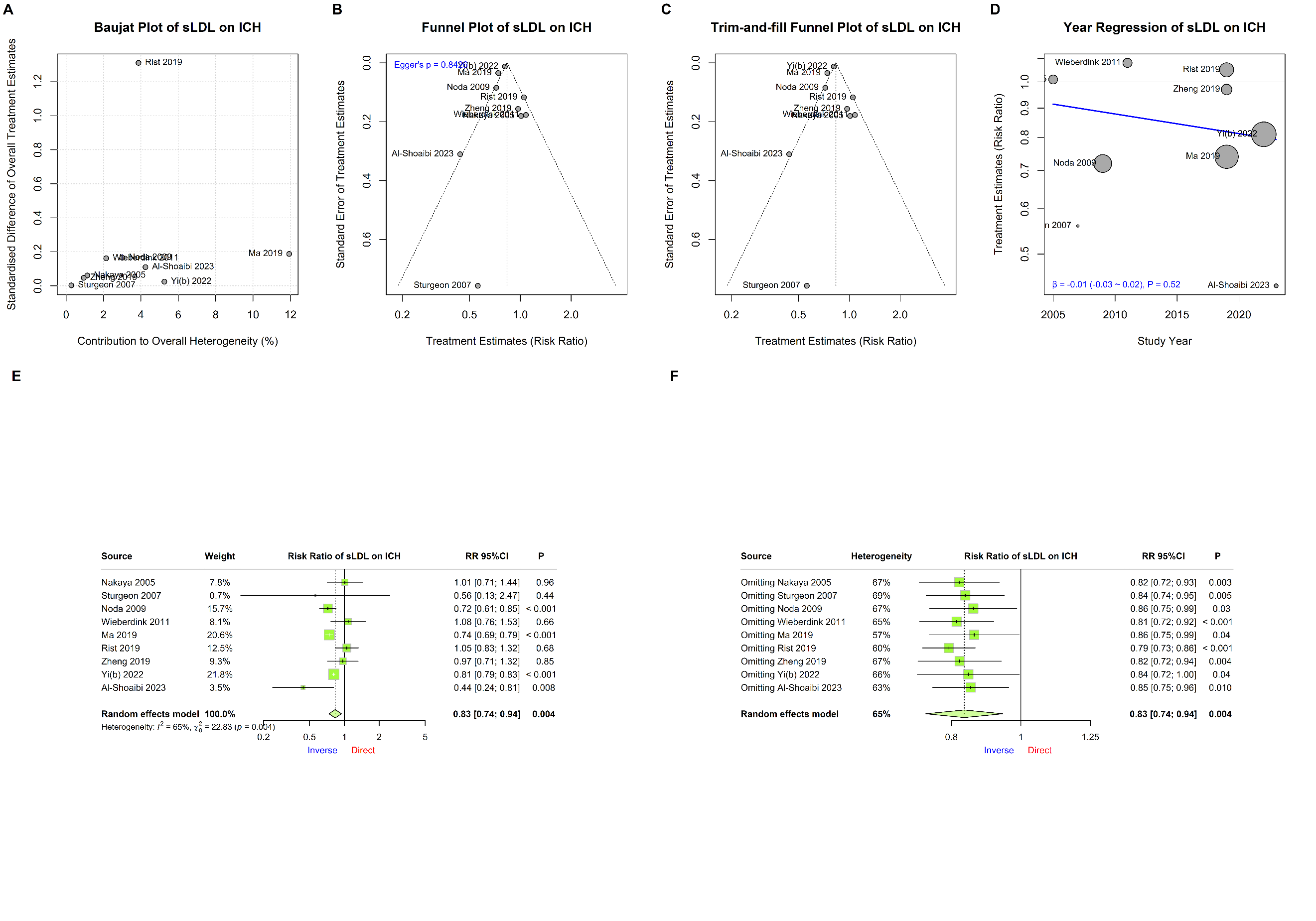


**Supplemental Figure 10** Forest plots of subgroup analysis of continuous variable analysis on risk ratio of serum low-density lipoprotein cholesterol on intracerebral hemorrhage. p_β_ represents *P* value for treatment estimates within subgroups; p_i_ represents *P* value for heterogeneity within subgroups; p_e_ as represents *P* value for heterogeneity between subgroups.


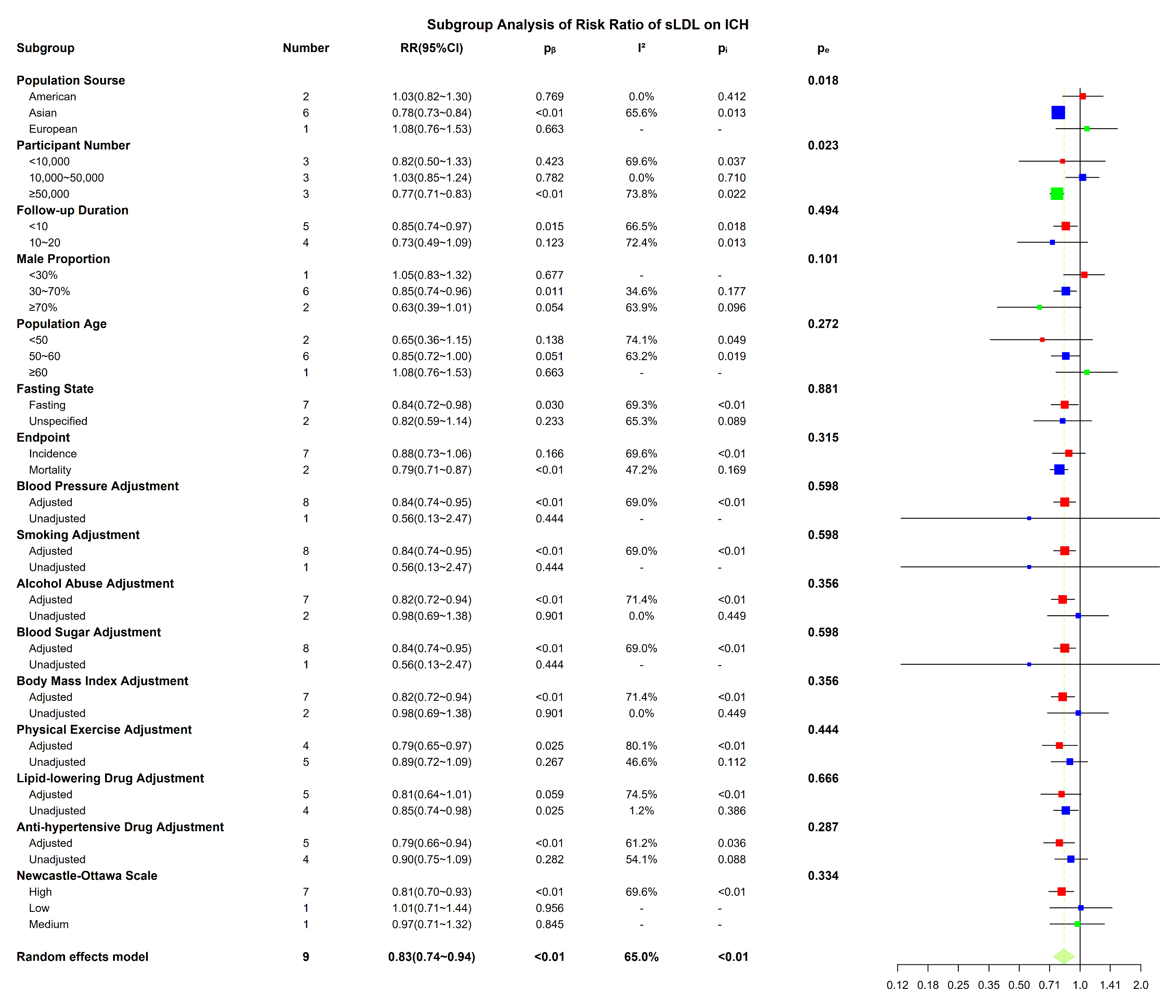


**Supplemental Figure 11** Continuous variable analysis on relationship between serum high-density lipoprotein cholesterol and risk of total hemorrhage stroke; **A)** Baujat plot for heterogeneity analysis, **B)** Funnel plot for bias on publication, **C)** Funnel plot for bias on publication after trim-and-fill method, **D)** Bubble plot for meta-regression on publish years, **E)** Forest plot for risk ratio after trim-and-fill method, **F)** Forest plot for risk ratio after one-by-one exclusion.


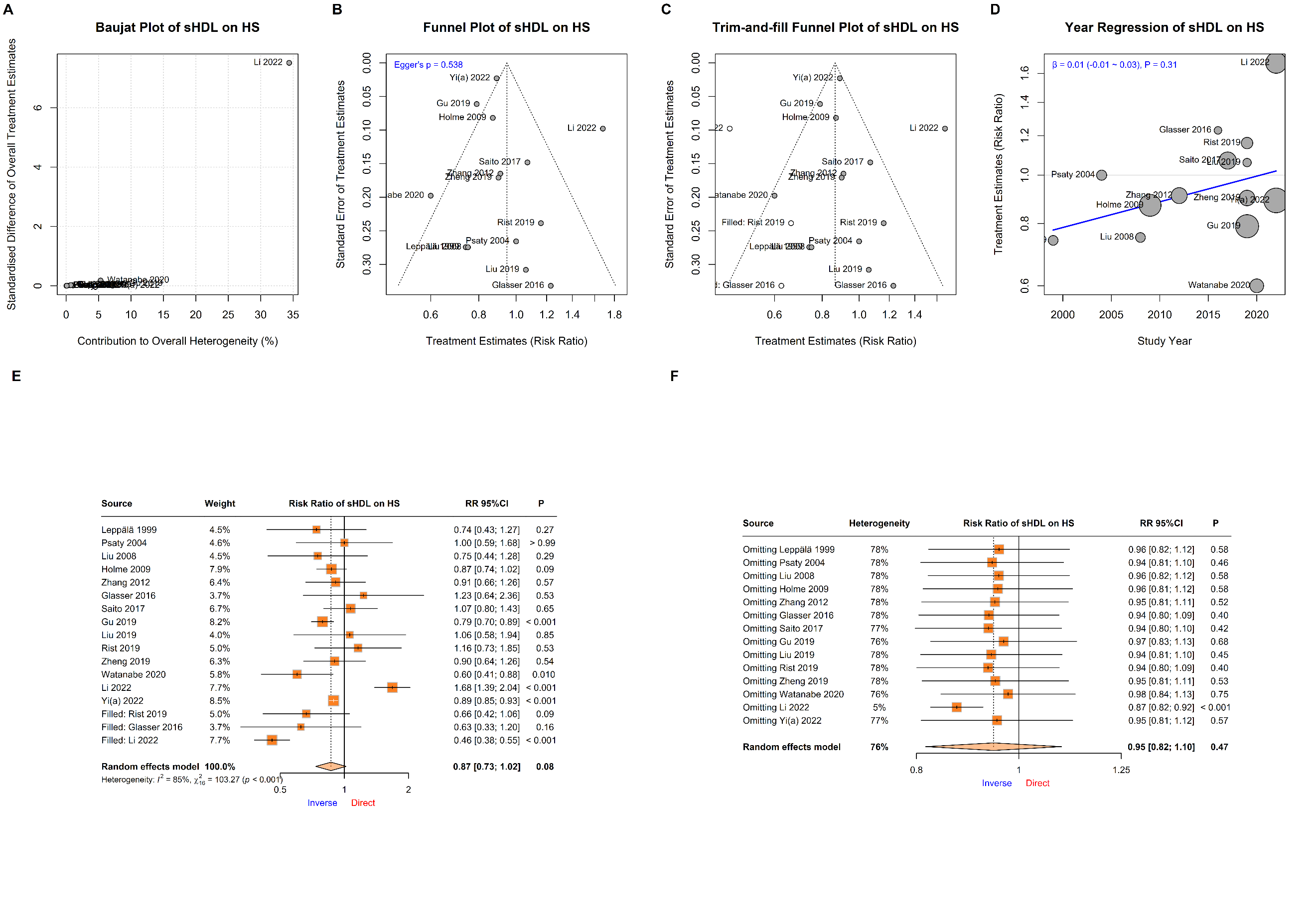


**Supplemental Figure 12** Forest plots of subgroup analysis of continuous variable analysis on risk ratio of serum high-density lipoprotein cholesterol on total hemorrhagic stroke. p_β_ represents *P* value for treatment estimates within subgroups; p_i_ represents *P* value for heterogeneity within subgroups; p_e_ as represents *P* value for heterogeneity between subgroups.


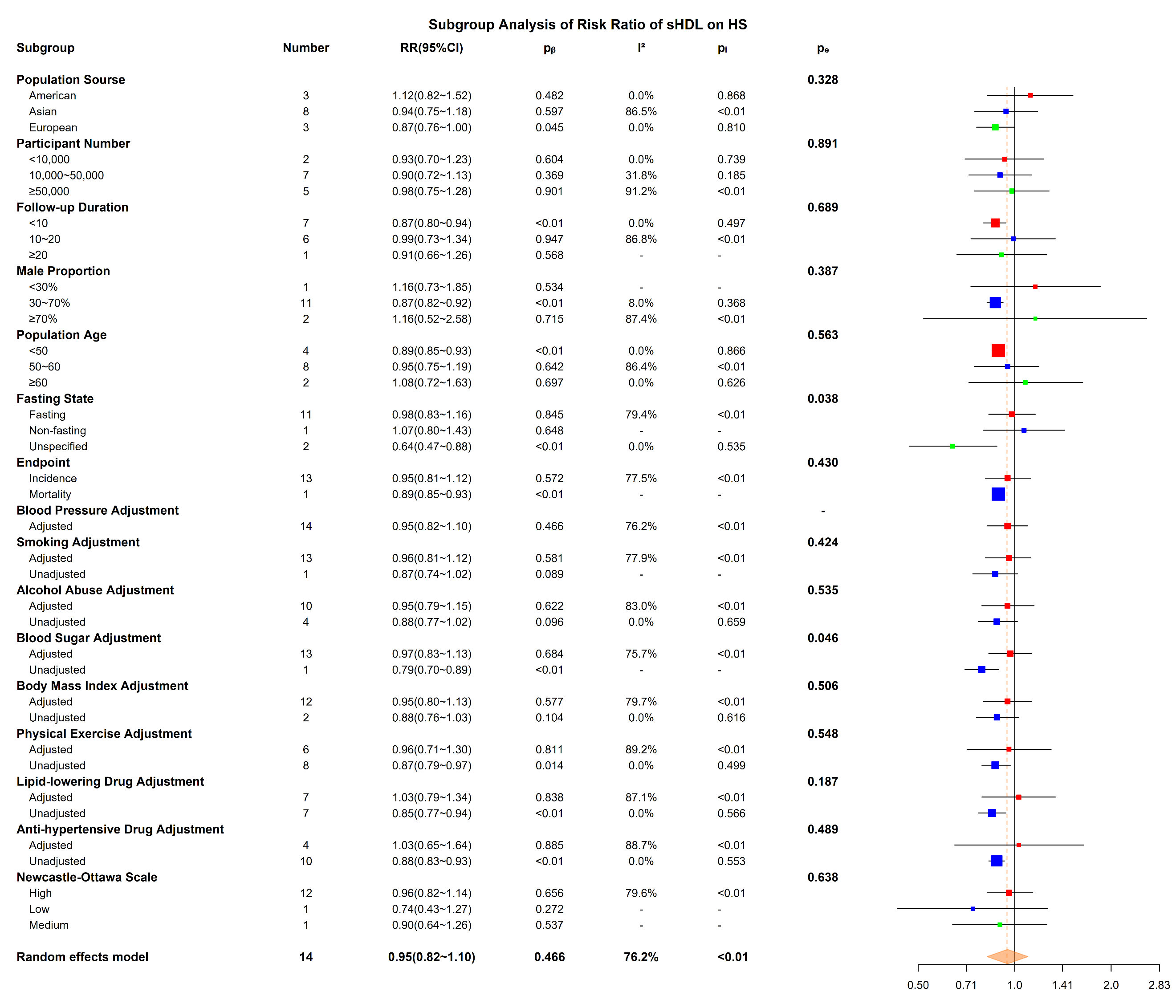


**Supplemental Figure 13** Continuous variable analysis on relationship between serum high-density lipoprotein cholesterol and risk of intracerebral hemorrhage; **A)** Baujat plot for heterogeneity analysis, **B)** Funnel plot for bias on publication, **C)** Funnel plot for bias on publication after trim-and-fill method, **D)** Bubble plot for meta-regression on publish years, **E)** Forest plot for risk ratio after trim-and-fill method, **F)** Forest plot for risk ratio after one-by-one exclusion.


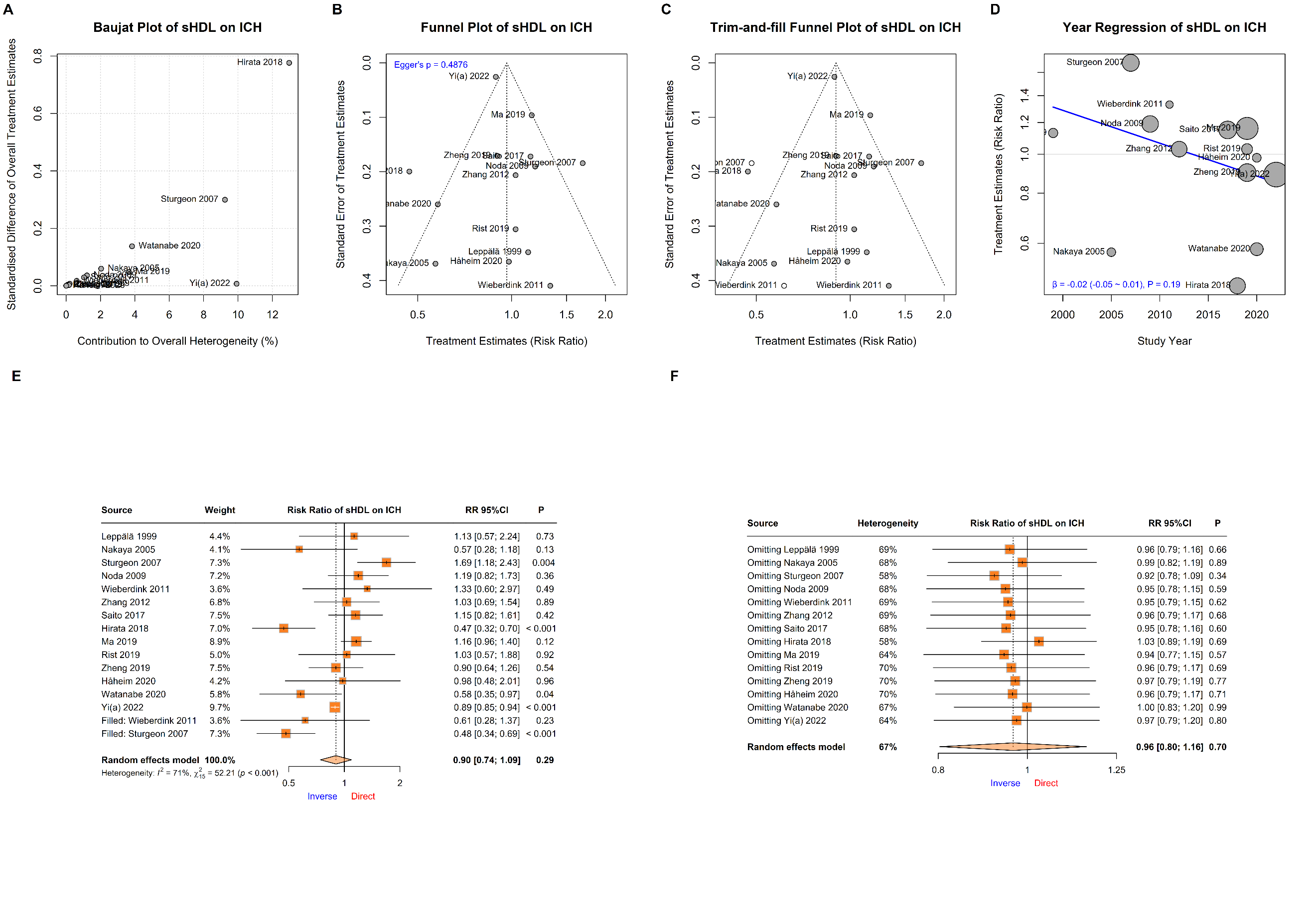


**Supplemental Figure 14** Forest plots of subgroup analysis of continuous variable analysis on risk ratio of serum high-density lipoprotein cholesterol on intracerebral hemorrhage. p_β_ represents *P* value for treatment estimates within subgroups; p_i_ represents *P* value for heterogeneity within subgroups; p_e_ as represents *P* value for heterogeneity between subgroups.


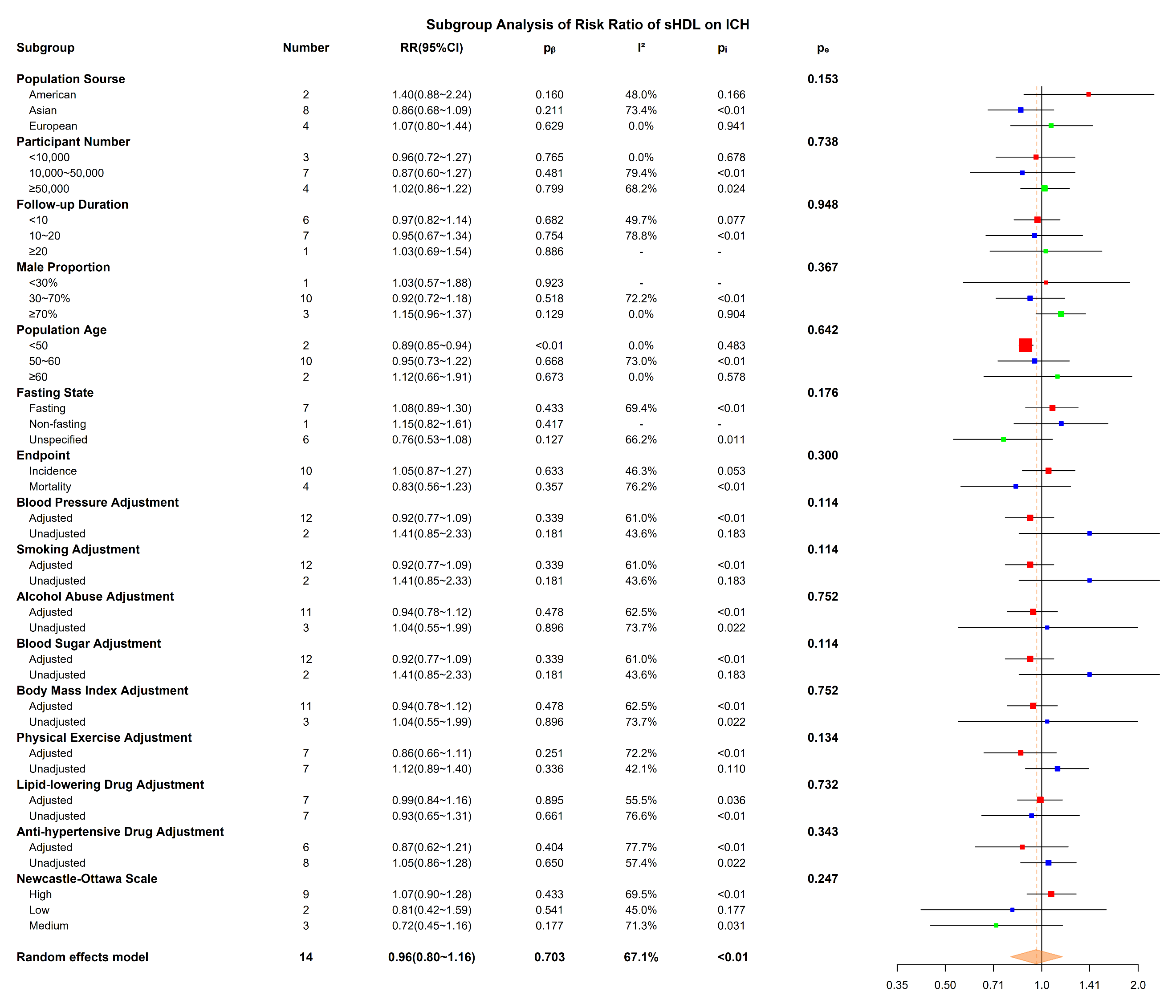


**Supplemental Figure 15** Continuous variable analysis on relationship between serum high-density lipoprotein cholesterol and risk of subarachnoid hemorrhage; **A)** Baujat plot for heterogeneity analysis, **B)** Funnel plot for bias on publication, **C)** Funnel plot for bias on publication after trim-and-fill method, **D)** Bubble plot for meta-regression on publish years, **E)** Forest plot for risk ratio after trim-and-fill method, **F)** Forest plot for risk ratio after one-by-one exclusion.


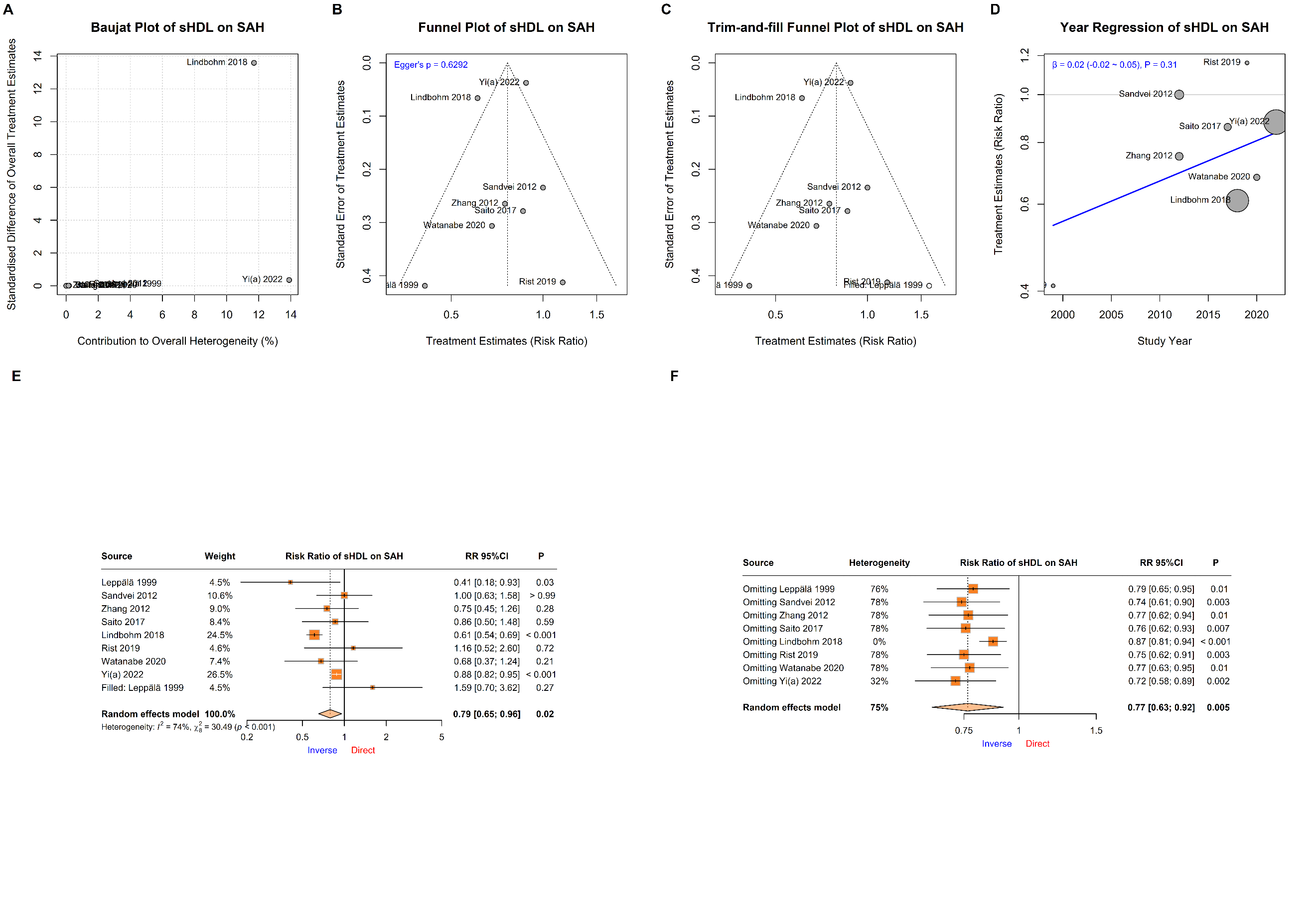


**Supplemental Figure 16** Forest plots of subgroup analysis of continuous variable analysis on risk ratio of serum high-density lipoprotein cholesterol on subarachnoid hemorrhage. p_β_ represents *P* value for treatment estimates within subgroups; p_i_ represents *P* value for heterogeneity within subgroups; p_e_ as represents *P* value for heterogeneity between subgroups.


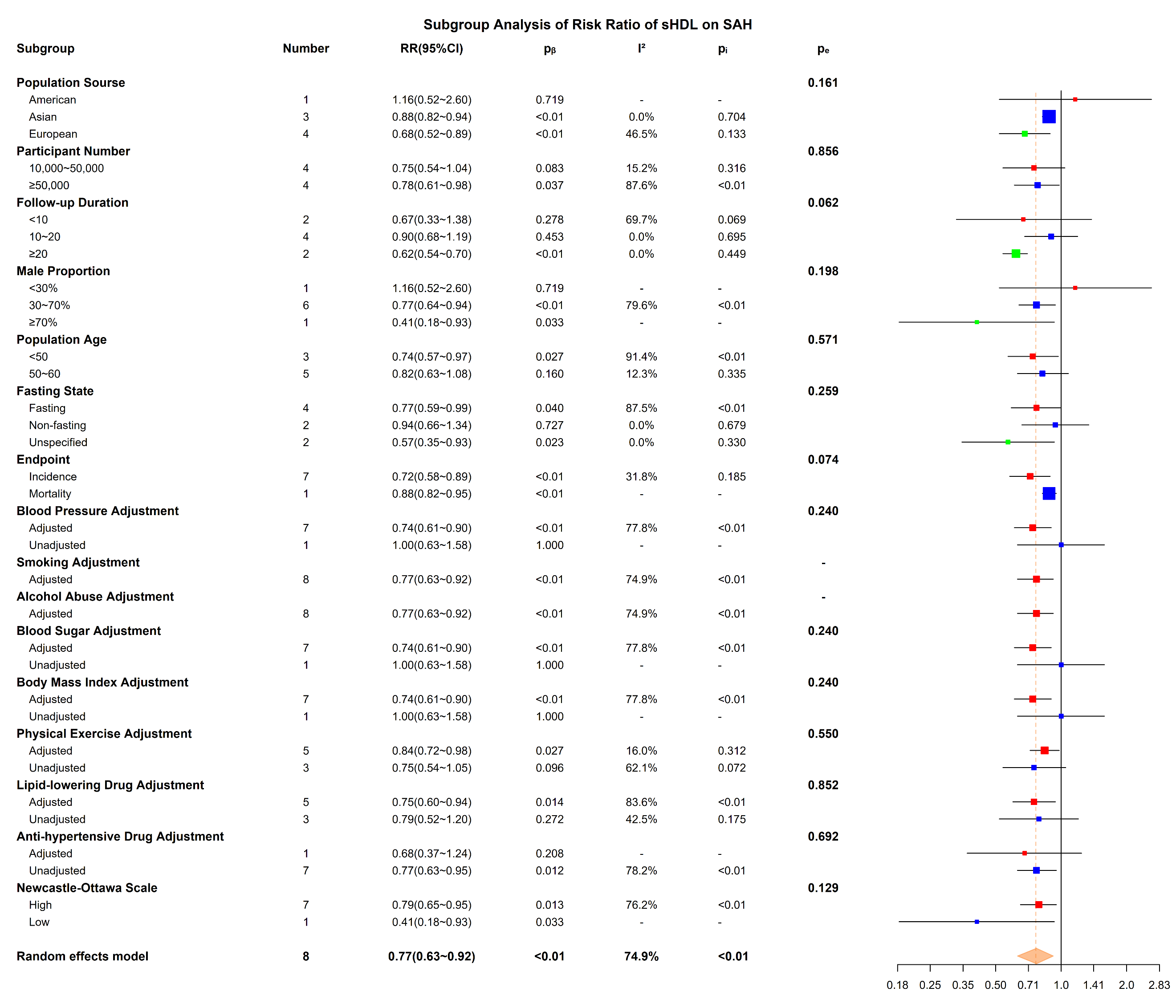


**Supplemental Figure 17** Continuous variable analysis on relationship between serum triglyceride and risk of total hemorrhage stroke; **A)** Baujat plot for heterogeneity analysis, **B)** Funnel plot for bias on publication, **C)** Funnel plot for bias on publication after trim-and-fill method, **D)** Bubble plot for meta-regression on publish years, **E)** Forest plot for risk ratio after trim-and-fill method, **F)** Forest plot for risk ratio after one-by-one exclusion.


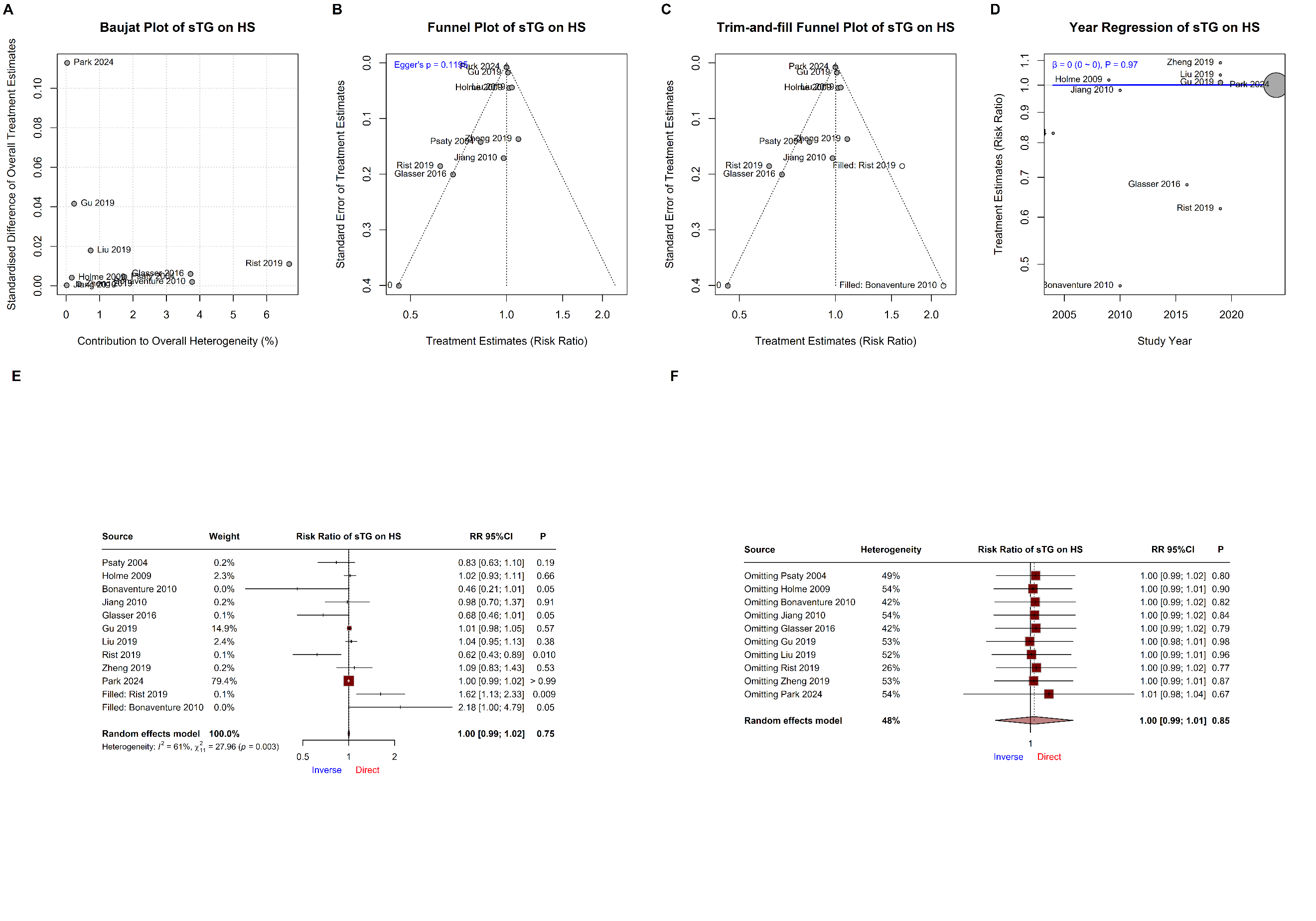


**Supplemental Figure 18** Forest plots of subgroup analysis of continuous variable analysis on risk ratio of serum triglyceride on total hemorrhagic stroke. p_β_ represents *P* value for treatment estimates within subgroups; p_i_ represents *P* value for heterogeneity within subgroups; p_e_ as represents *P* value for heterogeneity between subgroups.


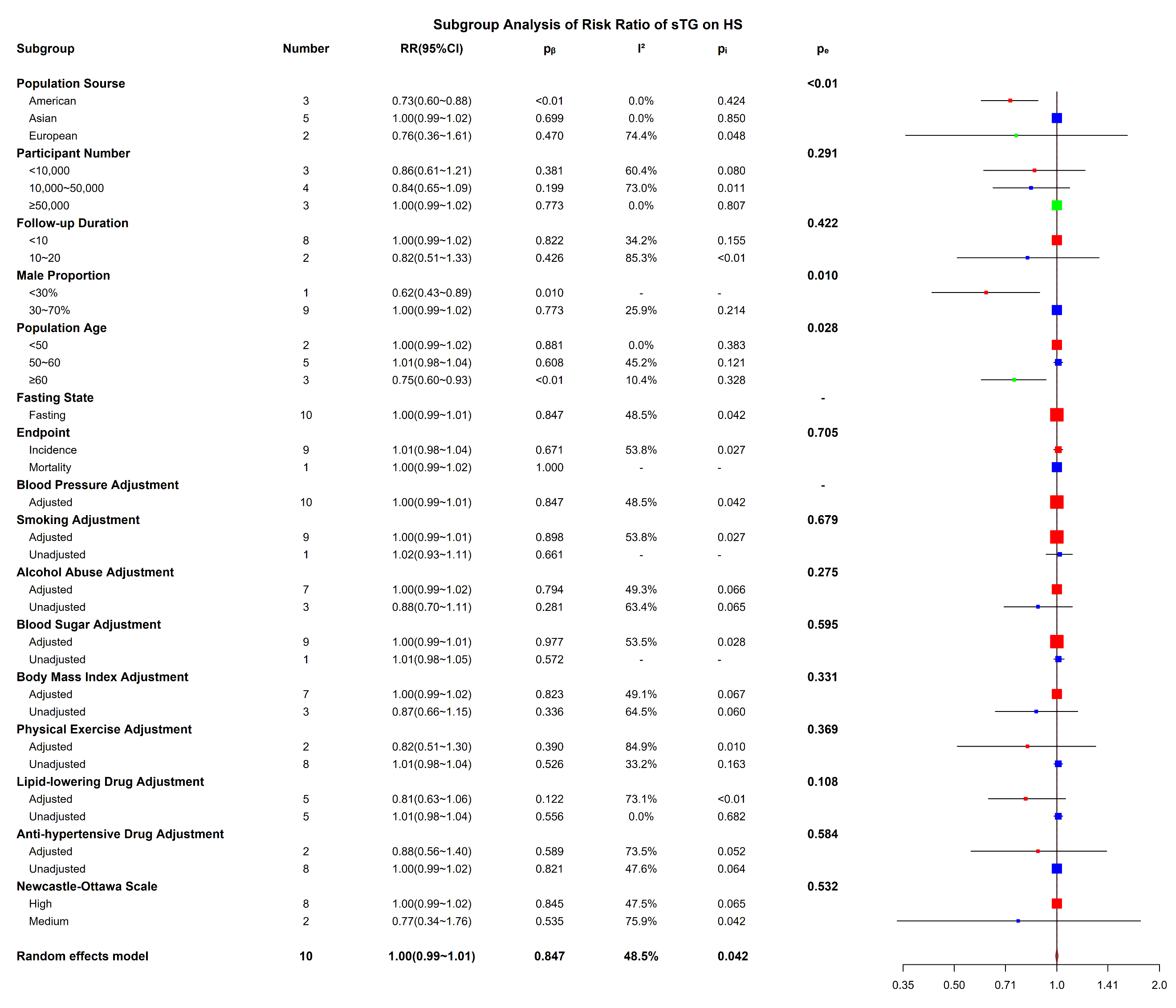


**Supplemental Figure 19** Continuous variable analysis on relationship between serum triglyceride and risk of intracerebral hemorrhage; **A)** Baujat plot for heterogeneity analysis, **B)** Funnel plot for bias on publication, **C)** Funnel plot for bias on publication after trim-and-fill method, **D)** Bubble plot for meta-regression on publish years, **E)** Forest plot for risk ratio after trim-and-fill method, **F)** Forest plot for risk ratio after one-by-one exclusion.


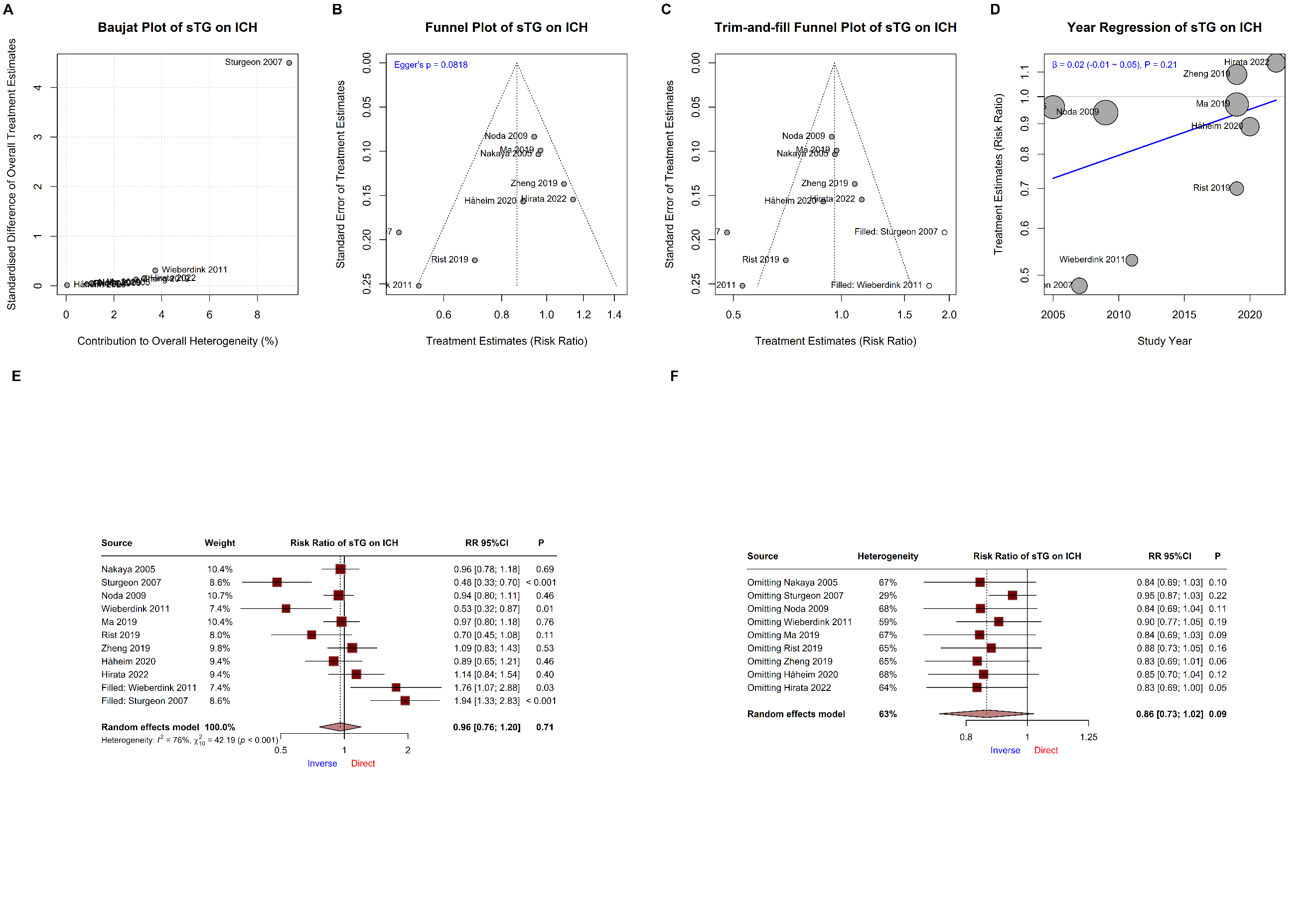


**Supplemental Figure 20** Forest plots of subgroup analysis of continuous variable analysis on risk ratio of serum triglyceride on intracerebral hemorrhage. p_β_ represents *P* value for treatment estimates within subgroups; p_i_ represents *P* value for heterogeneity within subgroups; p_e_ as represents *P* value for heterogeneity between subgroups.


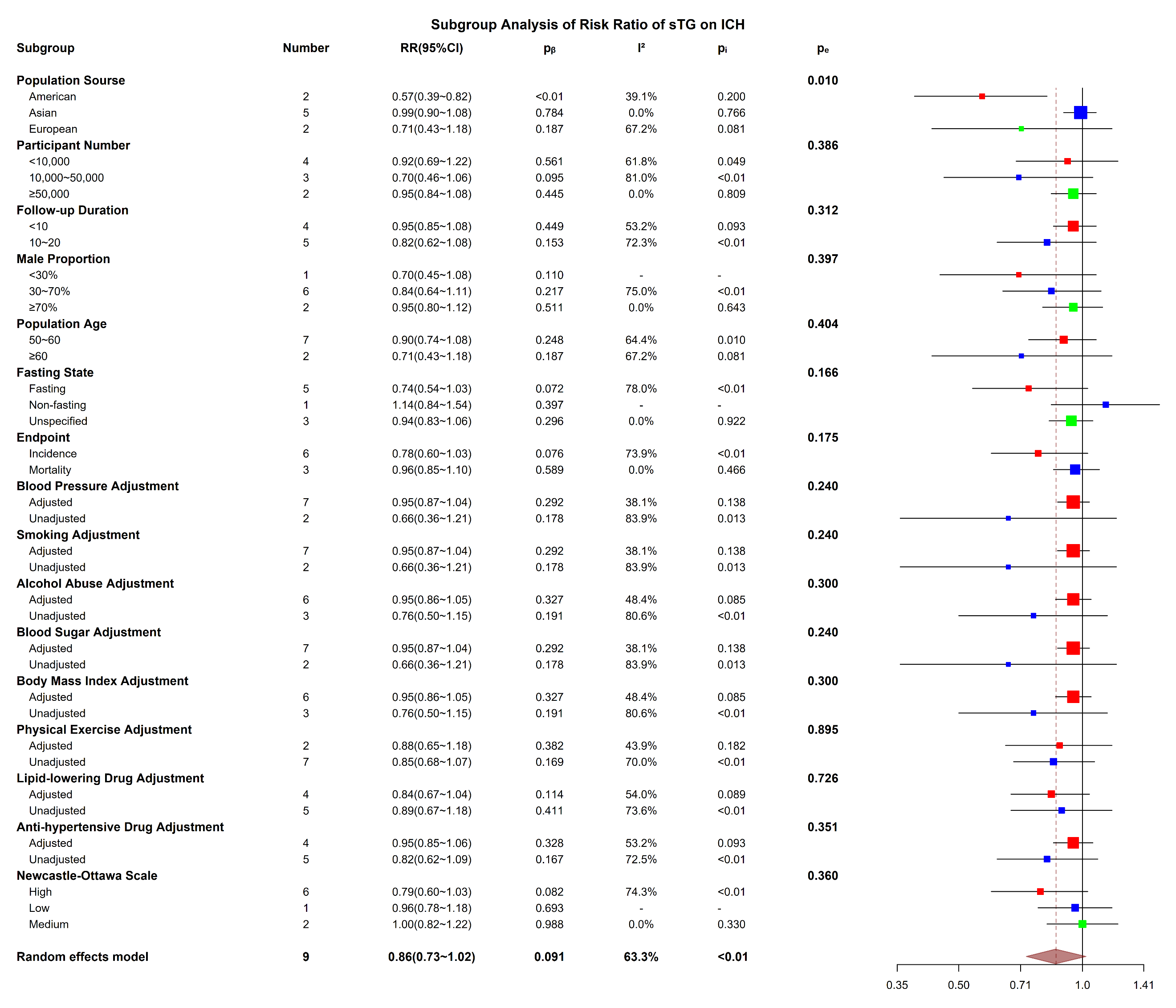

Supplement: Supplementary file 1 — Supplementary Material 1. [file 12944_2025_2698_MOESM1_ESM.docx]
